# Transfer Learning via Regularized Random-effects Linear Discriminant Analysis

**Published:** 2026

**Authors:** Hongzhe Zhang, Arnab Auddy, Hongzhe Li

**Affiliations:** Department of Biostatistics, Epidemiology and Informatics, University of Pennsylvania, Philadelphia, PA 19104, USA; Department of Statistics, The Ohio State University, Columbus, OH 43210, USA; Department of Biostatistics, Epidemiology and Informatics, University of Pennsylvania, Philadelphia, PA 19104, USA

**Keywords:** Covariate shift, Estimation risk, High dimension, Prediction risk, Random matrix theory, Regularization

## Abstract

Linear discriminant analysis is a widely used method for classification. However, the high dimensionality of predictors combined with small sample sizes often results in large classification errors. To address this challenge, it is crucial to leverage data from related source models to enhance the classification performance of a target model. This paper proposes a transfer learning approach via regularized random-effects linear discriminant analysis, where the discriminant direction is estimated as a weighted combination of ridge estimates obtained from both the target and source models. Multiple strategies for determining these weights are introduced and evaluated, including one that minimizes the estimation risk of the discriminant vector and another that minimizes the classification error. Utilizing results from random matrix theory, we explicitly derive the asymptotic values of these weights and the associated classification error rates in the high-dimensional setting, where the aspect ratio γ≔p/n as p,n→∞, with p representing the predictor dimension and n the sample size. Extensive numerical studies, including simulations, the analysis of the proteomics-based cardiovascular disease risk classification and the lipid traits classification problem with genotype data, demonstrate the effectiveness of the proposed approach.

## 1 Introduction

Large and diverse data sets are ubiquitous in modern applications, including those in genomics and medical decisions. It is of significant interest to integrate different data sets to obtain more accurate parameter estimates or to make a more accurate prediction or classification of an outcome. The success of a supervised statistical learning method relies on the availability of training data. When data is scarce, choosing an appropriately flexible model becomes critical to achieving optimal prediction accuracy. This is a classic illustration of the well-known “bias-variance tradeoff”. When building a prediction model for a target population, many auxiliary source data sets may exist and provide additional information for building the model for the target population. Modern techniques in the field of transfer learning (Pan and Yang, 2009; Weiss et al., 2016) aim to exploit these additional information. Given a target problem to solve, transfer learning (Torrey and Shavlik, 2010) aims at transferring the knowledge from different but related samples or studies to improve the learning performance of the target problem. In biomedical studies, some clinical or biological outcomes are hard to obtain due to ethical or cost issues, in which case transfer learning can be leveraged to boost the prediction and estimation performance by effectively utilizing information from related studies. Other relevant approaches include meta-learning (Peng, 2020; Huisman et al., 2021), domain adaptation (Redko et al., 2020; Sun et al., 2015), and, more recently, continual learning (De Lange et al., 2021).

In the high dimensional setting, Li et al. (2022, 2023) developed transfer learning methods for sparse high dimensional regressions and demonstrated that one can improve predictions of gene expression levels using data across different tissues. Such sparse models work well when the true models are sparse and the sample sizes are large. However, there are settings where sparse model assumption may not be valid. In genetics, estimating polygenetic risk scores (prss) using genome-wide genotype data (Mak et al., 2017; Torkamani et al., 2018) is an active area of research. Such prss can be used in risk stratification, or can be treated as risk factors in population health studies. However, due to very large number of genetic variants but relatively small sample sizes, building a prs model that accurately predicts the prs scores is challenging. Alternatively, ridge regression, which does not require the sparseness assumption, but can handle the linkage disequilibrium among the genetic variants, provides a viable method for prs prediction. Zhang and Li (2023) studied the estimation and prediction of random coefficient ridge regression in the setting of transfer learning, where in addition to observations from the target model, source samples from different but possibly related regression models are available. The informativeness of the source model to the target model can be quantified by the correlation between the regression coefficients.

The method of Zhang and Li (2023) is developed for continuous outcomes. In this paper, we propose a transfer learning framework for regularized linear discriminant analysis (RDA) (Friedman, 1989), which we term TL-RDA. Our model assumes a random classification weights setup, where the means of the covariates between two classes differ by a random quantity, δ, with zero mean and constant variance. The auxiliary and target populations are related through the correlation structure of δ. The TL-RDA framework aims to estimate the Bayes optimal predictor by combining naive RDA estimates from both the auxiliary and target populations through a weighted summation. The weights are designed to minimize the distance between the TL-RDA estimator and the Bayes optimal direction in a high-dimensional setting, where the number of features p grows proportionally to the sample size n in all populations. We derive the explicit asymptotic error rate for TL-RDA and show that it achieves the lowest error rate among all estimators based on weighted summations, including naive RDA using only target population data.

Several other LDA–style transfer learning methods have been proposed, but differ in the way of incorporating the source discriminant information. Yang and Gao (2013) derive the multi-view discriminant vectors through a single generalized-eigenvalue problem that maximizes the inter-view correlation while reducing domain discrepancy. Wang et al. (2020) imposes a group-sparse Bayesian prior on discriminant weights to select and linearly combine the informative source domains. Han et al. (2020); Liu et al. (2023) reformulate the classical LDA to learn explicitly the domain-invariant projections. They also augment Fisher's between-/within-class scatter ratio with a distribution-alignment regularizer that forces the projected source and target means and covariances to match. However, none of these methods has been analyzed in the high-dimensional random effects regime, and none aggregates source information by explicitly minimizing the target-domain prediction risk; which are two aspects that are central to our proposed TL-RDA.

A separate stream of work chooses to fuse or shrink parameters across populations with different objectives. In multi-task learning frameworks, discriminant directions or regression coefficients are encouraged to be close by adding fused penalties (Price and al., 2014; Okazaki and Aoyagi, 2024; Lozano and Swirszcz, 2012). These methods minimize a joint loss over all tasks, seeking a model that performs reasonably well simultaneously across domains. Komárek and Lesaffre (2010) proposes a related random-effects LDA that pools information through hierarchical priors from a Bayesian perspective. In the domain-adaptation literature, hypothesis-weighting techniques combine pre-trained source classifiers by shrinking the weights toward a simplex that balances average source risk (Mansour et al., 2009). Closer in spirit to our work, Gu et al. (2024) moves a high-dimensional ridge estimate toward multiple source coefficients along geodesic paths, with step-sizes chosen to minimize an asymptotic proxy for the target prediction error. Their analysis, however, is confined to linear regression and assumes access only to fitted source coefficients. A complementary direction is to bypass parameter fusion and instead learn latent features whose label–relationship is invariant across environments (Peters et al., 2016; Arjovsky et al., 2019). Such invariance-seeking objectives inevitably trade off target-specific accuracy, while TL-RDA tailors an optimized discriminant vector for the single target population.

The remainder of the paper is organized as follows. We first provide a detailed problem setup and outline the proposed TL-RDA approach in Section 2. We then introduce various types of weights used to integrate auxiliary information, followed by the technical assumptions required for analyzing the estimator in the context of random matrix theory in Section 3. In Section 4, we present the analysis of TL-RDA in the proportional regime, providing explicit asymptotic expressions for the different weighting schemes, along with their corresponding error rates. Section 5 offers interpretations of these weights and guidance on how users can select the most appropriate weights for their applications. In the first five sections, we have assumed all populations share the same population covariance matrix. Section 6 extends the TL-RDA to a heterogeneous population covariance matrix set up. In Section 7, we evaluate the proposed methods on two tasks: (i) cardiovascular-disease classification using proteomics data from the Chronic Renal Insufficiency Cohort (CRIC), and (ii) binary lipid traits prediction using gene-expression data from the Penn Medicine BioBank (PMBB). Section 8 offers a brief discussion.

## 2 Transfer Learning via Regularized Discriminant Analysis

We consider the setting of two-class LDA in the setting of transfer learning, where we have data observed from both the target model, indexd by K, and K-1 source models, indexed by k=1,⋯,K-1. We assume that all models, k=1,⋯,K, follow the classic two-class Gaussian mixture model. More specifically, for $i=1,\ldots ,n_{k}$ and k=1,…,K,

(1) $$y_{k}\in \left\{-1,+1\right\}\;P\left(y_{k}=\pm 1\right)=\pi_{\pm 1}\;{\left(X_{k}\right)}_{i}|y_{k}\sim N\left(\mu_{y,k},\Sigma \right).$$

Here ${\left(X_{k}\right)}_{i},i=1,\cdots ,n_{k}$ is a p–dimensional vector, and for ease of notation, we write $X_{k}={\left({\left(X_{k}\right)}_{1}{\left(X_{k}\right)}_{2}\ldots {\left(X_{k}\right)}_{n_{k}}\right)}^{⊤}\in R^{n_{k}\times p}$ as a $n_{k}\times p$ dimensional matrix. For simplicity of notation, we assume that the mixing proportions $\pi_{\pm 1}$ remain the same across populations. In fact, without much loss of generality, we mainly discuss the simpler case $\pi_{-1}=\pi_{+1}=1/2$. The more general case can be managed in a similar manner without significant technical difficulty (Appendix C). Moreover, in most of the paper, we assume the population covariance matrix are the same for all K populations, and are denoted by Σ. This assumption is relaxed in Section 6 where we allow each population to have their own covariance matrix $\Sigma_{k}$ for k=1,…,K.

Under this set up, the Bayes optimal prediction direction for the target population K (Anderson, 1958) is given by,

$$d_{\text{Bayes}}≔\Sigma^{-1}\delta_{K}\;\text{where}\;{\overset{‾}{\mu }}_{K}=\frac{\mu_{+1,K}+\mu_{-1,K}}{2}\;\text{for}\;\mu_{\pm 1,K}={\overset{‾}{\mu }}_{K}\pm \delta_{K},$$

and $\delta_{K}=\left(\mu_{+1,K}-\mu_{-1,K}\right)/2$. The Bayes prediction for a testing data point $x_{0}$ from population K is

$${\hat{y}}_{\text{Bayes}}\left(x_{0}\right)=\text{sign}\left[d_{\text{Bayes}}^{⊤}\left(x_{0}-{\overset{‾}{\mu }}_{K}\right)\right].$$

The regularized discriminant analysis (RDA) classifier uses an empirical version of the unkown Bayes direction. The RDA classifier for population k is a linear classifier

$${\hat{y}}_{RDA,k}\left(x_{0}\right)=\text{sign}\left({\hat{d}}_{k}^{⊤}x_{0}+{\hat{b}}_{k}\right),$$

where we use the plug-in estimates for the population parameters $\Sigma_{k}$ and $\delta_{k}$ as follows:

$${\hat{d}}_{k}={\left({\hat{\Sigma }}_{k}+\lambda_{k}I_{p}\right)}^{-1}{\hat{\delta }}_{k}$$

$${\hat{b}}_{k}=-{\hat{\delta }}_{k}^{⊤}{\left({\hat{\Sigma }}_{k}+\lambda_{k}I_{p}\right)}^{-1}\left({\hat{\mu }}_{-1,k}+{\hat{\mu }}_{+1,k}\right)/2$$

$${\hat{\mu }}_{\pm 1,k}=\frac{2}{n_{k}}\sum_{i:{\left(y_{k}\right)}_{i}=\pm 1}{\left(X_{k}\right)}_{i}\;{\hat{\delta }}_{k}=\frac{{\hat{\mu }}_{+1,k}-{\hat{\mu }}_{-1,k}}{2}$$

$${\hat{\Sigma }}_{k}=\frac{1}{n_{k}-2}\sum_{i=1}^{n_{k}}\left[{\left(X_{k}\right)}_{i}-{\hat{\mu }}_{{\left(y_{k}\right)}_{i},k}\right]{\left[{\left(X_{k}\right)}_{i}-{\hat{\mu }}_{{\left(y_{k}\right)}_{i},k}\right]}^{⊤}.$$

Here ${\hat{\Sigma }}_{k}$ is the usual sample covariance matrix, while ${\hat{\mu }}_{\pm 1,k},{\hat{\delta }}_{k}$ are simple estimators and the population-level counterpart $\mu_{\pm 1,k},\delta_{k}$. The sample covariance matrix is penalized on its diagonal to overcome the overwhelming variances in this estimation when p grows proportionally with n. As the penalization parameter $\lambda_{k}$ goes to zero or infinity, this classifier recover the Fisher's discriminant analysis or the naive Bayes method (Bickel and Levina, 2004).

To utilize the underlying relatedness of the RDA problems in K populations, for a vector of weights $w\in R^{K}$, we now define a classifier based on a weighted linear combination of the population specific discriminator vectors ${\hat{d}}_{k}\text{s}$ as follows:

$$\hat{d}\left(w\right)=\sum_{k=1}^{K}w_{k}{\hat{d}}_{k}.$$

Note that taking the co-ordinate basis vectors as weights, i.e., $w=e_{k}\in R^{K}$, we end up with the population specific discriminant directions $\hat{d}\left(e_{k}\right)={\hat{d}}_{k}$ for k=1,…,K. Now using the weighted combination discriminant vector we define the *transfer-learning* (TL) classifier

(2) $${\hat{y}}_{w}\left(x_{0}\right)=\text{sign}\left(\hat{d}(w{)}^{⊤}x_{0}+{\hat{b}}_{K}\right)$$

where $x_{0}\in R^{p}$ is a test point in the target population. We call this TL-RDA. We use the regular intercept term ${\hat{b}}_{K}$ as all intercept terms are asymptotically zero in the regime considered.

We formulate two criteria to be optimized over w. Let us recall the Bayes classification direction $d_{\text{Bayes}}$. Now for a testing data pair $\left(x_{0},y_{0}\right)$ sampled from population K, we consider the two errors:

(3) $${\left\|d_{\text{Bayes}}-\sum_{k=1}^{K}w_{k}{\hat{d}}_{k}\right\|}_{2}^{2},$$

(4) $$E_{x_{0}}{\left[{\left(d_{\text{Bayes}}-\sum_{k=1}^{K}w_{k}{\hat{d}}_{k}\right)}^{⊤}x_{0}\right]}^{2}.$$

The first criteria compares the transfer-learning classifier (TL-RDA) with the Bayes optimal classifier in terms of the estimation error, while the second criteria compares the two classifiers in terms of their prediction errors. In later sections, we present explicit solutions for optimal w that optimizes the two criteria above. Though none of (3) or (4) is explicitly related to prediction error rate, we show the optimal weight that minimizes (4) also minimizes the prediction error rate in the target population. We will also show the weights that minimizes these two criteria, are in fact related to one another.

In addition, we assume that the population covariance matrix are the same for all K populations (assumption 1). With this in mind, a natural way to further exploit mutual information across populations is to use a pooled sample covariance for all discriminant directions ${\hat{d}}_{k}$. We write ${\hat{\Sigma }}_{P}$ to denote the pooled sample covariance matrix

$${\hat{\Sigma }}_{P}=\sum_{k=1}^{K}\sum_{i=1}^{n_{k}}\left[{\left(X_{k}\right)}_{i}-{\hat{\mu }}_{y_{i},k}\right]{\left[{\left(X_{k}\right)}_{i}-{\hat{\mu }}_{y_{i},k}\right]}^{⊤}/\sum_{k=1}^{K}\left(n_{k}-2\right).$$

We then define the pooled classification weights and pooled transfer learning regularized discriminant analysis (TLP-RDA) estimator as

(5) $${\hat{y}}_{w}^{P}\left(x_{0}\right)=\text{sign}\left({\hat{d}}^{P}(w{)}^{⊤}x_{0}+{\hat{b}}_{K}\right)$$

where the direction estimates ${\hat{d}}_{k}^{P}$ are now estimated using the pooled covariance matrix as follows

$${\hat{d}}_{k}^{P}≔{\left({\hat{\Sigma }}_{P}+\lambda_{k}I_{p}\right)}^{-1}{\hat{\delta }}_{k}\;\text{and}\;{\hat{d}}^{P}\left(w\right)≔\sum_{k=1}^{K}w_{k}{\hat{d}}_{k}^{P}.$$

Once again using (3) and (4), we can optimize w in ${\hat{y}}_{w}^{P}\left(x_{0}\right)$ with respect to the two criteria above as well, and we will discuss how to choose between ${\hat{y}}_{w}^{P}\left(x_{0}\right)$ and ${\hat{y}}_{w}\left(x_{0}\right)$.

This form of TL estimator (2) has been used in Zhang and Li (2023) and Dobriban and Sheng (2020) for regression methods. Helm et al. (2024) also considers a TL estimator that aggregates auxiliary information by weighted summations of individual discriminant directions. However, their aggregation is less adaptive in the form $\hat{d}(w{)}^{*}=a\;{\hat{d}}_{K}+(1-a)\sum_{k=1}^{K-1}{\hat{d}}_{k}/(K-1)$ where a is a constant. In addition, the final weight a minimizes only empirical quantities and does not guarantee to minimize population-level criteria such as (3) or (4).

## 3 Random-effects RDA and the Assumptions on Population Parameters

To find the weights that minimize the estimation or prediction risk defined as 3 and 4, we consider random-effects RDA where the population means are random and are potentially correlated across different models. In addition, we also make the random matrix assumptions on the covariance matrix, which allows us to apply the recent advances in random matrix theory to derive the limiting values of the weights and the corresponding classification errors.

### 3.1 Classification Weights

A key parameter in the two-class classification problem is the classification weights $\delta_{k}$ that separate the two classes. In this paper, we consider a random classification weights set up formalized below.

**Assumption 1 (Random Classification Weights)** *The following conditions hold for populations* k=1,⋯,K.

1. *The class-specific population mean vectors* $\mu_{-1,k},\mu_{+1,k}\in R^{p}$ *are randomly generated as* $\mu_{\pm 1,k}={\overset{‾}{\mu }}_{k}\pm \delta_{k}$, *where each* $\delta_{k}$ *has i.i.d. coordinates with*
$$E\left({\left(\delta_{k}\right)}_{i}\right)=0\;\text{Var}\left({\left(\delta_{k}\right)}_{i}\right)=\alpha_{k}^{2}/p\;E\left({\left|{\left(\delta_{k}\right)}_{i}\right|}^{4+\eta }\right)\leq \frac{C}{p^{2+\eta }}$$
  *for fixed* C,η>0.
2. ${\overset{‾}{\mu }}_{k}$ *are either fixed or randomly distributed independent of* $\delta_{k},X_{k},y_{k}$. *The second moment of* ${\overset{‾}{\mu }}_{k}$ *are bounded almost surely such that* ${\text{lim}\;\text{sup}}_{p\to \infty }\left\|{\overset{‾}{\mu }}_{k}\right\|/p^{1/2-\xi }\leq C$ *for some constants* ξ,C>0.

The parameter $\alpha_{k}^{2}$ here plays the role of signal strength. This key assumption asserts that all coordinates of $\delta_{k}$ have the same zero mean and diminishing variance, therefore, all coordinates of an observation play equally important roles on determining the response class. This assumption also used by Dobriban and Wager (2018), is standard in the large n, large p regime. In addition, the bounded moment assumptions allow use to circumvent difficulties arise when minimizing criteria (3) and (4) which depends on unknown quantities such as $\delta_{k},\Sigma^{-1}$ by invoking Lemma A.5. The performance of the TL estimators clearly depends on how the K populations are related. We further pose the correlated classification weight assumption below.

**Assumption 2 (Correlated Classification Weights)** *For* k=1,⋯,K *the vectors* $\delta_{k}$ *are correlated across populations such that* $\text{Corr}\left(\delta_{k},\delta_{k^{\prime }}\right)=\rho_{kk^{\prime }}I_{p},k\neq k^{\prime }$.

For most of the paper, we assume that the parameters $\alpha_{k}^{2}$ and $\rho_{k,k^{\prime }}$ are known constants. We discuss consistent estimators for these parameters in Appendix B.3.

### 3.2 Random Matrix Assumption and Related Results

The sample covariance matrix is an important part of the transfer learning estimator. We consider the Marchenko-Pastur type sample covariance matrices as in several relevant works (Dobriban and Wager, 2018; Zhao et al., 2023; Zhang and Li, 2023). For a symmetric matrix, we can characterize its spectral distribution by a cumulative distribution function that places equal point mass on its eigenvalues. Our asymptotic analysis is based on the convergences of spectral distributions of sample covariance matrices, for which the following assumptions are required.

**Assumption 3 (rmt assumption)** *For* k=1,⋯,K, *the design matrix* $X_{k}\in R^{n_{k}\times p}$ *is generated as*

$$X_{k}={\left(\mu_{{\left(y_{k}\right)}_{1},k}\mu_{{\left(y_{k}\right)}_{2},k}\cdots \mu_{{\left(y_{k}\right)}_{n_{k}},k}\right)}^{⊤}+Z_{k}\Sigma^{1/2}$$

*for a matrix* $Z_{k}\in R^{n_{k}\times p}$ *with i.i.d. entries coming from an infinite array. The entries* ${\left(Z_{k}\right)}_{ij}$ *of* $Z_{k}$ *satisfy the moment conditions:* $E\left[{\left(Z_{k}\right)}_{ij}\right]=0$ *and* $E\left[{\left(Z_{k}\right)}_{ij}^{2}\right]=1$.

1. *The population covariance matrix* $\Sigma \in R^{p\times p}$ *is deterministic. The observations have unit variance, i.e*., $\Sigma_{jj}=1$ *for* j=1,⋯,p.
2. *The eigenvalues of* Σ *are uniformly bounded from above and away from zero with constants independent of the dimension* p.
3. *The sequence of spectral distributions* $T≔T_{\Sigma ,p}$ *of* $\Sigma ≔\Sigma_{p}$ *converges weakly to a limiting distribution* H *supported on* [0,∞), *called the population spectral distribution* (psd).

For sample covariance $\hat{\Sigma }≔(X-EX{)}^{⊤}(X-EX)/n$ with $X\in R^{n\times p}$ generated following the Assumption 3, its empirical spectral distribution (esd) is the probability measure

$$F^{\hat{\Sigma }}\left(\omega \right)=\frac{1}{p}\sum_{i=1}^{p}1\left\{\lambda_{i}\left(\hat{\Sigma }\right)\leq \omega \right\},\omega \in R,$$

where $\lambda_{i}(\hat{\Sigma })$ is the $i^{\text{th}}$ eigenvalue of $\hat{\Sigma }$. The Marchenko-Pastur theorem (Marchenko and Pastur, 1967) claims that $F^{\hat{\Sigma }}$ converges weakly (in distribution) to a limiting distribution $F_{\gamma }≔F_{\gamma }(H)$ supported on [0,∞) with probability 1, where γ is the aspect ratio defined as the limit of p/n→γ as p,n→∞.

Furthermore, for any distribution G supported on [0,∞), we define its Stieltjes transform as

(6) $$m_{G}\left(z\right)≔\int_{l=0}^{\infty }\frac{dG\left(l\right)}{l-z},\;z\in C\setminus R^{+}.$$

The esd of sample covariance matrix is uniquely determined by a fixed-point equation for its Stieltjes transform. The limit of the Stieltjes transform for the ESD is given by:

(7) $$m_{F^{\hat{\Sigma }}}(z)=\text{tr}\left\{{\left(\hat{\Sigma }-zI_{p}\right)}^{-1}/p\right\}\to_{a.s.}m_{F_{\gamma }}(z).$$

This can be understood by noting that the Stieltjes transform is a smooth, continuous functional of the esd and the esd converges weakly to $F_{\gamma }$.

We continue to define the Stieltjes transform of the limiting spectral distribution of $\overset{‾}{\Sigma }≔(X-EX)(X-EX{)}^{⊤}/p$ as $v_{F_{\gamma }}(z)$, called companion Stieltjes transform. For all $z\in C\setminus R^{+}$, the Stieltjes transform $v_{F_{\gamma }}(z)$ is related to $m_{F_{\gamma }}(z)$ by

(8) $$\gamma \left[m_{F_{\gamma }}(z)+\frac{1}{z}\right]=v_{F_{\gamma }}(z)+\frac{1}{z}$$

where γ is the limiting aspect ratio. In addition, we denote by $m_{F}^{\prime }(-\lambda )$ the derivative of the Stieltjes transform $m_{F}(z)$ evaluated at z=-λ, where

(9) $$m_{F_{\gamma }}^{\prime }(z)=\int_{l=0}^{\infty }\frac{dG(l)}{(l-z{)}^{2}}\;v_{F_{\gamma }}^{\prime }(z)=\gamma \left(m_{F_{\gamma }}^{\prime }(z)-\frac{1}{z^{2}}\right)+\frac{1}{z^{2}}.$$

In terms of the empirical quantities, similarly we have

$$m_{F^{\hat{\Sigma }}}^{\prime }(z)=\text{tr}\left\{{\left(\hat{\Sigma }-zI_{p}\right)}^{-2}/p\right\}\to_{a.s.}m_{F_{\gamma }}^{\prime }(z).\;(\text{Bai and Silverstein},2010)$$

These convergences form the bases on which we develop the limiting error rate and the limiting optimal weights according to criteria (3) and (4).

## 4 Asymptotic Analysis of Weights and Classification Errors

In this section, we present the expressions for the limiting prediction erorrs and the optimal weights. All formulae are compared with simulated data, and in Appendix B, they are demonstrated to be accurate even under small data sizes.

### 4.1 Classification Error

Under the two-class Gaussian classification model (Assumption 1), the expected test error of the linear classifier TL-RDA under weight w in target population K can be written as

$$Err(w)=\pi_{K,-}\Phi \left(\frac{(\hat{d}(w){)}^{⊤}\mu_{-1,K}+{\hat{b}}_{K}}{\sqrt{\left(\hat{d}(w){)}^{⊤}\Sigma (\hat{d}(w)\right)}}\right)+\pi_{K,+}\Phi \left(-\frac{(\hat{d}(w){)}^{⊤}\mu_{1,K}+{\hat{b}}_{K}}{\sqrt{\left(\hat{d}(w){)}^{⊤}\Sigma (\hat{d}(w)\right)}}\right)$$

$$\hat{d}(w)≔\sum_{k=1}^{K}w_{k}{\hat{d}}_{k}$$

where Φ(⋅) is the cumulative density function of a standard normal distribution and $\hat{d}(w)$ is the transfer learning discriminating vector. A simple proof of this formula is given in the Appendix. In this paper, we assume the balanced class such that $\pi_{+}=\pi_{-}$. The proof techniques generalize immediately to unbalanced cases, which is discussed briefly in Appendix C. The limiting form of Err(w) is given by Theorem 4.1 below. Two technical assumptions are required so $Z_{k}$ and the spectrum of Σ are well-behaved.

**Assumption 4 (Bounded Moment)** *Assume for each natural number* p, *the entries of* Z *written by* ${\left(Z_{k}\right)}_{ij}$ *has uniformly bounded* p*-th moment. That is, there are constants* $C_{p}$ *such that*

$$E{\left|{\left(Z_{k}\right)}_{ij}\right|}^{p}\leq C_{p}.$$

**Assumption 5 (Anisotropic Local Laws)** *Define the cumulative distribution function of* H *as* $F(x)=\sum_{i=1}^{p}I\left(\lambda_{i}\leq x\right)/p$, *where* I(⋅) *is the indicator function. Recall* $\lambda_{1}\geq \lambda_{2}\geq \cdots \geq \lambda_{p}\geq 0$ *are the eigenvalues of Σ. For a arbitrarily small positive constant* τ>0, *we assume*

$$F\left(\tau \right)\leq 1-\tau .$$

We are now in position to state the first main result of this section. The following theorem describes the asymptotic classification error for the regularized transfer learning classifier defined in (2). The result holds under the aforementioned model assumptions.

**Theorem 4.1 (Asymptotic Classification Error for TL-RDA)** *Suppose that assumptions 1–2 as well as 4 and 5 hold. Then for a fixed* K≥2, *as* $n_{k},p\to \infty ,p/n_{k}\to \gamma_{k}\in (0,\infty ]$ *for* 1≤k≤K, *we have that the limiting form of* Err(w) *for a given weight vector* $w\in R^{K}$ *is given by:*

(10) $$\Phi \left(-\frac{u^{⊤}w}{\sqrt{w^{⊤}𝒜w}}\right)$$

*where the elements of* $u\in R^{K}$ *and* $𝒜\in R^{K\times K}$ *are as follows:*

$$u_{k}=\rho_{kK}\alpha_{k}\alpha_{K}m_{F_{\gamma_{k}}}\left(-\lambda_{k}\right)\;\text{for}\;k=1,\ldots ,K,$$

*and*

$${𝒜}_{kk^{\prime }}=\left\{\begin{array}{ll} \alpha_{k}^{2}\left[\frac{v_{F_{\gamma_{k}}}\left(-\lambda_{k}\right)-\lambda_{k}v_{F_{\gamma_{k}}}^{\prime }\left(-\lambda_{k}\right)}{\gamma_{k}{\left[\lambda_{k}v_{F_{\gamma_{k}}}\left(-\lambda_{k}\right)\right]}^{2}}\right]+\frac{v_{F_{\gamma_{k}}}^{\prime }\left(-\lambda_{k}\right)-v_{F_{\gamma_{k}}}^{2}\left(-\lambda_{k}\right)}{\lambda_{k}^{2}v_{F_{\gamma_{k}}}^{4}\left(-\lambda_{k}\right)} & \text{if}\;k=k^{\prime },\\ \rho_{kk^{\prime }}\alpha_{k}\alpha_{k^{\prime }}{ℳ}_{kk^{\prime }} & \text{otherwise}, \end{array}\right.$$

*for* $k,k^{\prime }=1,\cdots ,K$. *Here* $m_{F_{\gamma_{k}}}(\cdot )$, $v_{F_{\gamma_{k}}}(\cdot )$ *and* $v_{F_{\gamma_{k}}}^{\prime }(\cdot )$ *are the Stieltjes transform, the companion Stieltjes transform and its derivative respectively, as defined in* (6)–(9). *Moreover for* $k,k^{\prime }=1,\cdots ,K$ *let* ${ℳ}_{kk^{\prime }}$ *be the constants defined as the following limiting quantities:*

$$\text{tr}\left[{\left({\hat{\Sigma }}_{k}+\lambda_{k}I_{p}\right)}^{-1}{\left({\hat{\Sigma }}_{k^{\prime }}+\lambda_{k^{\prime }}I_{p}\right)}^{-1}\Sigma \right]/p\to_{a.s.}{ℳ}_{kk^{\prime }}.$$

**Remark 4.2** *The limits of the off-diagonal terms in* 𝒜 *depends on the interplay of sample covariances in separated populations. Similar terms also appear later in the paper when finding the optimal weight vector (see Theorems 4.5, 4.10). Depending on the specific scenario, the term* ${ℳ}_{kk^{\prime }}$ *has explicit expressions, some of which are presented separately in Lemma A.8*.

**Remark 4.3** *When* K=1, *writing* $\lambda_{1},\gamma_{1},\alpha_{1},m_{F_{\gamma_{1}}}\left(-\lambda_{1}\right),v_{F_{\gamma_{1}}}\left(-\lambda_{1}\right)$, *and* $v_{F_{\gamma_{1}}}^{\prime }\left(-\lambda_{1}\right)$ *as* λ,γ,α,m,v *and* $v^{\prime }$ *respectively yields*

$${𝒜}_{11}=\frac{1}{\lambda^{2}v^{2}}\left[\frac{\alpha^{2}\left(v-\lambda v^{\prime }\right)}{\gamma }+\frac{v^{\prime }}{v^{2}}-1\right],$$

*which implies that the limiting misclassification error is given by*

$$\text{Err}(w)\to \Phi \left(\frac{\alpha^{2}m}{\sqrt{{𝒜}_{11}}}\right)=\Phi \left(-\alpha^{2}\lambda mv{\left\{\frac{\alpha^{2}\left(v-\lambda v^{\prime }\right)}{\gamma }+\frac{v^{\prime }}{v^{2}}-1\right\}}^{-1/2}\right).$$

*This matches exactly with Theorem 3.1 of* Dobriban and Wager (2018).

We now consider the classification error of the transfer learning classifier, when using the pooled covariance matrix. Let us replace $\hat{d}(w)$ with ${\hat{d}}_{P}(w)$ in Err(w) and define this new function of w as ${Err}_{P}(w)$. This gives us the classification error for TLP-RDA given a weight vector w.

**Corollary 4.4 (Asymptotic Classification Error for TLP-RDA)** *Under the same set up as Theorem 4.1, we further define* $p/\sum_{k=1}^{K}n_{k}\to \overset{‾}{\gamma }$. *Then assuming* $\lambda_{1}=\cdots =\lambda_{K}=\lambda$, *we have*

$${Err}_{P}(w)=\Phi \left(-\frac{u_{P}^{⊤}w}{\sqrt{w^{⊤}{𝒜}_{P}w}}\right)$$

*where the elements of* $u_{P}\in R^{K}$ *and* ${𝒜}_{P}\in R^{K\times K}$ *are as follows:*

$${\left(u_{P}\right)}_{k}=\rho_{kK}\alpha_{k}\alpha_{K}m_{F_{\overset{‾}{\gamma }}}\left(-\lambda \right)\;\text{for}\;k=1,\ldots ,K,$$

*and*

$${\left({𝒜}_{P}\right)}_{kk^{\prime }}=\left\{\begin{array}{ll} \alpha_{k}^{2}\left[\frac{v_{F_{\overset{‾}{\gamma }}}(-\lambda )-\lambda v_{F_{\overset{‾}{\gamma }}}^{\prime }(-\lambda )}{\overset{‾}{\gamma }{\left[\lambda v_{F_{\overset{‾}{\gamma }}}(-\lambda )\right]}^{2}}\right]+\gamma_{k}\left[\frac{v_{F_{\overset{‾}{\gamma }}}^{\prime }\left(-\lambda_{k}\right)-v_{F_{\overset{‾}{\gamma }}}^{2}(-\lambda )}{\overset{‾}{\gamma }\lambda^{2}v_{F_{\overset{‾}{\gamma }}}^{4}(-\lambda )}\right] & \text{if}\;k=k^{\prime },\\ \rho_{kk^{\prime }}\alpha_{k}\alpha_{k^{\prime }}\left[\frac{v_{F_{\overset{‾}{\gamma }}}(-\lambda )-\lambda v_{F_{\overset{‾}{\gamma }}}^{\prime }(-\lambda )}{\overset{‾}{\gamma }{\left[\lambda v_{F_{\overset{‾}{\gamma }}}(-\lambda )\right]}^{2}}\right] & \text{otherwise}, \end{array}\right.$$

*for* $k,k^{\prime }=1,\cdots ,K$. *Here* $m_{F_{\overset{‾}{\gamma }}}(\cdot ),v_{F_{\overset{‾}{\gamma }}}(\cdot )$ *and* $v_{F_{\overset{‾}{\gamma }}}^{\prime }(\cdot )$ *are the Stieltjes transform, the companion Stieltjes transform and its derivative respectively, as defined in* (6)–(9).

In this corollary, we make the assumption that all studies have the same degree of penalization, which brings us the simplified cross terms in ${𝒜}_{P}$ and a more amenable expression for ${𝒜}_{P}$ as a whole. This assumption might not be reasonable when the signal strengths $\alpha_{k}^{2}$ are vastly different, as lighter penalization may be given to population with stronger signal strength. In this case, one can use the general formula in Theorem 4.1.

### 4.2 Minimum Estimation Risk Weight

We firstly present the way to minimize the coordinate-wise estimation error of $\hat{d}(W)$ with respect to $d_{\text{Bayes}}$.

**Theorem 4.5 (Asymptotic Estimation Error Minimization for TL-RDA)** *Suppose that assumptions 1–2 as well as 4 and 5 hold. Then for a fixed* K≥2, *as* $n_{k},p\to \infty ,p/n_{k}\to \gamma_{k}\in (0,\infty ]$ *for* 1≤k≤K, *the weight for minimizing the error in estimating the Bayes optimal discriminator* $d_{\text{Bayes}}$ *is given by:*

$$w^{E}≔\text{arg}\;\underset{w}{\text{min}}{\left\|d_{\text{Bayes}}-\sum_{k=1}^{K}w_{k}{\hat{d}}_{k}\right\|}_{2}^{2}={\left({𝒜}^{E}+{ℛ}^{E}\right)}^{-1}u^{E},$$

*where the elements of* $u^{E}\in R^{K},{𝒜}^{E}\in R^{K\times K}$, *and* ${ℛ}^{E}\in R^{K\times K}$ *are:*

$${\left(u^{E}\right)}_{k}=\rho_{kK}\alpha_{k}\alpha_{K}\left[\frac{1}{\lambda_{k}}E\left(T^{-1}\right)-m_{F_{\gamma_{k}}}{\left(-\lambda_{k}\right)}^{2}\right]\;\text{for}\;k=1,\ldots ,K,$$

$${𝒜}_{kk^{\prime }}^{E}=\left\{\begin{array}{ll} \alpha_{k}^{2}m_{F_{\gamma_{k}}}^{\prime }\left(-\lambda_{k}\right) & \text{if}\;k=k^{\prime },\\ \rho_{kk^{\prime }}\alpha_{k}\alpha_{k^{\prime }}{ℰ}_{kk^{\prime }} & \text{otherwise}, \end{array}\right.$$

*and*

$${ℛ}_{kk^{\prime }}^{E}=\left\{\begin{array}{ll} \frac{v_{F_{\gamma_{k}}}\left(-\lambda_{k}\right)-\lambda_{k}v_{F_{\gamma_{k}}}^{\prime }\left(-\lambda_{k}\right)}{\lambda_{k}v_{F_{\gamma_{k}}}{\left(-\lambda_{k}\right)}^{2}} & \text{if}\;k=k^{\prime },\\ 0 & \text{otherwise}, \end{array}\right.$$

*for* $k,k^{\prime }=1,\cdots ,K$. *Here* $m_{F_{\gamma_{k}}}(\cdot )$, $v_{F_{\gamma_{k}}}(\cdot )$ *and* $v_{F_{\gamma_{k}}}^{\prime }(\cdot )$ *are the Stieltjes transform, the companion Stieltjes transform and its derivative respectively, as defined in* (6)–(9), while T *is the limiting spectral distribution of the population covariance matrix* Σ. *Moreover for* $k,k^{\prime }=1,\cdots ,K$, *let* ${ℰ}_{kk^{\prime }}$ *be the constants defined as the following limiting quantities:*

$$\text{tr}\left[{\left({\hat{\Sigma }}_{k}+\lambda_{k}I_{p}\right)}^{-1}{\left({\hat{\Sigma }}_{k^{\prime }}+\lambda_{k^{\prime }}I_{p}\right)}^{-1}\right]/p\to_{a.s.}{ℰ}_{kk^{\prime }}.$$

Just like the expressions for the asymptotic classification rate in Theorem 4.1, more explicit forms of ${ℰ}_{kk^{\prime }}$ are available if $\gamma_{k}$ and $\lambda_{k}$ are the same across populations. We call $w^{E}$ the minimum estimation weight, as it minimizes the $\ell_{2}$ error in estimating the Bayes optimal discriminating vector $d_{\text{Bayes}}$. In order to better illustrate the effect of transfer learning, we consider next a simpler situation where all the sources are homogeneous, in that $\gamma_{1}=\gamma_{2}=\cdots =\gamma_{K}$ and the corresponding regularization parameters are also the same, i.e., $\lambda_{1}=\lambda_{2}=\cdots =\lambda_{K}$. We further assume equal correlation among all sources, i.e., $\rho_{kk^{\prime }}=\rho$ whenever $k\neq k^{\prime }$.

**Corollary 4.6 (Estimation Error for Homogeneous Sources)** *Under the setup of Theorem 4.5, suppose* $\gamma_{1}=\gamma_{2}=\cdots =\gamma_{K}≕\gamma ,\lambda_{1}=\lambda_{2}=\cdots =\lambda_{K}≕\lambda$, *and* $\rho_{kk^{\prime }}=\rho$ *whenever* $k\neq k^{\prime },1\leq k,k^{\prime }\leq K$. *Then the weight vector* $w^{E}$ *that minimizes the asymptotic error in estimating* $d^{\text{Bayes}}$ *is given by:*

$$w^{E}=\alpha_{K}\left[\frac{1}{\lambda }E\left(T^{-1}\right)-m_{F_{\gamma }}(-\lambda {)}^{2}\right]\;\text{vec}\left(\frac{\{\rho -\xi +(1-\rho )I(k=K)\}\alpha_{k}}{t_{m,\varepsilon ,\lambda }\alpha_{k}^{2}+t_{v,\lambda }}:1\leq k\leq K\right)$$

*where* $\varepsilon ={ℰ}_{12},t_{\rho ,\varepsilon ,\lambda }≔-\rho \varepsilon +m_{F_{\gamma }}^{\prime }(-\lambda ),t_{v,\lambda }≔\frac{v_{F_{\gamma }}(-\lambda )-\lambda v_{F_{\gamma }}^{\prime }(-\lambda )}{\lambda v_{F_{\gamma }}(-\lambda {)}^{2}}$ *and*

$$\xi =\frac{\rho \varepsilon \sum_{k=1}^{K}\{\rho +(1-\rho )I(k=K){\}}_{\frac{\alpha_{k}}{t_{m,\varepsilon ,\lambda }\alpha_{k}^{2}+t_{v,\lambda }}}}{1+\rho \varepsilon \sum_{k=1}^{K}\frac{\alpha_{k}^{2}}{t_{m,\varepsilon ,\lambda }\alpha_{k}^{2}+t_{v,\lambda }}}.$$

The above corollary shows that when all sources have equal sample sizes, and equal correlation, the optimal weight is proportional to $\alpha_{k}\alpha_{K}/\left(t_{m,\varepsilon ,\lambda }\alpha_{k}^{2}+t_{v,\lambda }\right)$. In particular, if $\alpha_{k}\to 0$ for some source population, then the corresponding weight for that source increases at the rate of $1/\alpha_{k}$. This is intuitive since in the homogeneous setting of equal sample sizes, the signal strength is completely determined by the inverse of the variance of $\delta_{k}$, which is precisely $1/\alpha_{k}$. For practical usage of the optimal transfer weights, we now turn to the estimation of the above quantities.

**Remark 4.7 (Estimating optimal weights)** *The Marchenko-Pastur law, along with the* equations (6), (7), (8), *and* (9) *imply that the sample Stieltjes transform* $\text{tr}\left[{\left({\hat{\Sigma }}_{k}-zI_{p}\right)}^{-1}\right]$ *can be suitably used to estimate the functions* $m_{F_{\gamma_{k}}}(\cdot )$, $v_{F_{\gamma_{k}}}(\cdot )$, *and* $m_{F_{\gamma_{k}}}^{\prime }(\cdot )$ *reliably. We remind the reader that the estimation of* $\alpha_{k}^{2}$ *and* $\rho_{kk^{\prime }}$ *is tackled in* Appendix B.3*. Finally, we note that the expression of* $u^{E}$ *in all the above cases involves the expectation of* $T^{-1}$, *and we discuss a consistent estimator for* $E\left(T^{-1}\right)$ *in the following proposition, the proof of which is immediate from Corollary 4.2 of* Dobriban and Sheng (2021).

The following proposition holds under Assumptions 3.

**Proposition 4.8** *When* $\gamma_{k}<1$, *we have* $\text{tr}\left({\hat{\Sigma }}_{k}^{-1}\right)/p\to_{\text{a.s.}}E\left(T^{-1}\right)/\left(1-\gamma_{k}\right)$.

Since in our developments so far we have assumed all populations to share the same covariance matrix Σ, one can always use the pooled sample covariance in the estimation scheme above. That means we can reliably estimate $E\left(T^{-1}\right)$ as long as $\overset{‾}{\gamma }<1$. We then present the optimal estimation for TLP-RDA, i.e., the transfer learning discriminant analysis done using the pooled covariance matrix.

**Corollary 4.9 (Asymptotic Estimation Error Minimization for TLP-RDA)** *Under the same set up as Theorem 4.5, assuming* $\lambda_{1}=\cdots =\lambda_{K}=\lambda$, *we have*

$$w_{P}^{E}≔\text{arg}\;\underset{w}{\text{min}}{\left\|d_{\text{Bayes}}-\sum_{k=1}^{K}w_{k}{\overset{ˆ}{d}}_{k}^{P}\right\|}_{2}^{2}={\left({𝒜}_{P}^{E}+{ℛ}_{P}^{E}\right)}^{-1}u_{P}^{E},$$

*where the elements of* $u_{P}^{E}\in R^{K},{𝒜}_{P}^{E}\in R^{K\times K}$, *and* ${ℛ}_{P}^{E}\in R^{K\times K}$ *are:*

$${\left(u_{P}^{E}\right)}_{k}=\rho_{kK}\alpha_{k}\alpha_{K}\left[\frac{1}{\lambda }E\left(T^{-1}\right)-m_{F_{\overset{‾}{\gamma }}}(-\lambda {)}^{2}\right]\;\text{for}\;k=1,\ldots ,K,$$

*while*

$${\left({𝒜}_{P}^{E}\right)}_{kk^{\prime }}=\rho_{kk^{\prime }}\alpha_{k}\alpha_{k^{\prime }}m_{F_{\overset{‾}{\gamma }}}^{\prime }(-\lambda )$$

*and*

$${\left({ℛ}_{P}^{E}\right)}_{kk^{\prime }}=\left\{\begin{array}{ll} \gamma_{k}\frac{v_{F_{\overset{‾}{\gamma }}}(-\lambda )-\lambda v_{F_{\overset{‾}{\gamma }}}^{\prime }(-\lambda )}{\overset{‾}{\gamma }\lambda v_{F_{\overset{‾}{\gamma }}}(-\lambda {)}^{2}} & \text{if}\;k=k^{\prime },\\ 0 & \text{otherwise}, \end{array}\right.$$

*for* $k,k^{\prime }=1,\cdots ,K$.

Once again we can follow the remark and proposition above for estimating the weight that minimizes the estimation error when using the pooled covariance matrix.

### 4.3 Minimum Prediction Risk Weight

In the high dimensional regime considered in this paper, a weight minimizing the estimation error does not translate into maximizing the classification score $\hat{d}(w{)}^{⊤}x_{0}$. We can as well compute the optimal prediction weight by directly minimizing the difference between the TL-RDA classification score and the Bayes classification score. This is precisely the objective of the next theorem.

**Theorem 4.10 (Asymptotic Prediction Error Minimization for TL-RDA)** *Suppose that Assumptions 1–2 as well as 4 and 5 hold. Then for a fixed* K≥2, *as* $n_{k},p\to \infty ,p/n_{k}\to \gamma_{k}\in (0,\infty ]$ *for* 1≤k≤K, *the weight for minimizing the excess risk, i.e., the error in predicting the class at a random test point* $x_{0}$, *when compared to the Bayes optimal discriminator* $d_{\text{Bayes}}$*, is given by:*

$$w^{P}≔\text{arg}\;\underset{w}{\text{min}}\;E_{x_{0}}{\left[{\left(d_{\text{Bayes}}-\sum_{k=1}^{K}w_{k}{\hat{d}}_{k}\right)}^{⊤}x_{0}\right]}^{2}={\left({𝒜}^{P}+{ℛ}^{P}\right)}^{-1}u^{P},$$

*where the elements of* $u^{P}\in R^{K},{𝒜}^{P}\in R^{K\times K}$, *and* ${ℛ}^{P}\in R^{K\times K}$ *are:*

$${\left(u^{P}\right)}_{k}=\rho_{kK}\alpha_{k}\alpha_{K}m_{F_{\gamma_{k}}}\left(-\lambda_{k}\right)\;\text{for}\;k=1,\ldots ,K,$$

$${𝒜}_{kk^{\prime }}^{P}=\left\{\begin{array}{ll} \alpha_{k}^{2}\left[\frac{v_{F_{\gamma_{k}}}\left(-\lambda_{k}\right)-\lambda_{k}v_{F_{\gamma_{k}}}^{\prime }\left(-\lambda_{k}\right)}{\gamma_{k}{\left[\lambda_{k}v_{F_{\gamma_{k}}}\left(-\lambda_{k}\right)\right]}^{2}}\right] & \text{if}\;k=k^{\prime },\\ \rho_{kk^{\prime }}\alpha_{k}\alpha_{k^{\prime }}{ℳ}_{kk^{\prime }} & \text{otherwise}, \end{array}\right.$$

*and*

$${ℛ}_{kk^{\prime }}^{E}=\left\{\begin{array}{ll} \frac{v_{k}^{\prime }\left(-\lambda_{k}\right)-v_{k}^{2}\left(-\lambda_{k}\right)}{\lambda_{k}^{2}v_{k}^{4}\left(-\lambda_{k}\right)} & \text{if}\;k=k^{\prime },\\ 0 & \text{otherwise}, \end{array}\right.$$

*for* $k,k^{\prime }=1,\cdots ,K$. *Here* $m_{F_{\gamma_{k}}}(\cdot ),v_{F_{\gamma_{k}}}(\cdot )$ *and* $v_{F_{\gamma_{k}}}^{\prime }(\cdot )$ *are the Stieltjes transform, the companion Stieltjes transform and its derivative respectively, as defined in* (6)–(9). *Moreover for* $k,k^{\prime }=1,\cdots ,K$, *let* ${ℳ}_{kk^{\prime }}$ *be the constants defined as the following limiting quantities:*

$$\text{tr}\left[{\left({\hat{\Sigma }}_{k}+\lambda_{k}I_{p}\right)}^{-1}{\left({\hat{\Sigma }}_{k^{\prime }}+\lambda_{k^{\prime }}I_{p}\right)}^{-1}\Sigma \right]/p\to_{a.s.}{ℳ}_{kk^{\prime }}.$$

As in the case of estimation error weights, we discuss the issue of estimating the optimal weight that minimizes prediction error in this setting. Note that unlike the estimation error minimizing weight ${𝒲}^{E}$, the prediction risk minimizing weight ${𝒲}^{P}$, involves no population spectral distribution. It is thus estimable in all cases, including the case when γ>1. On the other hand, estimating ${ℳ}_{kk^{\prime }}$ requires more care, especially in the case k=K or $k^{\prime }=K$. The following proposition describes the estimation in this case.

**Proposition 4.11** *We have the following consistent estimators* ${\hat{ℳ}}_{kk^{\prime }}$ *for* ${ℳ}_{kk^{\prime }}$ *separately for three cases:*

$${\hat{ℳ}}_{kk^{\prime }}=\left\{\begin{array}{ll} \text{tr}\left[{\left({\hat{\Sigma }}_{k}+\lambda_{k}I_{p}\right)}^{-1}{\left({\hat{\Sigma }}_{k^{\prime }}+\lambda_{k^{\prime }}I_{p}\right)}^{-1}{\hat{\Sigma }}_{K}\right]/p & \text{if}\;k\neq k^{\prime },k\neq K,k^{\prime }\neq K\\ \text{tr}\left[{\left({\hat{\Sigma }}_{k}+\lambda_{k}I_{p}\right)}^{-2}{\hat{\Sigma }}_{K}\right]/p & \text{if}\;k=k^{\prime }\neq K\\ \frac{1}{px_{p}}\text{tr}\left[{\left({\hat{\Sigma }}_{k^{\prime }}+\lambda_{k^{\prime }}I_{p}\right)}^{-1}\right]-\frac{\lambda_{K}}{px_{p}}\text{tr}\left[{\left({\hat{\Sigma }}_{K}+\lambda_{K}I_{p}\right)}^{-1}{\left({\hat{\Sigma }}_{k^{\prime }}+\lambda_{k^{\prime }}I_{p}\right)}^{-1}\right] & \text{if}\;k=K,k^{\prime }\neq K \end{array}\right.$$

*where* $x_{p}=x\left(\gamma_{K},\lambda_{K}\right)$ *is the solution to the equation:*

$$1-x_{p}=\gamma_{K}\left[1-\lambda_{K}\int {\left(x_{p}t+\lambda_{K}\right)}^{-1}dH_{K}(t)\right]$$

*Then*

$${ℳ}_{kk^{\prime }}-{\hat{ℳ}}_{kk^{\prime }}\to_{a.s.}0.$$

In order to better illustrate the effect of transfer learning, we consider next a simpler situation where all the sources are homogeneous, in that $\gamma_{1}=\gamma_{2}=\cdots =\gamma_{K}$ and the corresponding regularization parameters are also the same, i.e., $\lambda_{1}=\lambda_{2}=\cdots =\lambda_{K}$. We further assume equal correlation among all sources, i.e., $\rho_{kk^{\prime }}=\rho$ whenever $k\neq k^{\prime }$.

**Corollary 4.12 (Prediction Error for Homogeneous Sources)** *Under the earlier setup of Theorem 4.5, suppose* $\gamma_{1}=\gamma_{2}=\cdots =\gamma_{K}≕\gamma ,\lambda_{1}=\lambda_{2}=\cdots =\lambda_{K}≕\lambda$, *and* $\rho_{kk^{\prime }}=\rho$ *whenever* $k\neq k^{\prime },1\leq k,k^{\prime }\leq K$. *Then the weight vector* $w^{P}$ *that minimizes the asymptotic excess risk is given by:*

$$w^{P}=\alpha_{K}m_{F_{\gamma }}(-\lambda )\text{vec}\left(\frac{\left\{\rho -\xi^{P}+(1-\rho )I(k=K)\right\}\alpha_{k}}{t_{v,\rho ,\lambda }^{P}\alpha_{k}^{2}+t_{v,\lambda }^{P}}:1\leq k\leq K\right)$$

*where* $m={ℳ}_{12},t_{v,\rho ,\lambda }^{P}≔-m\rho +\frac{v_{F_{\gamma }}(-\lambda )-\lambda v_{F_{\gamma }}^{\prime }(-\lambda )}{\gamma {\left[\lambda v_{F_{\gamma }}(-\lambda )\right]}^{2}},t_{v,\lambda }^{P}≔\frac{v^{\prime }(-\lambda )-v^{2}(-\lambda )}{\lambda^{2}v^{4}(-\lambda )}$ *and*

$$\xi^{P}=\frac{m\rho \sum_{k=1}^{K}\{\rho +(1-\rho )I(k=K)\}\frac{\alpha_{k}^{2}}{t_{v,\rho ,\lambda }^{P}\alpha_{k}^{2}+t_{v,\lambda }^{P}}}{1+m\rho \sum_{k=1}^{K}\frac{\alpha_{k}^{2}}{t_{v,\rho ,\lambda }^{P}\alpha_{k}^{2}+t_{v,\lambda }^{P}}}.$$

The above corollary shows that when all sources have equal sample sizes, and equal correlation, the optimal weight is proportional to $\alpha_{k}\alpha_{K}/\left(t_{v,\rho ,\lambda }^{P}\alpha_{k}^{2}+t_{v,\lambda }^{P}\right)$. Thus similar to Corollary 4.6, if $\alpha_{k}\to 0$ for some source population, then the corresponding weight for that source increases at the rate of $1/\alpha_{k}$. This is intuitive since in the homogeneous setting of equal sample sizes, the signal strength is completely determined by the inverse of the variance of $\delta_{k}$, which is precisely $1/\alpha_{k}$. Finally, since our assumption so far posits the same variance Σ for all populations, we also describe the estimation based on the pooled sample covariance matrix. This is given in the following corollary for the pooled covariance based classifier TLP-RDA.

**Corollary 4.13 (Asymptotic Prediction Error Minimization for TLP-RDA)** *Under the same set up as theorem 4.10, assume* $\lambda_{1}=\cdots =\lambda_{K}=\lambda$, *we have*

$$w_{P}^{P}≔\text{arg}\;\underset{w}{\text{min}}\;E_{x_{0}}{\left[{\left(d_{\text{Bayes}}-\sum_{k=1}^{K}w_{k}{\hat{d}}_{k}^{P}\right)}^{⊤}x_{0}\right]}^{2}={\left({𝒜}_{P}^{P}+{ℛ}_{P}^{P}\right)}^{-1}u_{P}^{P}$$

*where the elements of* $u_{P}^{P}\in R^{K},{𝒜}_{P}^{P}\in R^{K\times K}$, *and* ${ℛ}_{P}^{P}\in R^{K\times K}$ *are:*

$${\left(u_{P}^{P}\right)}_{k}=\rho_{kK}\alpha_{k}\alpha_{K}m_{F_{\overset{‾}{\gamma }}}\left(-\lambda \right)\;\text{for}\;k=1,\ldots ,K,$$

*while*

$${\left({𝒜}_{P}^{P}\right)}_{kk^{\prime }}=\rho_{kk^{\prime }}\alpha_{k}\alpha_{k^{\prime }}\left[\frac{v_{F_{\overset{‾}{\gamma }}}(-\lambda )-\lambda v_{F_{\overset{‾}{\gamma }}}^{\prime }(-\lambda )}{\overset{‾}{\gamma }{\left[\lambda v_{F_{\overset{‾}{\gamma }}}(-\lambda )\right]}^{2}}\right]$$

*and*

$${\left({ℛ}_{P}^{P}\right)}_{kk^{\prime }}=\left\{\begin{array}{ll} \gamma_{k}\frac{v_{F_{\overset{‾}{\gamma }}}^{\prime }\left(-\lambda_{k}\right)-v_{F_{\overset{‾}{\gamma }}}^{2}(-\lambda )}{\overset{‾}{\gamma }\lambda^{2}v_{F_{\overset{‾}{\gamma }}}^{4}(-\lambda )} & \text{if}\;k=k^{\prime },\\ 0 & \text{otherwise}, \end{array}\right.$$

*for* $k,k^{\prime }=1,\cdots ,K$.

The optimal prediction weights $w_{P}^{E},w_{P}^{P}$ dominate $w^{E},w^{P}$ respectively in classification error, meaning

$$Err\left(w^{E}\right)\geq Err\left(w^{P}\right),{Err}_{P}\left(w_{P}^{E}\right)\geq {Err}_{P}\left(w_{P}^{P}\right).$$

Note that a priori it is not immediate that the weight which minimizes the difference in prediction error from the Bayes optimal direction, also minimizes the classification error. In particular, the accuracy of classification depends on the sign of $\hat{d}(w{)}^{⊤}x_{0}$, and not the actual value. However, we now show that in addition to optimizing the score $\hat{d}(w{)}^{⊤}x_{0}$, the prediction weights $w^{P}$ and $w_{P}^{P}$ also minimize the misclassification error. This is formalized by the following proposition.

**Proposition 4.14** *The optimal prediction weight minimizes the testing data classification error:*

$$w^{P}=\text{arg}\;\underset{w}{\text{min}}\;E_{x_{0},y_{0}}\left[I\left(\text{sign}\left[(\hat{d}(w){)}^{⊤}x_{0}\right]\neq y_{0}\right)\right],$$

$$w_{P}^{P}=\text{arg}\;\underset{w}{\text{min}}\;E_{x_{0},y_{0}}\left[I\left(\text{sign}\left[{\left({\hat{d}}_{P}(w)\right)}^{⊤}x_{0}\right]\neq y_{0}\right)\right].$$

We conclude this section by validating the existence of the optimal weights. Indeed, the solution to all four types of weights implicitly assume that the matrices ${𝒜}^{E}+{ℛ}^{E},{𝒜}^{P}+{ℛ}^{P},{𝒜}_{P}^{E}+{ℛ}_{P}^{E},{𝒜}_{P}^{P}+{ℛ}_{P}^{P}$ are invertible. We prove this is always the case in the following proposition.

**Proposition 4.15 (Existence of Optimal Weights)** *The matrices* ${𝒜}^{E}+{ℛ}^{E},{𝒜}^{P}+{ℛ}^{P},{𝒜}_{P}^{E}+{ℛ}_{P}^{E},{𝒜}_{P}^{P}+{ℛ}_{P}^{P}$ *are invertible, and hence the limiting optimal weights* $w^{E},w^{P},w_{P}^{E},w_{P}^{P}$ *exist*.

We compare the error rates of the four transfer learning estimators under several different scenarios in Appendix B.

### 4.4 Geometric Interpretation

We now provide some geometric interpretations on the optimal weights derived in the previous subsections. Let us define the following discriminant directions:

$$d_{est}≔\hat{d}\left(w_{E}\right),\;d_{err}≔\hat{d}\left(w_{P}\right).$$

Also recall the Bayes discriminant direction $d_{\text{Bayes}}$. These discriminant directions are visualized in Figure 1. The blue plane is the space spanned by linear combinations of the local discriminant directions $\left\{{\hat{d}}_{k}\right\}$, and the top black line stands for the Bayes direction, which is not necessarily in the linear span of $\left\{{\hat{d}}_{k}\right\}$. Since both the TL-RDA directions $d_{\text{est}}$ and $d_{\text{err}}$ are linear combinations of ${\hat{d}}_{k}$, they both lie on the blue plane and are denoted by colored lines. The direction obtained by minimizing the error in estimating $d_{\text{Bayes}}$ is given by $d_{\text{est}}$, which is the projection of $d_{\text{Bayes}}$ onto the blue plane, denoting $\text{span}\left\{{\hat{d}}_{k}\right\}$. The criterion 3 suggests that $d_{\text{est}}$ is the minimizer of the OLS loss when fitting $d_{\text{Bayes}}$ with linear combinations of $d_{k}$.

Denoting the angle between $d_{\text{Bayes}}$ and $\hat{d}(w)$ as θ, we now show that the cosine of θ is directly related to Err(w) accounting for the scaling Σ. Let us define the scaled inner product $\langle a,b\rangle_{\Sigma }=a^{⊤}\Sigma b$ and the scaled cos angle ${\text{cos}}_{\Sigma }(a,b)=\langle a,b\rangle_{\Sigma }/\sqrt{\langle a,a\rangle_{\Sigma }\langle b,b\rangle_{\Sigma }}$. Then we have

$$\text{cos}\;\theta ≔{\text{cos}}_{\Sigma }∠\left(\hat{d}(w),d_{\text{Bayes}}\right)=\hat{d}(w{)}^{⊤}\delta_{K}/\sqrt{\hat{d}(w{)}^{⊤}\Sigma \hat{d}(w)\delta_{K}^{⊤}\Sigma^{-1}\delta_{K}}=\frac{\Theta (w)}{\Theta_{\text{Bayes}}}$$

$$\Theta (w)≔\frac{\hat{d}(w{)}^{⊤}\delta_{K}}{\sqrt{\hat{d}(w{)}^{⊤}\Sigma \hat{d}(w)}},\;\Theta_{\text{Bayes}}≔\sqrt{\delta_{K}^{⊤}\Sigma^{-1}\delta_{K}}$$

Recall that Φ(-Θ(w)) is the classification error rate of TL-RDA (Theorem 4.1) and $\Phi \left(-\Theta_{\text{Bayes}}\right)$ is the Bayes error rate. This implies that

$$\text{cos}\;\theta =\frac{\Theta (w)}{\Theta_{\text{Bayes}}}=\frac{\Phi^{-1}(Err(w))}{\Phi^{-1}\left({Err}_{\text{Bayes}}\right)}.$$

Since $\Phi^{-1}(\cdot )$ is a monotonically increasing function, it is clear that cos(θ) is close to one, i.e., θ is close to zero, if and only if Err(w) is close to the Bayes error ${Err}_{\text{Bayes}}$. Therefore, the size of θ directly quantifies the inefficiency of TL-RDA relative to $d_{\text{Bayes}}$ in terms of classification error, and $w^{P}$ simultaneously minimizes the classification error and cosθ (Proposition 4.14).

Finally, let us also consider an observation $x_{0}$ pointing in a random direction, and denote the angle between $d_{\text{err}}$ and $x_{0}$ as β and the angle between $d_{\text{Bayes}}$ and $x_{0}$ as α. Straightforwardly, $d_{\text{err}}$ minimizes the difference between β and α as $w^{P}$ minimizes the difference between the inner products $d_{\text{err}}^{⊤}x_{0}$ and $d_{\text{Bayes}}^{⊤}x_{0}$.

## 5 Robustness and Weight Selection

In this section, we present some guidance on how one can choose between different weighting schemes. As we have repeatedly illustrated in the previous section, the $d_{\text{est}}$ is dominated by $d_{\text{err}}$ in terms of classification error on unseen data. This claim, however, holds only when the test data distribution is as given by Assumption 1. The first goal of this section is to demonstrate that $d_{\text{est}}$ outperforms $d_{\text{err}}$ considerably in terms of classification error, when there is a distribution shift in the covariates. The second goal is to compare between the individual vs. pooled covariance estimators: i.e., TL-RDA and TLP-RDA. We will prove, at least in special cases, that the optimal TLP-RDA outperforms the optimal TL-RDA when the aspect ratio is large enough.

### 5.1 Robustness of Optimal Estimation Weight

In this section we demonstrate the robustness of $d_{\text{est}}$ to covariate shifts in test data. This robustness is intuitive as we know the optimal estimation weight only attempts to minimize the difference in TL discriminant direction $\left(\hat{d}(w)\right)$ and the Bayes discriminant direction $d_{\text{Bayes}}$. In fact, we can show the weights obtained by minimizing criteria (3) are equivalent to a conservative solution to the problem of minimizing criteria (4) when test data distribution is unknown. A similar argument is in Zhang and Li (2023), we summarize it in the following proposition.

**Proposition 5.1 (Robustness of Estimation Weight)** *For the class of covariate distributions given by* $𝒫≔\left\{P:x\sim P,E_{P}\left(\|x{\|}_{2}\right)\leq c\right\}$, *we have:*

$$\underset{w}{\text{arg}\;\text{min}}{\left\|d_{\text{Bayes}}-\sum_{k=1}^{K}w_{k}{\hat{d}}_{k}\right\|}^{2}=\underset{w}{\text{arg}\;\text{min}}\underset{x_{0}\in 𝒫}{\text{max}}\;E_{x_{0}}{\left[{\left(d_{\text{Bayes}}-\sum_{k=1}^{K}w_{k}{\hat{d}}_{k}\right)}^{⊤}x_{0}\right]}^{2}.$$

Proposition 5.1 claims that, when we only know the target data comes from a distribution with bounded expected norm, the safest option to minimize prediction error criteria (4) is to focus only on the estimation error criteria (3). We demonstrate the usefulness of this in Figure 2. Here p=150 and $n_{k}=250,\cdots ,160$ for study 1 to study K. The pairwise correlations across studies are fixed at 0.5. We use the same Toeplitz covariance matrix for all training data, however, the eigenvalues of the test data $x_{0}$ covariance matrix are modified to decay much faster. The testing accuracy of the three methods are shown on the y axis as λ changes. One can see the optimal estimation weight consistently outperforms others, suggesting the robustness and conservative nature of the optimal estimation weight boost the performance of TL-RDA when there is a change in testing data distribution.

### 5.2 Pooled Sample Covariance and Individual Sample Covariance Matrix

For TLP-RDA, all discriminant directions uses the same covariance estimate ${\hat{\Sigma }}_{P}$. Although ${\hat{\Sigma }}_{P}$ is a better covariance estimate than all ${\hat{\Sigma }}_{k},{\hat{d}}_{k}^{P}$ are inevitably similar and the columns space of ${\hat{d}}_{k}^{P}$ would be less informational. This essentially becomes a bias-variance trade off. When $\gamma_{k}$ is small, the estimates ${\hat{d}}_{k}$ are reliable already. In this case, individual covariance matrices bring more variances to this column space, therefore, increase the quality of final TL-RDA estimator. When $\gamma_{k}$ are large, direction estimate based on ${\hat{\Sigma }}_{k}$ are no longer reliable and one should consider the more stable ${\hat{\Sigma }}_{P}$. We will formalize this statement in this section also.

**Proposition 5.2 (TLP-RDA out performs TL-RDA when γ is large)** *Assume* $\Sigma =I_{p},\gamma_{1}=\cdots =\gamma_{K}=\gamma ,\lambda_{1},\cdots ,\lambda_{K}=\lambda =r\left(\gamma -\frac{1}{r+1}\right)$ *for some fixed* $r>(1-\gamma {)}_{+}/\gamma$. *For the pooled covariance matrix, we choose* $\lambda^{\prime }=r^{\prime }\left(\gamma /K-\frac{1}{r^{\prime }+1}\right)$ *for some* $r^{\prime }>(K-\gamma {)}_{+}/\gamma$.

1. *When* $\rho =1,Err\left(w^{P}\right)\geq {Err}_{P}\left(w_{P}^{P}\right)$, *if and only if*
$$\gamma^{2}\left[{\left(1+r^{\prime }\right)}^{2}-K(1+r{)}^{2}\right]\geq K\left(\left[\gamma (1+r{)}^{2}-1\right]\sum_{k}\alpha_{k}^{2}\right).$$
2. *When* $\rho =0,Err\left(w^{P}\right)\geq {Err}_{P}\left(w_{P}^{P}\right)$, *if and only if*
$$\gamma \left[{\left(1+r^{\prime }\right)}^{2}-(1+r{)}^{2}\right]\geq K-1.$$

We also numerically demonstrate this phenomenon by plugging values into the limiting expressions under a more general set up (Figure 3). We again use ρ=0.5 while changing the other parameters, including the number of auxiliary studies K, the decay rate of the eigenvalues of Σ and the signal strength α. We can see that TLP-RDA can outperform TL-RDA in all cases as $\gamma_{K}$ grows, as the error rates of TLP-RDA decrease at slower rates. The transition point $\gamma^{*}$, defined as the $\gamma_{K}$ when TLP-RDA outperforms TL-RDA, differs in different scenarios. One can see that it decreases when the eigenvalues of Σ decrease slower, and interestingly, also when the number of auxiliary population decreases.

## 6 Heterogeneous Population Covariance Matrix

The previous discussions of TL-RDA have been restricted to the case where observed covariates $X_{k}$ in all populations k=1,⋯,K share the same covariance matrix Σ. In this section we extend TL-RDA to accommodate the scenario when covariance matrices are different. We call this generalization beyond identical matrices as transfer-learning-heterogeneous (TLH)-RDA. We firstly formalize this set up.

**Assumption 6 (Heterogeneous Two-class Gaussian)** *We assume all populations* k=1,⋯,K *follow the classic two-class Gaussian mixture model. More specifically, for* $i=1,\ldots ,n_{k}$ *and* k=1,…,K,

(11) $${\left(y_{k}\right)}_{i}\in \{-1,+1\}\;P\left({\left(y_{k}\right)}_{i}=\pm 1\right)=\pi_{\pm 1}\;{\left(X_{k}\right)}_{i}|{\left(y_{k}\right)}_{i}\sim N\left(\mu_{{\left(y_{k}\right)}_{i}},\Sigma_{k}\right)$$

Note that this is identical to the original set up except each population has a different population covariance matrix $\Sigma_{k}$. In addition, we assume the assumption 3 holds for all covariance matrices $\Sigma_{k}$.

**Assumption 7 (Heterogeneous rmt assumption)** *For* k=1,⋯,K, *the design matrix* $X_{k}\in R^{n_{k}\times p}$ *is generated as*

$$X_{k}={\left(\mu_{{\left(y_{k}\right)}_{1},k}\mu_{{\left(y_{k}\right)}_{2},k}\ldots \mu_{{\left(y_{k}\right)}_{n_{k}},k}\right)}^{⊤}+Z_{k}\Sigma_{k}^{1/2}$$

*for a matrix* $Z_{k}\in R^{n_{k}\times p}$ *with i.i.d. entries coming from an infinite array. The entries* ${\left(Z_{k}\right)}_{ij}$ *of* $Z_{k}$ *satisfy the moment conditions:*

$$E\left[{\left(Z_{k}\right)}_{ij}\right]=0,\;E\left[{\left(Z_{k}\right)}_{ij}^{2}\right]=1\;\text{and}\;E\left[{\left(Z_{k}\right)}_{ij}^{4}\right]\leq C.$$

1. *The population covariance matrix* $\Sigma_{k}\in R^{p\times p}$ *is deterministic. The observations have unit variance, i.e*., ${\left(\Sigma_{k}\right)}_{jj}=1$ *for* j=1,⋯,p.
2. *The eigenvalues of* $\Sigma_{k}$ *are uniformly bounded from above and away from zero with constants independent of the dimension* p.
3. *The sequence of spectral distributions* $T_{k}≔{\left(T_{k}\right)}_{\Sigma ,p}$ *of* $\Sigma_{k}≔{\left(\Sigma_{k}\right)}_{p}$ *converges weakly to a limiting distribution* $H_{k}$ *supported on* [0,∞), *called the population spectral distribution* (psd).

We can then derive the optimal prediction and estimation weights in a manner identical to Theorems 4.5 and 4.10. Since the Bayes optimal discriminant direction is given by $\Sigma_{K}^{-1}\delta_{K}$, and as before we aim to leverage the related observations in each source through a weighted linear combination of their discriminant directions ${\hat{d}}_{k}$, for k=1,…,K.

**Theorem 6.1 (Asymptotic Estimation Error Minimization for TLH-RDA)** *Suppose that assumptions 1, 2, 4, 5 as well as 6 and 7 hold. Then for a fixed* K≥2, *as* $n_{k},p\to \infty ,p/n_{k}\to \gamma_{k}\in (0,\infty ]$ *for* 1≤k≤K, *the weight for minimizing the error in estimating the Bayes optimal discriminator* $d_{\text{Bayes}}$ *is given by:*

$$w_{H}^{E}≔\text{arg}\;\underset{w}{\text{min}}{\left\|d_{\text{Bayes}}-\sum_{k=1}^{K}w_{k}{\hat{d}}_{k}\right\|}_{2}^{2}={\left({𝒜}_{H}^{E}+{ℛ}_{H}^{E}\right)}^{-1}u_{H}^{E},$$

*where the elements of* $u_{H}^{E}\in R^{K},{𝒜}_{H}^{E}\in R^{K\times K}$, *and* ${ℛ}_{H}^{E}\in R^{K\times K}$ *are:*

$${\left(u_{H}^{E}\right)}_{k}=\left\{\begin{array}{ll} \rho_{kK}\alpha_{k}\alpha_{K}\;\text{tr}\left(\Sigma_{K}^{-1}{\left({\hat{\Sigma }}_{k}+\lambda_{k}I_{p}\right)}^{-1}\right) & \text{if}\;k=1,\ldots ,K-1,\\ \alpha_{K}^{2}\left[\frac{1}{\lambda_{k}}E\left(T_{K}^{-1}\right)-m_{F_{\gamma_{k}}}{\left(-\lambda_{k}\right)}^{2}\right] & \text{if}\;k=K. \end{array}\right.$$

$${\left({𝒜}_{H}^{E}\right)}_{kk^{\prime }}=\left\{\begin{array}{ll} \alpha_{k}^{2}m_{F_{\gamma_{k}}}^{\prime }\left(-\lambda_{k}\right) & \text{if}\;k=k^{\prime },\\ \rho_{kk^{\prime }}\alpha_{k}\alpha_{k^{\prime }}{𝒰}_{kk^{\prime }} & \text{otherwise}, \end{array}\right.$$

*and*

$${\left({ℛ}_{H}^{E}\right)}_{kk^{\prime }}=\left\{\begin{array}{ll} \frac{v_{F_{\gamma_{k}}}\left(-\lambda_{k}\right)-\lambda_{k}v_{F_{\gamma_{k}}}^{\prime }\left(-\lambda_{k}\right)}{\lambda_{k}v_{F_{\gamma_{k}}}{\left(-\lambda_{k}\right)}^{2}} & \text{if}\;k=k^{\prime },\\ 0 & \text{otherwise}, \end{array}\right.$$

*for* $k,k^{\prime }=1,\cdots ,K$. *Moreover for* $k,k^{\prime }=1,\cdots ,K$, *let* ${𝒰}_{kk^{\prime }}$ *be the constants defined as the following limiting quantities:*

(12) $$\text{tr}\left[{\left({\hat{\Sigma }}_{k}+\lambda_{k}I_{p}\right)}^{-1}{\left({\hat{\Sigma }}_{k^{\prime }}+\lambda_{k^{\prime }}I_{p}\right)}^{-1}\right]/p\to_{a.s.}{𝒰}_{kk^{\prime }}.$$

Since the quantities on the left hand side of (12) are exactly known in terms of sample quantities, we do not seek the exact limits of the traces of the cross sample covariance terms ${𝒰}_{kk^{\prime }}$. Instead, we advocate directly using the known quantities

$$\text{tr}\left[{\left({\hat{\Sigma }}_{k}+\lambda_{k}I_{p}\right)}^{-1}{\left({\hat{\Sigma }}_{k^{\prime }}+\lambda_{k^{\prime }}I_{p}\right)}^{-1}\right]/p.$$

The situation changes however for estimating ${\left(u_{H}^{E}\right)}_{k}$ when k≠K, since they depend on the unknown target population covariance $\Sigma_{K}$. This is guaranteed by the almost sure convergence of a suitable sample based quantity, as recorded in the following proposition.

**Proposition 6.2**

$$\text{tr}\left(\Sigma_{K}^{-1}{\left({\hat{\Sigma }}_{k}+\lambda_{k}I_{p}\right)}^{-1}\right)-\left(1-\gamma_{K}\right)\text{tr}\left({\hat{\Sigma }}_{K}^{-1}{\left({\hat{\Sigma }}_{k}+\lambda_{k}I_{p}\right)}^{-1}\right)\to_{a.s.}0$$

*as* $n_{k},p\to 0;p/n_{k}\to \gamma_{k}$ *for* k=1,⋯,K-1.

The proof of this proposition is immediate from Corollary 4.2 of Dobriban and Sheng (2021). We next move on to the optimal prediction weights for TLH-RDA.

**Theorem 6.3 (Asymptotic Prediction Error Minimization for TLH-RDA)** *Suppose that assumptions 1, 2 as well as 6 and 7 hold. Then for a fixed* K≥2, *as* $n_{k},p\to \infty ,p/n_{k}\to \gamma_{k}\in (0,\infty ]$ *for* 1≤k≤K, *the weight for minimizing the excess risk, i.e., the error in predicting the class at a random test point* $x_{0}$, *when compared to the Bayes optimal discriminator* $d_{\text{Bayes}}$, *is given by:*

$$w_{H}^{P}≔\text{arg}\;\underset{w}{\text{min}}\;E_{x_{0}}{\left[{\left(d_{\text{Bayes}}-\sum_{k=1}^{K}w_{k}{\hat{d}}_{k}\right)}^{⊤}x_{0}\right]}^{2}={\left({𝒜}_{H}^{P}+{ℛ}_{H}^{P}\right)}^{-1}u_{H}^{P},$$

*where the elements of* $u^{P}\in R^{K},{𝒜}^{P}\in R^{K\times K}$, *and* ${ℛ}^{P}\in R^{K\times K}$ *are:*

$${\left(u_{H}^{P}\right)}_{k}=\rho_{kK}\alpha_{k}\alpha_{K}m_{F_{\gamma_{k}}}\left(-\lambda_{k}\right)\;\text{for}\;k=1,\ldots ,K,$$

$${\left({𝒜}_{H}^{P}\right)}_{kk^{\prime }}=\left\{\begin{array}{ll} \alpha_{k}^{2}\left[\frac{v_{F_{\gamma_{k}}}\left(-\lambda_{k}\right)-\lambda_{k}v_{F_{\gamma_{k}}}^{\prime }\left(-\lambda_{k}\right)}{\gamma_{k}{\left[\lambda_{k}v_{F_{\gamma_{k}}}\left(-\lambda_{k}\right)\right]}^{2}}\right] & \text{if}\;k=k^{\prime }=K,\\ \rho_{kk^{\prime }}\alpha_{k}\alpha_{k^{\prime }}{𝒴}_{kk^{\prime }} & \text{otherwise}, \end{array}\right.$$

*and*

$${\left({ℛ}_{H}^{E}\right)}_{kk^{\prime }}=\left\{\begin{array}{ll} \frac{v_{k}^{\prime }\left(-\lambda_{k}\right)-v_{k}^{2}\left(-\lambda_{k}\right)}{\lambda_{k}^{2}v_{k}^{4}\left(-\lambda_{k}\right)} & \text{if}\;k=k^{\prime },\\ 0 & \text{otherwise}, \end{array}\right.$$

*for* $k,k^{\prime }=1,\cdots ,K$. *Moreover for* $k,k^{\prime }=1,\cdots ,K$, *let* ${𝒴}_{kk^{\prime }}$ *be the constants defined as the following limiting quantities:*

$$\text{tr}\left[{\left({\hat{\Sigma }}_{k}+\lambda_{k}I_{p}\right)}^{-1}{\left({\hat{\Sigma }}_{k^{\prime }}+\lambda_{k^{\prime }}I_{p}\right)}^{-1}\Sigma_{K}\right]/p\to_{a.s.}{𝒴}_{kk^{\prime }}.$$

In order to utilize the above weights in a practical scenario, we need to replace all unknown quantities by their estimates. As before, the Marcenko Pastur law is crucial in estimating the parameters related to the spectral distributions of $\Sigma_{k}$, for k=1,…,K. Most of the details are straightforward and identical in estimation procedure as the rest of the paper. It remains to provide consistent estimators for ${𝒴}_{kk^{\prime }}$, which we describe in the following proposition.

**Proposition 6.4** *We have the following consistent estimators* ${\hat{𝒴}}_{kk^{\prime }}$ *for* ${𝒴}_{kk^{\prime }}$ *separately for three cases:*

$${\hat{𝒴}}_{kk^{\prime }}=\left\{\begin{array}{ll} \text{tr}\left[{\left({\hat{\Sigma }}_{k}+\lambda_{k}I_{p}\right)}^{-1}{\left({\hat{\Sigma }}_{k^{\prime }}+\lambda_{k^{\prime }}I_{p}\right)}^{-1}{\hat{\Sigma }}_{K}\right]/p & \text{if}\;k\neq k^{\prime },k\neq K,k^{\prime }\neq K\\ \text{tr}\left[{\left({\hat{\Sigma }}_{k}+\lambda_{k}I_{p}\right)}^{-2}{\hat{\Sigma }}_{K}\right]/p & \text{if}\;k=k^{\prime }\neq K\\ \frac{1}{px_{p}}\text{tr}\left[{\left({\hat{\Sigma }}_{k^{\prime }}+\lambda_{k^{\prime }}I_{p}\right)}^{-1}\right]-\frac{\lambda_{K}}{px_{p}}\text{tr}\left[{\left({\hat{\Sigma }}_{K}+\lambda_{K}I_{p}\right)}^{-1}{\left({\hat{\Sigma }}_{k^{\prime }}+\lambda_{k^{\prime }}I_{p}\right)}^{-1}\right] & \text{if}\;\;k=K,k^{\prime }\neq K \end{array}\right.$$

*where* $x_{p}=x\left(\gamma_{K},\lambda_{K}\right)$ *is the solution to the equation:*

$$1-x_{p}=\gamma_{K}\left[1-\lambda_{K}\int {\left(x_{p}t+\lambda_{K}\right)}^{-1}dH_{K}(t)\right]$$

*Then*

$${𝒴}_{kk^{\prime }}-{\hat{𝒴}}_{kk^{\prime }}\to_{a.s.}0.$$

## 7 Real Data

### 7.1 Proteomics-based Prediction of 10-year Cardiovascular Disease Risk

We utilize the data set from the Chronic Renal Insufficiency Cohort (CRIC) study to evaluate the performance of the proposed transfer learning methods. The dataset comprises observations from 2, 182 subjects who had not had any cardiovascular disease at the baseline, with 4, 830 protein measurements collected. The number of subjects are 272, 280, 333, 326, 409, 180, 382 respectively for seven university sites. Our goal is to build a classification model for 10-year cardiovascular disease risk based on the baseline plasma proteomic data. We hypothesize that while the mechanisms by which proteins predict events are related across these sites, they are not identical. Consequently, we sequentially designate each site as the target population, with the remaining sites serving as auxiliary populations, aiming to enhance prediction accuracy using TL-RDA or TLP-RDA.

For each target population, approximately 20% of the patients were set aside as a testing dataset, while the remaining 80% were used for training. We ensured that the proportion of events was consistent between the training and testing datasets (~7%). A univariate filtering procedure was applied to the training dataset using two-sample t-tests, and we evaluated the benefits of transfer learning using the top 500, 1000, and 1500 proteins. All predictors are centered and scaled within each sites, using training dataset information only. We considered all four variants of transfer learning RDA: TL-RDA with optimal estimation or prediction weights and TLP-RDA with optimal estimation/prediction weights. The “optimal weights” were estimated as instructions given by remark 4.7. The tuning parameters λ for each model were selected independently by four-fold cross-validations over a length 9 logarithmically spaced grid ranges from 0.01 to 100. The domain specific penalization is obtained by $\lambda_{k}=\lambda \gamma_{k}/\alpha_{k}^{2}$. We refit each model on full training data with chosen λ and test them on the held-out testing dataset. All transfer learning weights are estimated based on empirical estimators provided in the theorems.

The testing AUCs were then compared with those obtained from competing methods, namely naive RDA and pool-RDA. As implied by their names, naive RDA is fitted using only the target population data, while pool-RDA is fitted on data pooled from all populations. These procedures were repeated 25 times, and we report the average AUCs and standard deviations of the AUCs in Figure 4.

It is evident that the transfer learning RDA methods outperform the others in the vast majority of cases. This demonstrates that not only are TL-RDAs capable of borrowing information to improve prediction accuracy, but they also do so more efficiently than simply pooling the data. Notably, TLP-RDA with optimal prediction weights outperforms the other methods most frequently. The amount of improvement brought by transfer learning also seemingly increase as we include more proteins in the model. Additionally, we observe that methods based on pooled sample covariance tend to perform better when the number of features p is larger, consistent with Proposition 5.2.

### 7.2 Lipid traits classification using genotype data

We also evaluate the proposed transfer-learning estimators using a data set from Penn Medicine BioBank, which contains both genome-wide genotype data and the electronic health record–derived phenotypes. We focus on three lipid traits with substantial genetic signal, high-density lipoprotein cholesterol (HDL), low-density lipoprotein cholesterol (LDL), and triglycerides (TRI), and dichotomize each outcome using guideline-based clinical cutoffs (Grundy et al., 2019, 2005; NCEP, 2002). To emulate settings where related auxiliary traits are available, we analyze each trait in turn as the target and use the remaining two traits as sources. The analytic cohort contains approximately 12000 individuals, partitioned into three trait-specific datasets. For each target analysis, feature screening is performed solely on the target training split via univariate association tests, and the top 1000, 2000, and 3000 nucleotide polymorphism (SNPs) (ranked by significance) are retained as the predictors.

All regularization hyperparameters were tuned exactly as in the proteomics experiment, using the same grid of candidate values. Figure 5 reports averaged AUCs and their standard deviations on held-out test sets for the proposed methods and the baselines. The statistics are based on 25 independent train / test splits and cross-validations. Across most configurations, Trans-Ridge improves upon target-only ridge regression. In contrast to the proteomics based prediction across different clinical sites in previous Section, the TL-RDA methods outperform the TLP-RDA variants based on pooled sample-covariance estimators. This ordering is again consistent with Proposition 5.2, which predicts an advantage for TL-RDA in the regime of small $\gamma_{k}$, the regime relevant to the present data.

## 8 Discussion

We have developed methods of transfer learning for classification using regularized random effects linear discriminant analysis. In this approach, the discriminant direction is estimated through a weighted combination of the regularized estimates of discriminant directions derived from both the target and source models. By leveraging results from random matrix theory, we have demonstrated that the weights, as well as the classification error rate, can be accurately estimated using the data. Our findings highlight that the optimal choice of weights depends critically on the underlying true models and the presence or absence of distributional shifts between the testing and training data in the target model.

Through comprehensive empirical evaluations, we have illustrated the practical utility of the proposed methods. Specifically, we applied the transfer learning approach to predict 10-year cardiovascular disease risk using high-dimensional protein expression data and to the lipid trait prediction problem with genotype data. The results demonstrate that incorporating information from related source datasets substantially improved classification performance compared to target-only models or models based on pooled data. This improvement underscores the importance of accounting for shared information across datasets while accommodating potential distributional differences. In addition, the theoretical guidance for selecting among the four candidate weighting schemes is supported by the empirical evidence. In the high-dimensional proteomics application, pooled-covariance weighting performs the best, consistent with its variance-reduction advantage when signals are diffuse. For the genotype data, where target and source data includes correlated but different outcomes, weights optimized for estimation typically outperform those optimized for prediction. When the requisite assumptions are uncertain, we recommend selecting among the four schemes using cross-validation.

In terms of computational scalability, TL-RDA has the same leading complexity as running the K source RDA models. To compute RDA directions with naive algorithms, forming the covariances costs $O\left(Kn_{k}p^{2}\right)$ and the K+1 matrix inversions cost $O\left(Kp^{3}\right)$. Solving and estimating the optimal weights are equivalent to at most two linear systems and one eigen-problem of size K×K. This $O\left(K^{3}\right)$ overhead is negligible because K≪p in our applications. In addition, all heavy computations are performed locally within each domain. Each domain returns only its RDA direction and a few scalar trace statistics required by Theorem 4.5 and Theorem 4.10. The central node only needs to combine these O(Kp) and O(K) summaries to obtain the weight vector. Moreover, because each inversion has the ridge form ${\left({\hat{\Sigma }}_{k}+\lambda I_{p}\right)}^{-1}$, we can apply Woodbury and perform a Cholesky factorization on an $n_{k}\times n_{k}$ system, thereby eliminating the cubic dependence on p and reducing per-domain cost to $O\left(n_{k}^{3}+n_{k}p\right)$ when $n_{k}\ll p$.

This paper focuses on two-class LDA models. A promising direction for future research is extending these methods to multi-class classification within the framework of transfer learning. Other interesting avenues include exploring this problem under privacy or communication constraints, as has been recently studied for other classification methods, or analyzing LDA trained with mixed in-distribution and out-of-distribution samples (see, e.g., Auddy et al., 2024; De Silva et al., 2023). Also, we have assumed the number of source domains K is small compared to number of observations and predictors ${(n}_{k},p)$. If instead K is very large, source selection becomes necessary. As noted in Section 4.4, the optimal weight vectors solves a least-squares problem in which the Bayes discriminant direction is regressed on the collection of domain-specific discriminant directions. When the number of sources K grows to the same order as n and p, this becomes a high-dimensional regression and variable-selection problem. A simple heuristic is to retain only those sources whose discriminant directions have the largest absolute correlations with the target estimate; however, this ignores cross-source dependencies and may discard complementary information. A natural extension is to add a ridge-type weight penalization $\|w{\|}_{2}^{2}$ on criterion 3 or criterion 4. The resulting estimator can still be computed in closed form via the machinery of Theorems 4.5 and Theorems 4.10. Developing principled, sparsity-inducing penalties or screening rules that account for inter-source correlations and analyzing their theoretical guarantees, remains an interesting direction for future research.

## Supplementary Material

1

## Figures and Tables

**Figure 1:**
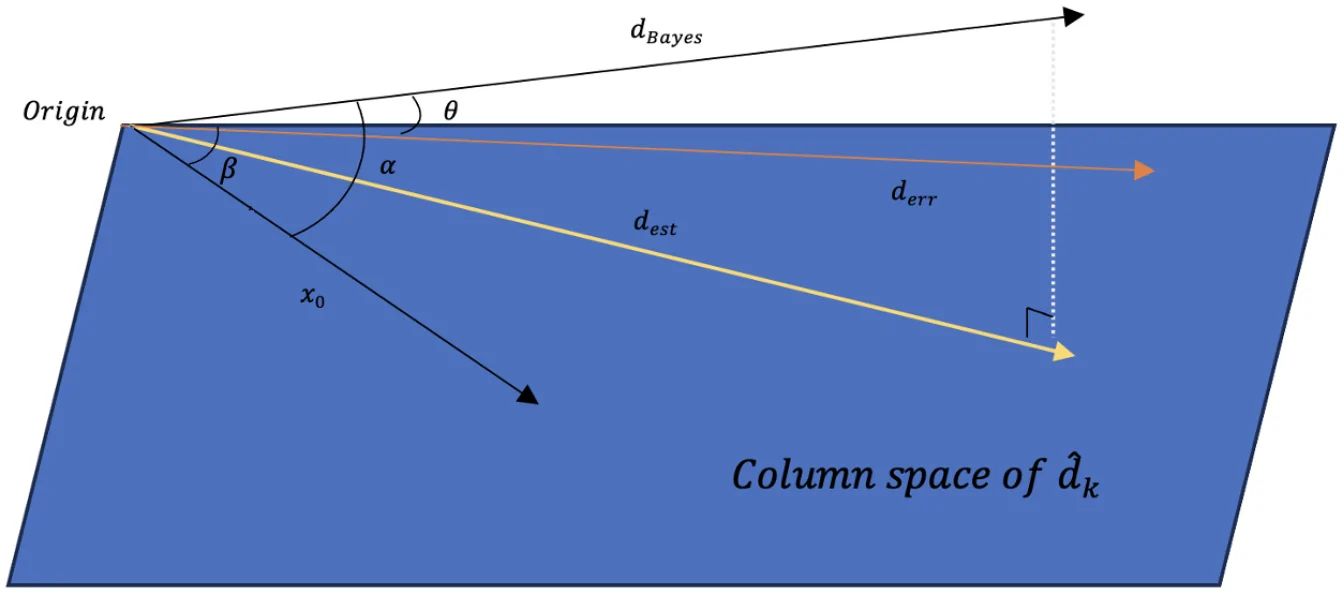
Geometric interpretations of optimal weights

**Figure 2:**
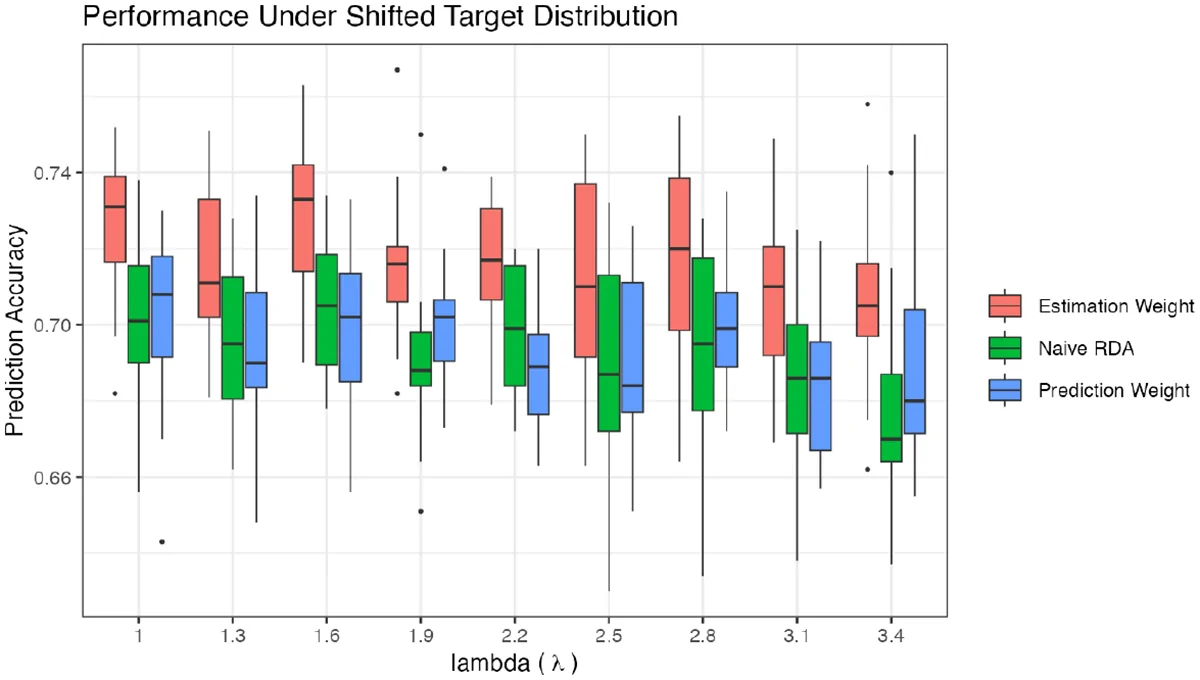
TL-RDA with the optimal estimation weight outperforms regular RDA and TL-RDA with the optimal prediction weight when the target distribution changes.

**Figure 3:**
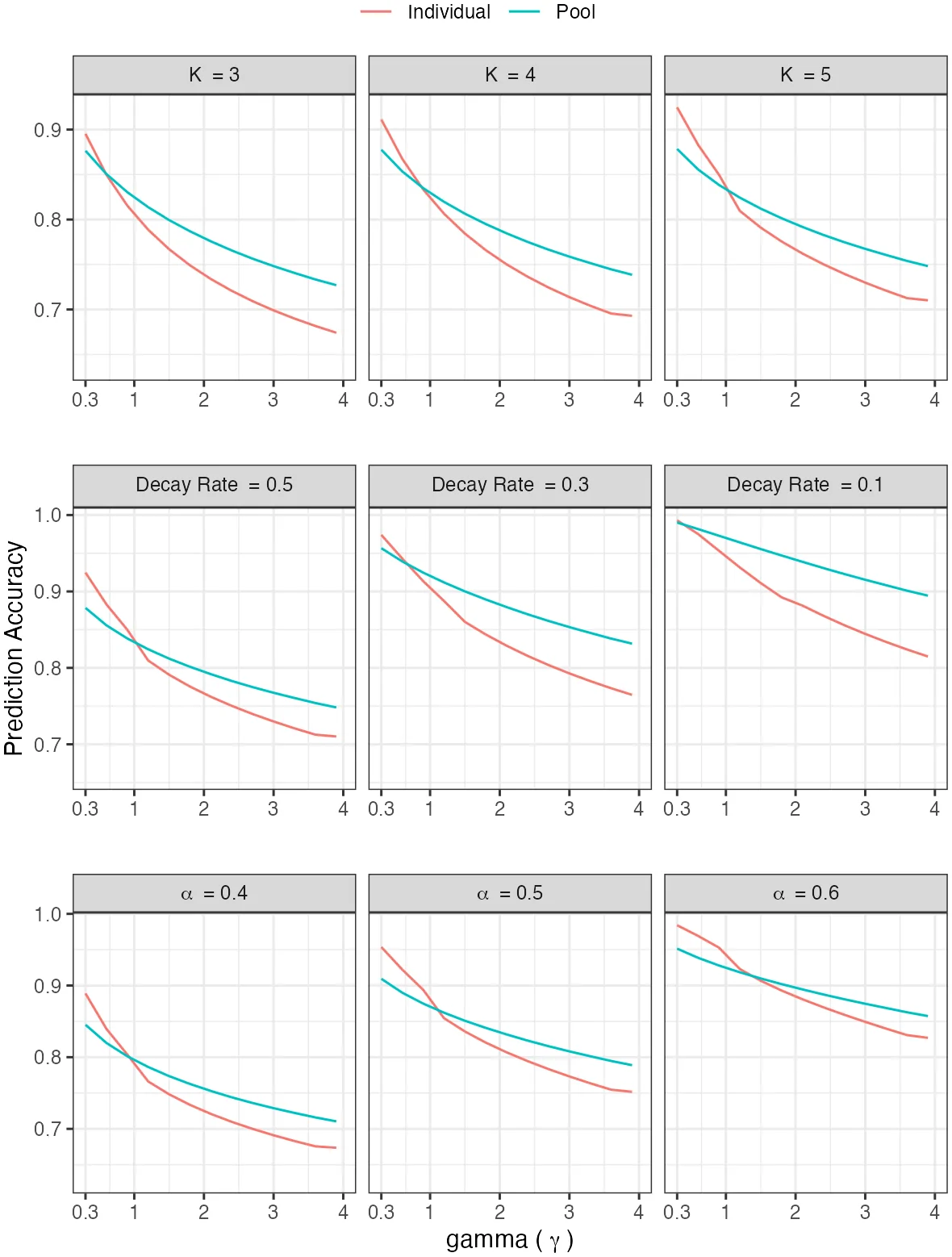
TLP-RDA outperforms TL-RDA when γ is large under general setups.

**Figure 4:**
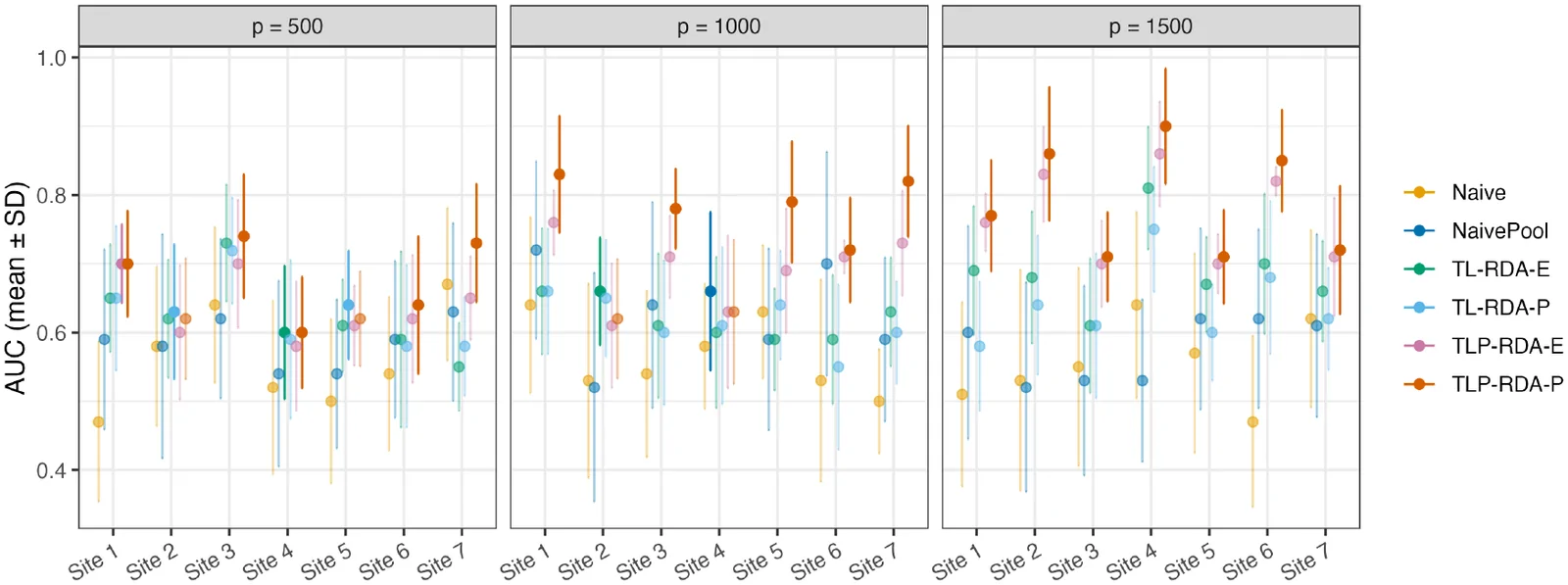
AUC ± SD on testing data for 10-year cardiovascular disease risk for each of the 7 clinical sites in the CRIC cohort using 500, 1000 and 1500 proteins, respectively. TL-RDA-E, TL-RDA-P, TLP-RDA-E and TLP-RDA-P denote TL-RDA with optimal estimation/prediction weights and TLP-RDA with optimal estimation/prediction weights, respectively.

**Figure 5:**
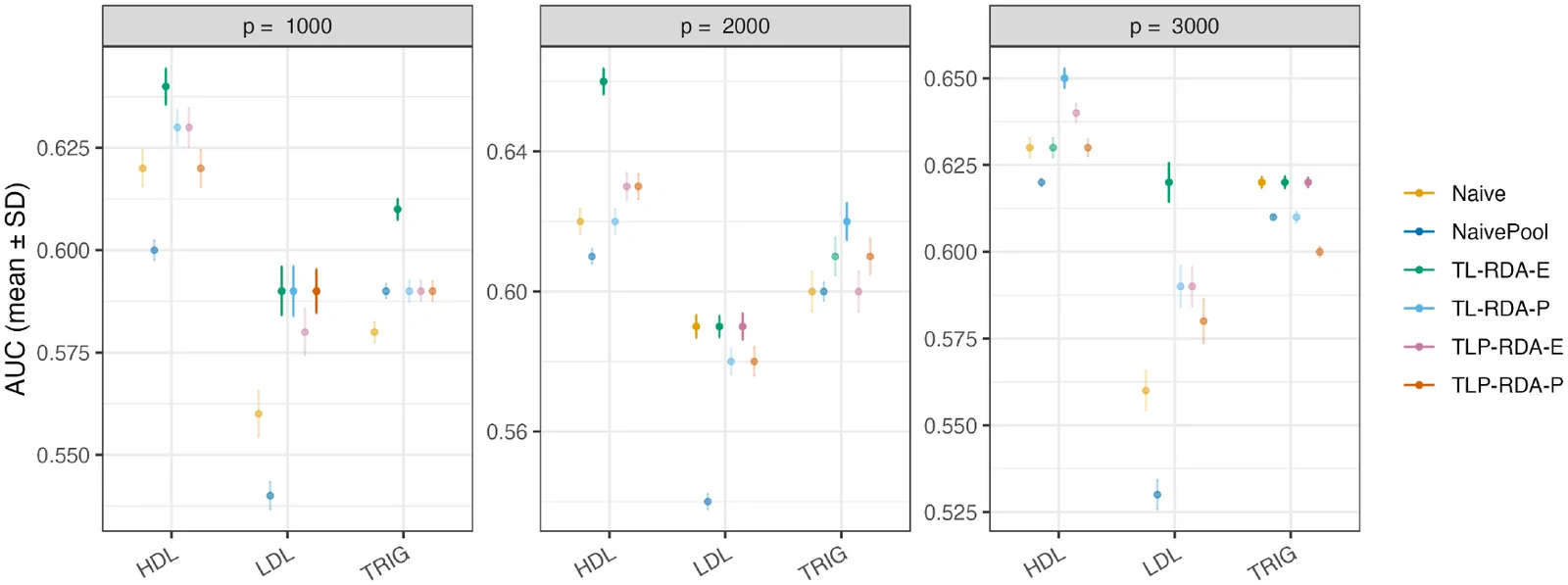
AUC ± SD on testing data for HDL/LDL/Trig using 1000, 2000 and 3000 SNPs, respectively. TL-RDA-E, TL-RDA-P, TLP-RDA-E and TLP-RDA-P denote TL-RDA with optimal estimation/prediction weights and TLP-RDA with optimal estimation/prediction weights, respectively.
