# Supplementary material for: Transfer Learning via Regularized Random-effects Linear Discriminant Analysis
Source: J Mach Learn Res. Author manuscript; Available in PMC 2026 Oct 7. (PMC13641122)
Supplement: Supplementary file 1 [file NIHMS2192745-supplement-1.pdf]

Appendix A. Appendix A

A.1 Proofs of Propositions

Proof [Proof of Proposition 4.8] Denote E as the random variable distributed according to the empirical spectral distribution F_γ. When $\gamma < 1$, E is supported on a compact set bounded away from 0 (Bai and Silverstein, 2010). Therefore, we can take the limit of $m_{F_\gamma}(-\lambda)$ as $\lambda \to 0$. Recall the Marchenko–Pastur equation

$$m_{F_\gamma}(z) = \int_{t=0}^{\infty} \frac{dH(t)}{t(1-\gamma-\gamma z m_{F_\gamma}(z)) - z}$$

We find $m_{F_\gamma}(0) = \int 1/[t(1-\gamma)]dH(t)$ or equivalently $\text{tr}(\widehat{\Sigma}^{-1})/p \to_{a.s.} \mathbb{E}(T^{-1})/(1-\gamma)$. ∎

Proof [Proof of Proposition 4.11] The proof is identical to the proof of Proposition 6.4 which considers the more general case of unequal covariance matrices Σ_k. ∎

Proof [Proof of Proposition 4.14] Recall from Theorem 10 that the limiting prediction error is in the form of

$$\Phi\left(-\frac{\mathbf{w}^\top \mathbf{u}}{\sqrt{\mathbf{w}^\top A \mathbf{w}}}\right)$$

Any weight $\mathbf{w}$ with negative linear term $\mathbf{w}^\top \mathbf{u}$ has higher prediction error than the weight with positive linear term. Without loss of generality, we assume $\mathbf{w}^\top v$ to be positive. Then we know

$$\mathbf{w}^* = \arg\max_{\mathbf{w}} \frac{\mathbf{w}^\top \mathbf{u}}{\sqrt{\mathbf{w}^\top \mathcal{A} \mathbf{w}}} = \arg\max \frac{\mathbf{w}^\top \mathbf{u}\mathbf{u}^\top \mathbf{w}}{\mathbf{w}^\top \mathcal{A} \mathbf{w}}$$

this becomes the generalized Rayleigh quotient problem and the solution is given by the first unit eigenvector of $\mathcal{A}^{-1}\mathbf{u}\mathbf{u}^\top$, which is a rank one matrix, and hence

$$\mathbf{w}^* = \frac{1}{\|\mathcal{A}^{-1}\mathbf{u}\|}\mathcal{A}^{-1}\mathbf{u}, \text{ which implies, } Err_{opt} := \Phi\left(-\sqrt{\mathbf{u}^\top \mathcal{A}^{-1}\mathbf{u}}\right).$$

Note the standardization factor in $\mathbf{w}$ is not necessary due to the form of $Err(\mathbf{w})$. The proof finishes as one recognizes the $\mathcal{A} = \mathcal{A}^P + \mathcal{R}^P$ and $\mathbf{u} = \mathbf{u}^P$. We can prove the statement for $\mathbf{w}_P^P$ in the same manner. ∎

Proof [Proof of Proposition 4.15] We only need to prove $\mathcal{A}^E + \mathcal{R}^E, \mathcal{A}^P + \mathcal{R}^P, \mathcal{A}_P^E + \mathcal{R}_P^E, \mathcal{A}_P^P + \mathcal{R}_P^P$ are positive definite, therefore, invertible. Looking at the individual sample covariance matrix cases, taking individual prediction weight as an example. We will firstly show that $\mathcal{A}^P$ is positive semi-definite. Note that

$$\begin{aligned}
(\mathcal{A}^P)_{kk'} &= \alpha_k \alpha_{k'} \rho_{kk'} \text{tr}[\Sigma(\widehat{\Sigma}_k + \lambda_k \mathbb{I}_p)^{-1}(\widehat{\Sigma}_{k'} + \lambda_{k'}\mathbb{I}_p)^{-1}]/p \\
&= \alpha_k \alpha_{k'} \rho_{kk'} \text{tr}[\mathbf{M}_k^\top \mathbf{M}_{k'}]/p \\
&= \alpha_k \alpha_{k'} \rho_{kk'} \mathbf{v}_{M_k}^\top \mathbf{v}_{M_{k'}}/p
\end{aligned}$$

where $\mathbf{M}_k = \Sigma^{1/2}(\widehat{\Sigma}_k + \lambda_k \mathbb{I}_p)^{-1}$ and $\mathbf{v}_{M_k} = \mathrm{vec}(\mathbf{M}_k) \in \mathbb{R}^{K^2}$. For any $\mathbf{x} \in \mathbb{R}^K$, $\mathbf{x} \neq \mathbf{0}$, we have

$$\begin{aligned}\mathbf{x}^\top \mathcal{A}^P \mathbf{x} &= \sum_{k,k'} (\alpha_k x_k)(\alpha_{k'} x_{k'}) \rho_{kk'} \mathbf{v}_{M_k}^\top \mathbf{v}_{M_{k'}}/p \\ &= \mathbf{y}^\top \mathcal{A}^P \mathbf{y}/p\end{aligned}$$

where $\mathbf{y} = ((\alpha_i x_i))_i \in \mathbb{R}^K$. Since $\alpha_i > 0$ for all i, we have $\mathbf{y} \neq \mathbf{0}$ if and only if $\mathbf{x} \neq \mathbf{0}$. It is thus enough to show that $\mathcal{A}^{(3)}$, defined through

$$(\overline{\mathcal{A}})_{ij} = \ \rho_{ij} \mathbf{v}_{M_i}^\top \mathbf{v}_{M_j}$$

is positive-semi definite. To this end, note that

$$\begin{aligned}\mathbf{y}^\top \overline{\mathcal{A}} \mathbf{y} &= \ \sum_{i,j} \rho_{ij} (y_i \mathbf{v}_{M_i})^\top (y_j \mathbf{v}_{M_j}) \\ &= \ \sum_{i,j} \rho_{ij} (\mathbf{c}_i)^\top \mathbf{c}_j = \mathrm{tr}(\Sigma_\delta \mathbf{C}^\top \mathbf{C}) \\ &= \mathrm{tr}(\mathbf{C} \Sigma_\delta^{1/2} \Sigma_\delta^{1/2} \mathbf{C}^\top) \\ &= \|\boldsymbol{\rho}^{1/2} \mathbf{C}^\top\|_{\mathrm{F}}^2 \geq 0.\end{aligned}$$

Here $\mathbf{C} \in \mathbb{R}^{K^2 \times K}$ matrix with columns $\mathbf{c}_i = y_i \mathbf{v}_{M_i}$, for $i = 1, \ldots, K$. This shows that $\mathcal{A}^{(1)}$ is positive semi-definite since for any $\mathbf{x} \in \mathbb{R}^K$, $\mathbf{x} \neq \mathbf{0}$ we have

$$\mathbf{x}^\top \mathcal{A}^P \mathbf{x} = \mathbf{y}^\top \overline{\mathcal{A}} \mathbf{y} \geq 0. \tag{13}$$

Now note that $\mathcal{A}^{(2)}$ is a diagonal matrix with non-negative elements, this finishes the proof of the positive definiteness of $\mathcal{A}^P + \mathcal{A}^R$. To prove the case of estimation weight, one can simply define $\mathbf{M}_k = (\widehat{\Sigma}_k + \lambda_k \mathbb{I}_p)^{-1}$.

Looking at the pooled sample covariance matrix cases, taking pooled prediction weight as an example, we would have

$$\begin{aligned}\mathcal{A}_P^P &= c^A \Sigma_{\alpha\delta} \\ \Sigma_{\alpha\delta} &= mat\,[\rho_{kk'} \alpha_k \alpha_{k'}] \\ c^A &= \mathrm{tr}[(\widehat{\Sigma}_P + \lambda I_p)^{-2} \Sigma]/p\end{aligned}$$

Since $\mathcal{R}^P$ has non-negative diagonal entries and $c^A > 0$, we only need to prove the positive semi-definiteness of $\Sigma_{\alpha\delta}$. Consider the quadratic form $x^\top \Sigma_{\alpha\delta} x$:

$$x^\top \Sigma_{\alpha\delta} x = \sum_{i=1}^K \sum_{j=1}^K \alpha_i x_i \alpha_j (\Sigma_{\alpha\delta})_{ij} x_j = \left(\sum_{i=1}^K \alpha_i x_i\right) \left(\sum_{j=1}^K \alpha_j \Sigma_{ij} x_j\right)$$

Define $y_i = \alpha_i x_i$. Then we have:

$$x^\top \Sigma_{\alpha\delta} x = \sum_{i=1}^K y_i \sum_{j=1}^K (\Sigma_\delta)_{ij} y_j = y^\top \Sigma y$$

Since Σ_δ is positive semi-definite, $\mathcal{A}_P^P + \mathcal{R}_P^P$ will be positive definite. We can prove the positive definiteness of $\mathcal{A}_P^E + \mathcal{R}_P^E$ in exactly the same way. ■

Proof [Proof of Proposition 5.1] Since the maximum value of the dot product of two vectors is achieved when the two vectors are aligned, the maximization of the min-max problem is solved when x_0 is in the same direction as the error with $\mathbb{E}_P(\|x_0\|_2) = c$. Since x_0 is independent of the estimation error vector $(\sum_{k=1}^K w_k \widehat{d}_k - d_{Bayes})^\top$, we have the solution of the max problem as

$$x_0^* = c\frac{d_{Bayes} - \sum_{k=1}^K w_k \widehat{d}_k}{\|d_{Bayes} - \sum_{k=1}^K w_k \widehat{d}_k\|} \text{ with probability 1.}$$

This leads to the estimation risk minimization problem with criteria (3). ■

Proof [Proof of Proposition 5.2] For simplicity, we assume $\Sigma = \mathbb{I}_p$, in which case the optimal esimation weight coincides with the optimal prediction weights. In addition, we have the following simplifications

Corollary A.1 (Simplification of Limiting Terms for Individual Sample Covariance Matrix) *Recall the form of limiting prediction error given a weight vector* $\mathbf{w}$ (10)*. We have the term* $\mathcal{A}$ *simplified as following. For* $k = 1, \cdots, K$*,*

$$\mathcal{A}_{kk} = \alpha_k^2 m_k'(-\lambda_k) + \gamma_k m_k'(-\lambda_k)$$

else when $k \neq k'; k, k' = 1, \cdots, K$

$$\mathcal{A}_{kk'} = \rho_{kk'}\alpha_k\alpha_{k'} m_k(-\lambda_k) m_{k'}(-\lambda_{k'})$$

and we have $\mathbf{u}^E = vec[\rho_{kK}\alpha_k\alpha_K m_{F_{\gamma_k}}(-\lambda_k)]$*,* $\mathcal{A}_{kk'}^E + \mathcal{R}^E = \mathcal{A}$*.*

Corollary A.2 (Simplification of Limiting Terms for Pooled Sample Covariance Matrix) *Define the weighted covariance matrix as* $\Sigma_\delta = mat[\rho_{kk'}\alpha_k\alpha_{k'}], \rho_{kk} = 1$*. We have the term* $\mathcal{A}$ *simplified as:*

$$\mathcal{A}_P = \Sigma_\delta m'_{F_{\gamma/K}}(-\lambda) + \gamma_k m'_{F_{\gamma/K}}(-\lambda)\mathbb{I}_K$$

and $\mathbf{u}_P^E = vec[\rho_{kK}\alpha_k\alpha_K m_{F_{\gamma/K}}(-\lambda)]$*,* $\mathcal{A}_P^E + \mathcal{R}_P^E = \mathcal{A}_P$*.*

We can now write the limiting error of using individual sample covariance matrix and pooled covariance matrix as

$$Err_{ind} = \Phi\left(-\sqrt{(\mathbf{u}^E)^\top \mathcal{A}^{-1}\mathbf{u}^E}\right) \text{ and } Err_{pool} = \Phi\left(-\sqrt{(\mathbf{u}_P^E)^\top \mathcal{A}_P^{-1}\mathbf{u}_P^E}\right).$$

Recall that we have the explicit expressions

$$m_{F_\gamma}(-\lambda) = \frac{-(1-\gamma+\lambda) + \sqrt{(1-\gamma+\lambda)^2 + 4\gamma\lambda}}{2\gamma\lambda} \tag{14}$$

$$m'_{F_\gamma}(-\lambda) = \frac{m^2_{F_\gamma}(-\lambda)[1+\gamma m_{F_\gamma}(-\lambda)]}{1+\gamma\lambda m^2_{F_\gamma}(-\lambda)}. \tag{15}$$

For some $r > (1-\gamma)_+/\gamma + c$ (for some small constant c to be chosen later), we choose λ as follows:

$$\begin{aligned} 1-\gamma+\lambda = r\gamma - \lambda/r \iff& (1+1/r)\lambda = (r+1)\gamma - 1 \\ \iff& \lambda_* = r\left(\gamma - \frac{1}{r+1}\right) \end{aligned}$$

The following proposition contains a specific choice of r and some inequalities that will be useful for the rest of this proof. The proof of this proposition is immediate and hence omitted.

Proposition A.3 *The following statements hold for $r > (1-\gamma)_+/\gamma + c$ (for some constant $c > 0$):*

1. $m_\gamma(-\lambda_*) = \frac{1}{r\gamma}$
2. $\frac{m'_{F_\gamma}(-\lambda_*)}{m^2_{F_\gamma}(-\lambda_*)} = \frac{\gamma(1+r)^2}{\gamma(1+r)^2-1}$
3. $\Delta := \frac{m'_{F_\gamma}(-\lambda_*)}{m^2_{F_\gamma}(-\lambda_*)} - 1 = \frac{1}{\gamma(1+r)^2-1} \le \frac{1}{\gamma-1}$

We now look at the quadratic term in Err_{pool} and assume $\rho_{kk'} = \rho$. Then by Corollary A.2 we have

$$\begin{aligned} \mathcal{A}_P &= \Sigma_\delta m'_{F_{\gamma/K}}(-\lambda) + m'_{F_{\gamma/K}}(-\lambda)\mathrm{diag}(\{\gamma_k : 1\le k\le K\}) \\ &= m'_{F_{\gamma/K}}(-\lambda)\left[\rho\widetilde{\boldsymbol\alpha}\widetilde{\boldsymbol\alpha}^\top + \mathrm{diag}(\{(1-\rho)\alpha_k^2 + \gamma_k : 1\le k\le K\})\right] \\ &=: m'_{F_{\gamma/K}}(-\lambda)\left[\rho\widetilde{\boldsymbol\alpha}\widetilde{\boldsymbol\alpha}^\top + D_{\rho,\widetilde{\boldsymbol\gamma}}\right] \end{aligned}$$

and

$$\mathbf{u}_P^E = vec[\rho_{kK}\alpha_K\alpha_k m_{F_{\gamma/K}}(-\lambda)] = m_{F_{\gamma/K}}(-\lambda)\alpha_K[\rho\widetilde{\boldsymbol\alpha} + (1-\rho)\alpha_K\mathbf{e}_K].$$

By the Sherman Morrison formula, we have:

$$\mathcal{A}_P^{-1} = \frac{1}{m'_{F_{\gamma/K}}(-\lambda)}\left[D^{-1}_{\rho,\widetilde{\boldsymbol\gamma}} - \frac{\rho}{1+\rho\sum_{k=1}^K \frac{\alpha_k^2}{(1-\rho)\alpha_k^2+\gamma_k}} D^{-1}_{\rho,\widetilde{\boldsymbol\gamma}}\widetilde{\boldsymbol\alpha}\widetilde{\boldsymbol\alpha}^\top D^{-1}_{\rho,\widetilde{\boldsymbol\gamma}}\right].$$

Consequently:

$$\begin{aligned} \widetilde{\boldsymbol\alpha}^\top\mathcal{A}_P^{-1}\widetilde{\boldsymbol\alpha} &= \frac{1}{m'_{F_{\gamma/K}}(-\lambda)}\cdot\frac{\widetilde{\boldsymbol\alpha}^\top D^{-1}_{\rho,\widetilde{\boldsymbol\gamma}}\widetilde{\boldsymbol\alpha}}{1+\rho\widetilde{\boldsymbol\alpha}^\top D^{-1}_{\rho,\widetilde{\boldsymbol\gamma}}\widetilde{\boldsymbol\alpha}} \\ \widetilde{\boldsymbol\alpha}^\top\mathcal{A}_P^{-1}\mathbf{e}_K &= \frac{1}{m'_{F_{\gamma/K}}(-\lambda)}\cdot\frac{\widetilde{\boldsymbol\alpha}^\top D^{-1}_{\rho,\widetilde{\boldsymbol\gamma}}\mathbf{e}_K}{1+\rho\widetilde{\boldsymbol\alpha}^\top D^{-1}_{\rho,\widetilde{\boldsymbol\gamma}}\widetilde{\boldsymbol\alpha}} = \frac{\alpha_K}{m'_{F_{\gamma/K}}(-\lambda)}\cdot\frac{\mathbf{e}_K^\top D^{-1}_{\rho,\widetilde{\boldsymbol\gamma}}\mathbf{e}_K}{1+\rho\widetilde{\boldsymbol\alpha}^\top D^{-1}_{\rho,\widetilde{\boldsymbol\gamma}}\widetilde{\boldsymbol\alpha}} \end{aligned}$$

$$
\begin{aligned}
\mathbf{e}_K^\top \mathcal{A}_P^{-1}\mathbf{e}_K &= \frac{1}{m'_{F_{\gamma/K}}(-\lambda)} \cdot \left[\mathbf{e}_K^\top D_{\rho,\widetilde{\gamma}}^{-1}\mathbf{e}_K - \frac{\alpha_K^2}{1+\rho\widetilde{\boldsymbol{\alpha}}^\top D_{\rho,\widetilde{\gamma}}^{-1}\widetilde{\boldsymbol{\alpha}}}(\mathbf{e}_K^\top D_{\rho,\widetilde{\gamma}}^{-1}\mathbf{e}_K)^2\right] \\
&= \frac{\mathbf{e}_K^\top D_{\rho,\widetilde{\gamma}}^{-1}\mathbf{e}_K}{m'_{F_{\gamma/K}}(-\lambda)} \cdot \left[\frac{1+\rho\widetilde{\boldsymbol{\alpha}}^\top D_{\rho,\widetilde{\gamma}}^{-1}\widetilde{\boldsymbol{\alpha}} - \rho\alpha_K^2\mathbf{e}_K^\top D_{\rho,\widetilde{\gamma}}^{-1}\mathbf{e}_K}{1+\rho\widetilde{\boldsymbol{\alpha}}^\top D_{\rho,\widetilde{\gamma}}^{-1}\widetilde{\boldsymbol{\alpha}}}\right]
\end{aligned}
$$

which, upon writing $\widetilde{\boldsymbol{\alpha}}_{/K} = (\alpha_1\,\alpha_2\,\ldots\,\alpha_{K-1}\,0)^\top$, implies that:

$$
\begin{aligned}
&(\mathbf{u}_P^E)^\top \mathcal{A}_P^{-1}\mathbf{u}_P^E \\
&= \frac{\alpha_K^2\cdot m^2_{F_{\gamma/K}}(-\lambda)}{m'_{F_{\gamma/K}}(-\lambda)}\left[\rho^2\widetilde{\boldsymbol{\alpha}}^\top\mathcal{A}_P^{-1}\widetilde{\boldsymbol{\alpha}} + 2\rho(1-\rho)\alpha_K\widetilde{\boldsymbol{\alpha}}^\top\mathcal{A}_P^{-1}\mathbf{e}_K + (1-\rho)^2\alpha_K^2\mathbf{e}_K^\top\mathcal{A}_P^{-1}\mathbf{e}_K\right] \\
&= \frac{m^2_{F_{\gamma/K}}(-\lambda)}{m'_{F_{\gamma/K}}(-\lambda)}\cdot\frac{\alpha_K^2}{1+\rho\widetilde{\boldsymbol{\alpha}}^\top D_{\rho,\widetilde{\gamma}}^{-1}\widetilde{\boldsymbol{\alpha}}}\times \\
&\quad\times\left[\rho^2\widetilde{\boldsymbol{\alpha}}^\top D_{\rho,\widetilde{\gamma}}^{-1}\widetilde{\boldsymbol{\alpha}} + (1-\rho)\alpha_K^2\mathbf{e}_K^\top D_{\rho,\widetilde{\gamma}}^{-1}\mathbf{e}_K[2\rho+1-\rho+\rho(1-\rho)\widetilde{\boldsymbol{\alpha}}_{/K}^\top D_{\rho,\widetilde{\gamma}}^{-1}\widetilde{\boldsymbol{\alpha}}_{/K}]\right] \\
&= \frac{m^2_{F_{\gamma/K}}(-\lambda)}{m'_{F_{\gamma/K}}(-\lambda)}\cdot\frac{\alpha_K^2}{1+\rho\widetilde{\boldsymbol{\alpha}}^\top D_{\rho,\widetilde{\gamma}}^{-1}\widetilde{\boldsymbol{\alpha}}}\times \\
&\quad\times\left[\alpha_K^2\mathbf{e}_K^\top D_{\rho,\widetilde{\gamma}}^{-1}\mathbf{e}_K + \rho^2\widetilde{\boldsymbol{\alpha}}_{/K}^\top D_{\rho,\widetilde{\gamma}}^{-1}\widetilde{\boldsymbol{\alpha}}_{/K} + \rho(1-\rho)^2(\alpha_K^2\mathbf{e}_K^\top D_{\rho,\widetilde{\gamma}}^{-1}\mathbf{e}_K)\widetilde{\boldsymbol{\alpha}}_{/K}^\top D_{\rho,\widetilde{\gamma}}^{-1}\widetilde{\boldsymbol{\alpha}}_{/K}\right] \qquad (16)
\end{aligned}
$$

Let us now look at the quadratic term of the individual directions, once again assuming $\rho_{kk'} = \rho$. We make another simplifying assumption at this point:
Assumption: $\gamma_k = \gamma$ and $\lambda_k = \lambda$ for $k = 1, \ldots, K$. Then the population specific covariance matrix based quadratic form reduces through the following calculation.

By Corollary A.1 we now have

$$
\begin{aligned}
\mathcal{A} &= m^2_{F_\gamma}(-\lambda)\left[\rho\widetilde{\boldsymbol{\alpha}}\widetilde{\boldsymbol{\alpha}}^\top + \mathrm{diag}\left(\left\{\left(\frac{m'_{F_\gamma}(-\lambda)}{m^2_{F_\gamma}(-\lambda)} - \rho\right)\alpha_k^2 + \frac{m'_{F_\gamma}(-\lambda)\gamma}{m^2_{F_\gamma}(-\lambda)} : 1\le k\le K\right\}\right)\right] \\
&=: m^2_{F_\gamma}(-\lambda)\left[\rho\widetilde{\boldsymbol{\alpha}}\widetilde{\boldsymbol{\alpha}}^\top + D_{\rho,\gamma,ind}\right]
\end{aligned}
$$

and

$$
\mathbf{u}^E = vec[\rho_{kK}\alpha_K\alpha_k m_{F_\gamma}(-\lambda)] = m_{F_\gamma}(-\lambda)\alpha_K[\rho\widetilde{\boldsymbol{\alpha}} + (1-\rho)\alpha_K\mathbf{e}_K].
$$

Then following the exact steps leading to (16) we arrive at:

$$
\begin{aligned}
&(\mathbf{u}^E)^\top\mathcal{A}^{-1}\mathbf{u}^E \\
&= \frac{\alpha_K^2}{1+\rho\widetilde{\boldsymbol{\alpha}}^\top D_{\rho,\gamma,ind}^{-1}\widetilde{\boldsymbol{\alpha}}}\times \\
&\quad\times\left[\alpha_K^2\mathbf{e}_K^\top D_{\rho,\gamma,ind}^{-1}\mathbf{e}_K + \rho^2\widetilde{\boldsymbol{\alpha}}_{/K}^\top D_{\rho,\gamma,ind}^{-1}\widetilde{\boldsymbol{\alpha}}_{/K} + \rho(1-\rho)^2(\alpha_K^2\mathbf{e}_K^\top D_{\rho,\gamma,ind}^{-1}\mathbf{e}_K)\widetilde{\boldsymbol{\alpha}}_{/K}^\top D_{\rho,\gamma,ind}^{-1}\widetilde{\boldsymbol{\alpha}}_{/K}\right]. \qquad (17)
\end{aligned}
$$

Now comparing Equations (16) and (17) we can determine whether the pooled or the individual covariance matrix builds a better estimator. We consider two special cases below:

Case 1 ($\rho = 1$): In this case we have from (16) and (17) that:

$$(\mathbf{u}_P^E)^\top \mathcal{A}_P^{-1} \mathbf{u}_P^E = \frac{m^2_{F_{\gamma/K}}(-\lambda')\alpha_K^2}{m'_{F_{\gamma/K}}(-\lambda')} \cdot \frac{\widetilde{\boldsymbol{\alpha}}^\top D^{-1}_{1,\widetilde{\boldsymbol{\gamma}}}\widetilde{\boldsymbol{\alpha}}}{1+\widetilde{\boldsymbol{\alpha}}^\top D^{-1}_{1,\widetilde{\boldsymbol{\gamma}}}\widetilde{\boldsymbol{\alpha}}}$$

and

$$(\mathbf{u}^E)^\top \mathcal{A}^{-1} \mathbf{u}^E = \frac{\alpha_K^2(\widetilde{\boldsymbol{\alpha}}^\top D^{-1}_{1,\gamma,ind}\widetilde{\boldsymbol{\alpha}})}{1+\widetilde{\boldsymbol{\alpha}}^\top D^{-1}_{1,\gamma,ind}\widetilde{\boldsymbol{\alpha}}}.$$

Note that by definition of $D_{\rho,\gamma,ind}$ we have

$$\begin{aligned}
\widetilde{\boldsymbol{\alpha}}^\top D^{-1}_{1,\gamma,ind}\widetilde{\boldsymbol{\alpha}} &= \sum_{k=1}^{K} \frac{\alpha_k^2}{\left(\frac{m'_{F_\gamma}(-\lambda)}{m^2_{F_\gamma}(-\lambda)} - \rho\right)\alpha_k^2 + \frac{m'_{F_\gamma}(-\lambda)\gamma}{m^2_{F_\gamma}(-\lambda)}} \\
&\le \frac{m^2_{F_\gamma}(-\lambda)}{m'_{F_\gamma}(-\lambda)} \sum_{k=1}^{K} \frac{\alpha_k^2}{\gamma} = \frac{m^2_{F_\gamma}(-\lambda)}{m'_{F_\gamma}(-\lambda)} \widetilde{\boldsymbol{\alpha}}^\top D^{-1}_{1,\widetilde{\boldsymbol{\gamma}}}\widetilde{\boldsymbol{\alpha}} \\
&= \frac{\gamma(1+r)^2-1}{\gamma(1+r)^2} \times \widetilde{\boldsymbol{\alpha}}^\top D^{-1}_{1,\widetilde{\boldsymbol{\gamma}}}\widetilde{\boldsymbol{\alpha}}
\end{aligned}$$

where the last line follows from our choice of λ. Since $f(t) = \frac{t}{1-t}$ is an increasing function of t it follows that:

$$\frac{\widetilde{\boldsymbol{\alpha}}^\top D^{-1}_{1,\gamma,ind}\widetilde{\boldsymbol{\alpha}}}{1+\widetilde{\boldsymbol{\alpha}}^\top D^{-1}_{1,\gamma,ind}\widetilde{\boldsymbol{\alpha}}} \le \frac{[\gamma(1+r)^2-1]\widetilde{\boldsymbol{\alpha}}^\top D^{-1}_{1,\widetilde{\boldsymbol{\gamma}}}\widetilde{\boldsymbol{\alpha}}}{\gamma(1+r)^2 + [\gamma(1+r)^2-1]\widetilde{\boldsymbol{\alpha}}^\top D^{-1}_{1,\widetilde{\boldsymbol{\gamma}}}\widetilde{\boldsymbol{\alpha}}} = \frac{[\gamma(1+r)^2-1]\sum_k \alpha_k^2}{\gamma^2(1+r)^2 + [\gamma(1+r)^2-1]\sum_k \alpha_k^2}.$$

On the other hand,

$$\frac{m^2_{F_{\gamma/K}}(-\lambda')}{m'_{F_{\gamma/K}}(-\lambda')} \cdot \frac{\widetilde{\boldsymbol{\alpha}}^\top D^{-1}_{1,\widetilde{\boldsymbol{\gamma}}}\widetilde{\boldsymbol{\alpha}}}{1+\widetilde{\boldsymbol{\alpha}}^\top D^{-1}_{1,\widetilde{\boldsymbol{\gamma}}}\widetilde{\boldsymbol{\alpha}}} = \frac{\gamma(1+r')^2-K}{\gamma(1+r')^2} \times \frac{\sum_k \alpha^2}{\gamma + \sum_k \alpha_k^2}.$$

Thus the pooled covariance matrix performs better when

$$\left(\gamma(1+r')^2 - K\right)\left(\gamma^2(1+r)^2 + [\gamma(1+r)^2-1]\sum_k \alpha_k^2\right) \ge \gamma(1+r')^2[\gamma(1+r)^2-1](\gamma + \sum_k \alpha_k^2)$$

which happens when

$$\begin{aligned}
&\left(\gamma(1+r')^2 - K\right)\left([\gamma(1+r)^2-1]\sum_k \alpha_k^2\right) - K\gamma^2(1+r)^2 \\
&\qquad\qquad \ge \gamma(1+r')^2[\gamma(1+r)^2-1](\sum_k \alpha_k^2) - \gamma^2(1+r')^2 \\
\iff\ & \gamma^2[(1+r')^2 - K(1+r)^2] \ge K\left([\gamma(1+r)^2-1]\sum_k \alpha_k^2\right).
\end{aligned}$$

i.e., when $\gamma \geq \gamma_*$ where γ_* is the largest value of γ for which equality holds in the above inequality. This follows since the coefficient of γ^2 on the LHS is positive, by our choice of r, r'.

Case 2 ($\rho = 0$): Once again using (16) and (17) we get that in this case:

$$(\mathbf{u}_P^E)^\top \mathcal{A}_P^{-1} \mathbf{u}_P^E = \frac{m^2_{F_{\gamma/K}}(-\lambda')\alpha_K^2}{m'_{F_{\gamma/K}}(-\lambda')} \cdot \frac{\alpha_K^2}{\alpha_K^2 + \gamma} = \frac{\gamma(1+r')^2 - K}{\gamma} \cdot \frac{\alpha_K^4}{\alpha_K^2 + \gamma}$$

and

$$(\mathbf{u}^E)^\top \mathcal{A}^{-1} \mathbf{u}^E = \frac{m^2_{F_\gamma}(-\lambda)\alpha_K^2}{m'_{F_\gamma}(-\lambda)} \cdot \frac{\alpha_K^2}{\alpha_K^2 + \gamma} = \frac{\gamma(1+r)^2 - 1}{\gamma} \cdot \frac{\alpha_K^4}{\alpha_K^2 + \gamma}$$

where we use the definitions of $D_{\rho,\widetilde{\gamma}}$ and $D_{\rho,\gamma,ind}$ for $\rho = 0$. Thus the pooled covariance matrix leads to the better estimator when

$$\gamma[(1+r')^2 - (1+r)^2] > K - 1.$$

■

Proof [Proof of Proposition 6.4] Let us first discuss the estimation of $\mathcal{Y}_{Kk'}$ for $k' \neq K$. By Theorem 1 of Serdobolskii (2007) we have the deterministic equivalence

$$(\widehat{\Sigma}_K + \lambda_K \mathbb{I}_p)^{-1} \asymp (x_p \Sigma_K + \lambda_K \mathbb{I}_p)^{-1}$$

for $x_p = x(\gamma_K, \lambda_K)$ as specified earlier. Thus

$$\begin{aligned}
&\operatorname{tr}[(\widehat{\Sigma}_K + \lambda_K \mathbb{I}_p)^{-1}(\widehat{\Sigma}_{k'} + \lambda_{k'} \mathbb{I}_p)^{-1}\Sigma_K]/p \\
=\ &\operatorname{tr}[(x_p\Sigma_K + \lambda_K \mathbb{I}_p)^{-1}(\widehat{\Sigma}_{k'} + \lambda_{k'} \mathbb{I}_p)^{-1}\Sigma_K]/p + \Omega_n \\
=\ &\frac{1}{px_p}\operatorname{tr}[(x_p\Sigma_K + \lambda_K \mathbb{I}_p)^{-1}(\widehat{\Sigma}_{k'} + \lambda_{k'} \mathbb{I}_p)^{-1}(x_p\Sigma_K + \lambda_K \mathbb{I}_p - \lambda_K \mathbb{I}_p)] + \Omega_n \\
=\ &\frac{1}{px_p}\operatorname{tr}[(\widehat{\Sigma}_{k'} + \lambda_{k'} \mathbb{I}_p)^{-1}] - \frac{\lambda_K}{px_p}\operatorname{tr}[(x_p\Sigma_K + \lambda_K \mathbb{I}_p)^{-1}(\widehat{\Sigma}_{k'} + \lambda_{k'} \mathbb{I}_p)^{-1}] + \Omega_n \\
=\ &\frac{1}{px_p}\operatorname{tr}[(\widehat{\Sigma}_{k'} + \lambda_{k'} \mathbb{I}_p)^{-1}] - \frac{\lambda_K}{px_p}\operatorname{tr}[(\widehat{\Sigma}_K + \lambda_K \mathbb{I}_p)^{-1}(\widehat{\Sigma}_{k'} + \lambda_{k'} \mathbb{I}_p)^{-1}] + \Omega_n
\end{aligned}$$

for a sequence $\Omega_n \to_{a.s.} 0$ as $p, n_k \to \infty$ with $p/n_k = \gamma_k$ for $k = 1, \ldots, K$. Here the first and last equalities follow by the deterministic equivalent for $(\widehat{\Sigma}_K + \lambda_K \mathbb{I}_p)^{-1}$ quoted above. For estimating $\mathcal{Y}_{kk'}$ where $k \neq K$ and $k' \neq K$, the result follows by the definition of $\widehat{\mathcal{Y}}_{kk'}$ and then using Lemma A.4 along with the independence of $\mathbf{X}_k$ for $k = 1, \ldots, K$. ■

A.2 Technical Lemmas and Their Proofs

Lemma A.4 *For a deterministic matrix $B \in \mathbb{R}^{p\times p}$ with operator norm $\|B\| \leq c$, the sample and population covariance matrices, $\widehat{\Sigma}_k$ and Σ_k, for $k = 1, \cdots, K$; satisfy:*

$$\operatorname{tr}(B(\widehat{\Sigma}_k - \Sigma_k))/p \to_{a.s.} 0.$$

Proof [Proof of Lemma A.4] Denoting the rows of $\mathbf{X}_k$ and $\mathbf{Z}_k$ by $X_{k,i}$ and $Z_{k,i} \in \mathbb{R}^p$ for $i = 1, \ldots, n_k$, we have $X_{k,i} - \mathbb{E}(X_{k,i}) = \Sigma_k^{1/2} Z_{k,i}$, and hence

$$\begin{aligned}\operatorname{tr}(B(\widehat{\Sigma}_k - \Sigma_k))/p &= \frac{1}{pn_k} \sum_{i=1}^{n_k} \operatorname{tr}(B((X_{k,i} - \mathbb{E}(X_{k,i}))(X_{k,i} - \mathbb{E}(X_{k,i}))^\top - \Sigma_k)) \\ &= \frac{1}{pn_k} \sum_{i=1}^{n_k} \left(Z_{k,i}^\top \Sigma_k^{1/2} B \Sigma_k^{1/2} Z_{k,i} - \mathbb{E}[Z_{k,i}^\top \Sigma_k^{1/2} B \Sigma_k^{1/2} Z_{k,i}] \right) \\ &= \frac{1}{pn_k} \sum_{i=1}^{n_k} T_{k,i}\end{aligned}$$

where for $i = 1, \ldots, n_k$,

$$T_{k,i} := Z_{k,i}^\top \Sigma_k^{1/2} B \Sigma_k^{1/2} Z_{k,i} - \mathbb{E}[Z_{k,i}^\top \Sigma_k^{1/2} B \Sigma_k^{1/2} Z_{k,i}]$$

are i.i.d. random variables with $\mathbb{E}T_{k,i} = 0$. By assumption 7, we have

$$\mathbb{E}[Z_{k,i}^\top \Sigma_k^{1/2} B \Sigma_k^{1/2} Z_{k,i}] = \operatorname{tr}(\Sigma_k^{1/2} B \Sigma_k^{1/2}).$$

We now compute the variance, writing $B' = \Sigma_k^{1/2} B \Sigma_k^{1/2}$ and using moment assumptions on Z from assumption 7:

$$\begin{aligned}&\operatorname{Var}(T_{k,i}) \\ =\ &\operatorname{Var}\left(\sum_{l_1, l_2} B'_{l_1 l_2} Z_{l_1} Z_{l_2} \right) \\ =\ &\sum_{l_1, l_2, l_3, l_4} \mathbb{E}(B'_{l_1 l_2} B'_{l_3 l_4} Z_{l_1} Z_{l_2} Z_{l_3} Z_{l_4}) - (\operatorname{tr}(B'))^2 \\ =\ &\sum_{l_1=1}^p \sum_{l_3=1}^p (B'_{l_1 l_1})(B'_{l_3 l_3}) + \sum_{l_1=1}^p \sum_{l_2=1}^p (B'_{l_1 l_2})^2 + \sum_{l_1=1}^p \sum_{l_2=1}^p (B'_{l_1 l_2})(B'_{l_2 l_1}) + \sum_{l_1=1}^p (B'_{l_1 l_1})^2 (\mathbb{E}Z_{l_1}^4 - 1) - (\operatorname{tr}(B'))^2 \\ =\ &2\|B'\|_{\mathrm{F}}^2 + \sum_{l_1=1}^p (B'_{l_1 l_1})^2 (\mathbb{E}Z_{l_1}^4 - 1) \le Cp\end{aligned}$$

for a constant $C > 0$. The last line follows since

$$\|B'\|_{\mathrm{F}}^2 \le p \|\Sigma_k^{1/2} B \Sigma_k^{1/2}\|^2 \le Cp$$

due to our assumption on $\|B\|$. Thus by Chebyshev inequality, for any $t > 0$, we have

$$\mathbb{P}\left(|*| \frac{1}{pn_k} \sum_{i=1}^{n_k} T_{k,i} > t \right) \le \frac{1}{p^2 n_k t^2} \operatorname{Var}(T_{k,i}) \le \frac{C}{pn_k t^2} = \frac{C}{\gamma_k n_k^2 t^2},$$

from where the almost sure convergence follows via Borel-Cantelli lemma. ■

Lemma A.5 *Under assumptions 3-2, consider a matrix $\mathbf{Z} \in \mathbb{R}^{p\times p}$ whose entries Z_{ij} have finite $(8+\epsilon)$-th moment for some $\epsilon > 0$. Suppose further that $\mathbf{Z}$ is independent of δ_k, $k, k' = 1, \cdots, K; k \neq k'$, we have as $p \to \infty$*

$$\delta_k^\top A\delta_{k'} - \rho_{kk'}\alpha_k\alpha_{k'} \operatorname{tr}(\mathbf{Z})/p \to_{a.s.} 0$$

$$\delta_k^\top A\delta_k - \alpha_k^2 \operatorname{tr}(\mathbf{Z})/p \to_{a.s.} 0$$

Proof [Proof of Lemma A.5] When $k = k'$, this lemma is the same as Lemma C.3 in Dobriban and Wager (2018) and theorem 2 in Sheng and Dobriban (2020). When $k \neq k'$, the same results still holds trivially under the bounded moments condition of A. This result has already been used by theorem 3.1 and theorem 4.1 of Zhao et al. (2023). ■

Lemma A.6 *Under the assumption 3, recall the definition of sample covariance matrix $\widehat{\Sigma} = (\mathbf{X} - \mathbf{1}_n\overline{X}^\top)^\top(\mathbf{X} - \mathbf{1}_n\overline{X}^\top)/n$ and its companion $\underline{\widehat{\Sigma}} = (\mathbf{X} - \mathbf{1}_n\overline{X}^\top)(\mathbf{X} - \mathbf{1}_n\overline{X}^\top)^\top/p$; we have*

$$\begin{aligned}
\operatorname{tr}[(\widehat{\Sigma} + \lambda\mathbb{I}_p)^{-1}]/p &\to_{a.s.} m_{F_\gamma}(-\lambda) \\
\operatorname{tr}[(\underline{\widehat{\Sigma}} + \lambda\mathbb{I}_p)^{-1}]/n &\to_{a.s.} v_{F_\gamma}(-\lambda) \\
\operatorname{tr}[(\widehat{\Sigma} + \lambda\mathbb{I}_p)^{-2}]/p &\to_{a.s.} m'_{F_\gamma}(-\lambda) \\
\operatorname{tr}[(\underline{\widehat{\Sigma}} + \lambda\mathbb{I}_p)^{-2}]/n &\to_{a.s.} v'_{F_\gamma}(-\lambda) \\
\operatorname{tr}[(\widehat{\Sigma} + \lambda\mathbb{I}_p)^{-1}\Sigma]/p &\to_{a.s.} \frac{1}{\gamma}\left(\frac{1}{\lambda v_{F_\gamma}(-\lambda)} - 1\right) \\
\operatorname{tr}[(\widehat{\Sigma} + \lambda\mathbb{I}_p)^{-2}\Sigma]/p &\to_{a.s.} \frac{1}{\gamma}\left(\frac{v_{F_\gamma}(-\lambda) - \lambda v'_{F_\gamma}(-\lambda)}{[\lambda v_{F_\gamma}(-\lambda)]^2}\right) \\
\operatorname{tr}[(\widehat{\Sigma} + \lambda\mathbb{I}_p)^{-2}\Sigma^2]/p &\to_{a.s.} \frac{1}{\gamma}\left(\frac{v'_{F_\gamma}(-\lambda) - v^2_{F_\gamma}(-\lambda)}{\lambda^2 v^4_{F_\gamma}(-\lambda)}\right).
\end{aligned}$$

Proof [Proof of Lemma A.6] The first four convergence statements follow from Marchenko and Pastur (1967) and Silverstein (1995). The convergence of last three trace terms are from Lemma 2 of Ledoit and Péché (2011), Lemma 2.2 of Dobriban and Wager (2018) and Lemma 3.11 of Dobriban and Wager (2018). ■

Lemma A.7 *Assume $n_1, \cdots, n_K, p \to \infty$, $p/n_k \to \gamma_k$ for $k = 1, \cdots, K$ and assumption 3. We have*

$$E_{kk'} := \operatorname{tr}[(\widehat{\Sigma}_k + \lambda_k\mathbb{I}_p)^{-1}(\widehat{\Sigma}_{k'} + \lambda_{k'}\mathbb{I}_p)^{-1}]/p \to_{a.s.} \mathcal{E}_{kk'}.$$

(1). Assume $n_1 = \cdots = n_K = n$, so $\gamma_1 = \cdots = \gamma_K = \gamma$, and use $\lambda_1 = \cdots = \lambda_K = \lambda$. Thus for all k, $m_{F_{\gamma_k}}(\lambda_k) = m_{F_\gamma}(\lambda)$. We have for $k \neq k'$

$$\mathcal{E}_{kk'} = \frac{(1-\gamma)m'_{F_\gamma}(-\lambda) + 2\gamma\lambda m_{F_\gamma}(-\lambda)m'_{F_\gamma}(-\lambda) - \gamma m_{F_\gamma}(-\lambda)^2}{1 - \gamma + \gamma\lambda^2 m'_{F_\gamma}(-\lambda)}.$$

(2). Under the assumption $\Sigma = \mathbb{I}_p$,

$$\mathcal{E}_{kk'} = m_{F_{\gamma_k}}(-\lambda_k) m_{F_{\gamma_{k'}}}(-\lambda_{k'}).$$

(3). Under the Assumptions 4 and 5, we have

$$\begin{aligned}\mathcal{E}_{kk'} &= \frac{1}{\lambda_k \lambda_{k'}} \Bigg\{ \lambda_k m_{F_{\gamma_k}}(-\lambda_k) + \lambda_{k'} m_{F_{\gamma_{k'}}}(-\lambda_{k'}) + \frac{\lambda_k m_{F_{\gamma_k}}(-\lambda_k) m_{F_{\gamma_{k'}}}(-\lambda_{k'})}{(m_{F_{\gamma_k}}(-\lambda_k) - m_{F_{\gamma_{k'}}}(-\lambda_{k'}))} \\ &\quad - \frac{\lambda_{k'} m_{F_{\gamma_k}}(-\lambda_k) m_{F_{\gamma_{k'}}}(-\lambda_{k'})}{(m_{F_{\gamma_k}}(-\lambda_k) - m_{F_{\gamma_{k'}}}(-\lambda_{k'}))} \Bigg\}.\end{aligned}$$

Proof [Proof of Lemma A.7] In the first case, when $n_1 = \cdots = n_K = n$ so $\gamma_1 = \cdots = \gamma_K = \gamma$ and use $\lambda_1 = \cdots = \lambda_K = \lambda$, the limits of term $E_{kk'}$ has been found in the proof theorem 3 in Sheng and Dobriban (2020). When $\Sigma = \mathbb{I}_p$, the term $E_{kk'}$ boils down to

$$\mathrm{tr}[(\widehat{\Sigma}_k + \lambda_k \mathbb{I}_p)^{-1} (\widehat{\Sigma}_{k'} + \lambda_{k'} \mathbb{I}_p)^{-1}]/p = \mathrm{tr}[(\mathbf{Z}_k^\top \mathbf{Z}_k / n_k + \lambda_k \mathbb{I}_p)^{-1} (\mathbf{Z}_{k'}^\top \mathbf{Z}_{k'} / n_{k'} + \lambda_{k'} \mathbb{I}_p)^{-1}]/p]$$

Note we always have

$$E_{kk'} \to_{a.s.} \mathbb{E}_H \frac{1}{(x_k T + \lambda_k)(x_{k'} T + \lambda_{k'})}$$

Recall H is the limiting population spectral distribution, and x_k is the fixed point solution to

$$1 - x_k = \gamma_k \left[1 - \lambda_k \int \frac{1}{x_k t + \lambda_k} dH(t) \right] \tag{18}$$

When $\Sigma = \mathbb{I}_p$, H only has a point mass on 1 so the expectation decomposes and

$$E_{kk'} \to_{a.s.} m_{F_{\gamma_k}}(-\lambda_k) m_{F_{\gamma_{k'}}}(-\lambda_{k'})$$

As an alternative proof when we have Assumption 4; we know $\mathbf{Z}_k$ will be asymptotically free from any bounded constant matrices, this is a standard result, ex. theorems 5.4.5 in Anderson et al. (2010). Further, we know sample covariances of the form $\mathbf{Z}_k^\top \mathbf{Z}_k / n_k$ is asymptotically free from $\mathbf{Z}_{k'}^\top \mathbf{Z}_{k'} / n_{k'}$ [see Capitaine and Casalis (2004)]. Two arguments combined suggests that $(\mathbf{Z}_k^\top \mathbf{Z}_k / n_k + \lambda_k \mathbb{I}_p)^{-1}$ is asymptotically free from $(\mathbf{Z}_{k'}^\top \mathbf{Z}_{k'} / n_{k'} + \lambda_{k'} \mathbb{I}_p)^{-1}$ therefore,

$$E_{kk'} - m_{F_{\gamma_k}}(-\lambda_k) m_{F_{\gamma_{k'}}}(-\lambda_{k'}) \to_{a.s.} 0$$

For the third case, a slight generalization of Corollary 3.9 of Knowles and Yin (2017) tells us

$$\ell_1^\top \left[(\widehat{\Sigma}_k + \lambda_k \mathbb{I}_p)^{-1} - \frac{1}{\lambda_k (1 + m_{F_{\gamma_k}}(-\lambda) \Sigma)} \right] \ell_2 \to_{a.s.} 0$$

where ℓ_1, ℓ_2 can be any continuous random vectors independent from $(\widehat{\Sigma}_k + \lambda_k \mathbb{I}_p)^{-1}$. We can decompose $E_{kk'}$ by

$$\underbrace{\mathrm{tr}[(\widehat{\Sigma}_k + \lambda_k \mathbb{I}_p)^{-1} (\widehat{\Sigma}_{k'} + \lambda_{k'} \mathbb{I}_p)^{-1}]/p}_{E_{kk'}} - \frac{1}{\lambda_k} \sum_{i=1}^{p} \ell_{1,i}^\top (\mathbb{I}_p + m_{F_{\gamma_k}}(-\lambda) \Sigma)^{-1} \ell_{2,i}/p \to_{a.s.} 0$$

where $\ell_{1,i}$ is e_i with 1 in its ith entry and 0 else where; and $\ell_{2,i} := (\widehat{\Sigma}_{k'} + \lambda_{k'}\mathbb{I}_p)^{-1}e_i$. From now on, simplify the notation by using $m_k := m_{F_{\gamma_k}}(-\lambda_k)$ and $m_{k'} := m_{F_{\gamma_{k'}}}(-\lambda_{k'})$. Perform the similar trick to $\ell_{2,i}$, we have

$$E_{kk'} - \frac{1}{\lambda_k\lambda_{k'}}\operatorname{tr}((\mathbb{I}_p + m_k\Sigma)^{-1}(\mathbb{I}_p + m_{k'}\Sigma)^{-1})/p \to_{a.s.} 0$$

In addition

$$\begin{aligned}
&\frac{1}{\lambda_k\lambda_{k'}}\operatorname{tr}((\mathbb{I}_p + m_k\Sigma)^{-1}(\mathbb{I}_p + m_{k'}\Sigma)^{-1})/p \\
=&\frac{1}{\lambda_k\lambda_{k'}}[1 - m_k\operatorname{tr}((\mathbb{I}_p + m_k\Sigma)^{-1}\Sigma)/p - m_{k'}\operatorname{tr}((\mathbb{I}_p + m_{k'}\Sigma)^{-1}\Sigma)/p \\
&+ m_km_{k'}\operatorname{tr}((\mathbb{I}_p + m_k\Sigma)^{-1}\Sigma(\mathbb{I}_p + m_{k'}\Sigma)^{-1}\Sigma)]/p
\end{aligned}$$

where we used the matrix identity

$$(\mathbb{I}_p + m_{F_{\gamma_k}}(-\lambda_k)\Sigma)^{-1} = \mathbb{I}_p - m_{F_{\gamma_k}}(-\lambda_k)(\mathbb{I}_p + m_k(-\lambda_k)\Sigma)^{-1}\Sigma$$

Each of the terms can be expressed in empirical quantities by

$$\operatorname{tr}((\mathbb{I}_p + m_k\Sigma)^{-1}\Sigma)/p - \mathbb{E}_H\frac{1}{m_k(1+tm_k)} + \mathbb{E}_H\frac{1}{m_k(1+tm_k)} - \frac{1}{m_k}[1 - \lambda_km_k] \to_{a.s.} 0$$

With the same techniques, we get

$$\begin{aligned}
&\operatorname{tr}((\mathbb{I}_p + m_k\Sigma)^{-1}\Sigma(\mathbb{I}_p + m_{k'}\Sigma)^{-1}\Sigma)/p \\
\to_{a.s.}&\frac{\lambda_km_k}{m_k(m_k - m_{k'})} - \frac{\lambda_{k'}m_{k'}}{m_{k'}(m_k - m_{k'})} + \frac{1}{m_km_{k'}}
\end{aligned}$$

Substitute these expressions back into the expressions for $E_{kk'}$ finishes the proof. ■

Lemma A.8 *With assumption 3, and under $n_k, p \to \infty$, $p/n_k \to \gamma_k$, we have the following convergence results*

$$\operatorname{tr}[(\widehat{\Sigma}_k + \lambda_k\mathbb{I}_p)^{-1}(\widehat{\Sigma}_{k'} + \lambda_{k'}\mathbb{I}_p)^{-1}\Sigma]/p \to_{a.s.} \mathcal{M}_{kk'}.$$

For $k \neq k'; k, k' \in \{1, \cdots, K\}$, we have:

1. *Assume $n_1 = \cdots = n_K = n$, so $\gamma_1 = \cdots = \gamma_K = \gamma$, and use $\lambda_1 = \cdots = \lambda_K = \lambda$.*

$$\mathcal{M}_{kk'} = \frac{m_{F_\gamma}(-\lambda) - \lambda m'_{F_\gamma}(-\lambda)}{1 - \gamma + \gamma\lambda^2 m'_{F_\gamma}(-\lambda)}.$$

2. *Under the assumption $\Sigma = \mathbb{I}_p$,*

$$\mathcal{M}_{kk'} = m_{F_{\gamma_k}}(-\lambda_k)m_{F_{\gamma_{k'}}}(-\lambda_{k'}).$$

3. Under Assumptions 4 and 5,

$$\mathcal{M}_{kk'} = \frac{\lambda_k m_{F_{\gamma_k}}(-\lambda_k) - \lambda_{k'} m_{F_{\gamma_{k'}}}(-\lambda_{k'})}{\lambda_k \lambda_{k'}(m_{F_{\gamma_{k'}}}(-\lambda_{k'}) - m_{F_{\gamma_k}}(-\lambda_k))}.$$

Proof [Proof of Lemma A.8] The proof is similar to the proof of lemma A.7. In the first case where $n_1 = \cdots = n_K = n$ so $\gamma_1 = \cdots = \gamma_K = \gamma$ and use $\lambda_1 = \cdots = \lambda_K = \lambda$, we have

$$\mathrm{tr}[(\widehat{\Sigma}_k + \lambda_k \mathbb{I}_p)^{-1}(\widehat{\Sigma}_{k'} + \lambda_{k'}\mathbb{I}_p)^{-1}\Sigma]/p \to_{a.s.} \int \frac{t}{(xt+\lambda)^2} dH(t)$$

When γ is equal across all populations, we will use the shorter notation $m := m_{F_\gamma}(-\lambda), m' = m'_{F_\gamma}(-\lambda)$ in this supplement. By definitions, we have

$$\int \frac{1}{xt+\lambda} dH(t) := m \text{ and } \int \frac{x't+1}{(xt+\lambda)^2} dH(t) := m'.$$

Here $x := x_k$ is the solution to the fixed point equation in (18), and x' is the derivative of x with respect to λ. Then

$$m' = \int \frac{(xt+\lambda-\lambda)\frac{x'}{x}+1}{(xt+\lambda)^2} dH(t) = \frac{x'}{x} m + (1 - \frac{\lambda x'}{x}) \int \frac{1}{(xt+\lambda)^2} dH(t)$$

$$\int \frac{1}{(xt+\lambda)^2} dH(t) = \frac{xm' - x'm}{x - \lambda x'}$$

So the functional of interest is

$$\begin{aligned} \int \frac{t}{(xt+\lambda)^2} dH(t) &= \frac{\int \frac{x't+1}{(xt+\lambda)^2} dH(t) - \int \frac{1}{(xt+\lambda)^2} dH(t)}{x'} \\ &= \frac{m' - \frac{xm'-x'm}{x-\lambda x'}}{x'} = \frac{\frac{m'x - \lambda m'x' - xm' + x'm}{x - \lambda x'}}{x'} \\ &= \frac{m - \lambda m'}{x - \lambda x'} = \frac{m - \lambda m'}{1 - \gamma + \gamma \lambda^2 m'}. \end{aligned}$$

For the second case, when $\Sigma = \mathbb{I}_p$, $\mathcal{M}_{kk'} = \mathcal{E}_{kk'}$. So the proof follows from lemma A.7. For the third case, pulling the trick with results from Knowles and Yin (2017) again gives

$$\mathrm{tr}(\Sigma(\widehat{\Sigma}_k + \lambda_k \mathbb{I}_p)^{-1}(\widehat{\Sigma}_{k'} + \lambda_{k'}\mathbb{I}_p)^{-1})/p \to_{a.s.} \frac{1}{\lambda_k \lambda_{k'}} \mathbb{E}_H \frac{t}{(1+tm_k)(1+tm_{k'})}$$

which can be consistently estimated by

$$\frac{1}{\lambda_k \lambda_{k'}} \frac{\lambda_k m_k - \lambda_{k'} m_{k'}}{m_{k'} - m_k}$$

as claimed by the lemma. ■

A.3 Proofs of Theorems

Proof [Proof of Theorem 4.1] Firstly, when $\pi_- = \pi_+$, we have

$$Err(\mathbf{w}) = \Phi\left(\frac{(\widehat{d}(\mathbf{w}))^\top \mu_{-1} + \widehat{b}_K}{\sqrt{(\widehat{d}(\mathbf{w}))^\top \Sigma(\widehat{d}(\mathbf{w}))}}\right)$$

Under Assumptions 1 and 2; by Lemma 3.7 in Dobriban and Wager (2018), we know $\widehat{b} \to_{a.s.} 0$. By Lemma 3.8 in the same paper, we can use the almost sure limit $(\widehat{d}(\mathbf{w}))^\top \mu_{-1} \to_{a.s.} (\widehat{d}(\mathbf{w}))^\top \delta_K$ and arrive at

$$Err(\mathbf{w}) \to_{a.s.} \Phi\left(\frac{(\widehat{d}(\mathbf{w}))^\top \delta_K}{\sqrt{(\widehat{d}(\mathbf{w}))^\top \Sigma(\widehat{d}(\mathbf{w}))}}\right).$$

Observe that

$$(\widehat{d}(\mathbf{w}))^\top \delta_K = \mathbf{w}^\top \widehat{\mathbf{u}}, \quad \widehat{\mathbf{u}} = vec\left[\widehat{\delta}_k(\widehat{\Sigma}_k + \lambda_k)^{-1}\delta_K\right]$$

$$(\widehat{d}(\mathbf{w}))^\top \Sigma(\widehat{d}(\mathbf{w})) = \mathbf{w}^\top \widehat{\mathcal{A}}\mathbf{w}, \quad \widehat{\mathcal{A}} = mat\left[\widehat{\delta}_k^\top(\widehat{\Sigma}_k + \lambda_k \mathbb{I}_p)^{-1}\Sigma(\widehat{\Sigma}_{k'} + \lambda_{k'}\mathbb{I}_p)^{-1}\widehat{\delta}_{k'}\right]$$

Here $vec[\cdot], mat[\cdot]$ are the vector operator and matrix operator respectively. As argued in the proof of Lemma 3.7 in Dobriban and Wager (2018), one can decompose $\widehat{\delta}_k$ in the kth population as

$$\widehat{\delta}_k = \delta_k + \frac{1}{\sqrt{n_k}}\Sigma^{1/2}\widetilde{\mathbf{Z}}_k$$

where $\widetilde{\mathbf{Z}}_k \in \mathbb{R}^p$ are standard normal random vectors independent of $\mathbf{X}_{k'}$ conditionally on $\mu_{\pm 1,k'}, \delta_{k'}$ for all $k' = 1, \cdots, K$. Thus, Lemma A.5 and Lemma A.6 gives us

$$\widehat{\mathbf{u}} \to_{a.s.} \rho_{kK}\alpha_k\alpha_K \; vec\left[m_{F_{\gamma_k}}(-\lambda_k)\right]$$

We decompose $\widehat{\mathcal{A}}$ into three parts for further analysis, such that

$$\widehat{\mathcal{A}}_{kk'} = \widehat{\delta}_k^\top(\widehat{\Sigma}_k + \lambda_k)^{-1}\Sigma(\widehat{\Sigma}_{k'} + \lambda_{k'})^{-1}\widehat{\delta}_{k'} = \widetilde{A}_{kk'} + 2\widetilde{B}_{kk'} + \widetilde{C}_{kk'}$$

where

$$\begin{aligned}
M^{(kk')} &:= (\widehat{\Sigma}_k + \lambda_k\mathbb{I}_p)^{-1}\Sigma(\widehat{\Sigma}_{k'} + \lambda_{k'}\mathbb{I}_p)^{-1} \\
\widetilde{A}_{kk'} &:= \delta_k^\top M^{(kk')}\delta_{k'} \\
\widetilde{B}_{kk'} &:= \delta_k^\top M^{(kk')}(\widehat{\delta}_{k'} - \delta_{k'}) \\
\widetilde{C}_{kk'} &:= (\widehat{\delta}_k - \delta_k)^\top M^{(kk')}(\widehat{\delta}_{k'}^\top - \delta_{k'})
\end{aligned}$$

Firstly, one can show $\widetilde{B}_{kk'} \to_{a.s.} 0$ with the same techniques used to prove $\widehat{b} \to_{a.s.} 0$. For the off-diagonal terms in $\widetilde{A}$, one can again invoke Lemma A.5 and Lemma A.6 to show

$$\widetilde{A}_{kk'} - \rho_{kk'}\alpha_k\alpha_{k'}\,\mathrm{tr}(M^{(kk')})/p \to_{a.s.} 0.$$

Next, the limit of $\mathrm{tr}(M^{(kk')})/p$ is provided by Lemma A.8. For the diagonal terms $\widetilde{A}_{kk}$, one can simply read off the limit of $\mathrm{tr}(M^{(kk)})/p$ from Lemma A.6. Finally, we have the following equality for $\widetilde{C}$ based on decomposing $\widehat{\delta}_k$:

$$\widetilde{C}_{kk'} = \frac{1}{n_k}\widetilde{Z}_k^\top \Sigma^{1/2} M^{(kk')} \Sigma^{1/2} \widetilde{Z}_{k'}$$

We know $\widetilde{C}_{kk'} \to_{a.s.} 0$ for $k \neq k'$ based on Lemma A.5 due to the independence between $\widetilde{Z}_k, \widetilde{Z}_{k'}$. For the diagonal terms of $\widetilde{C}$, we have

$$\widetilde{C}_{kk} - \gamma_k \,\mathrm{tr}(\Sigma M^{(kk)})/p \to_{a.s.} 0$$

and the limit of the trace of $\Sigma M^{(kk)}$ can again be read off from Lemma A.6. ■

Proof [Proof of Theorem 4.5] Recall the decomposition $\widehat{\delta}_k = \delta_k + \frac{1}{\sqrt{n_k}}\Sigma^{1/2}\widetilde{\mathbf{Z}}_k$, we then have from the objective in (3), that

$$\left\|\sum_{k=1}^{K} w_k(\widehat{\Sigma}_k + \lambda_k \mathbb{I}_p)^{-1}\widehat{\delta}_k - \Sigma^{-1}\delta_K\right\|_2^2 = \mathbf{w}^\top\left(\widehat{\mathcal{A}}^E + \widehat{\mathcal{R}}^E\right)\mathbf{w} - 2(\widehat{\mathbf{u}}^E)^\top \mathbf{w} + \|\delta_K^\top \Sigma^{-1}\|_2^2$$

where

$$\begin{aligned}
\widehat{\mathcal{A}}^E &= mat\left[\delta_k^\top(\widehat{\Sigma}_k + \lambda_k \mathbb{I}_p)^{-1}(\widehat{\Sigma}_{k'} + \lambda_{k'}\mathbb{I}_p)^{-1}\delta_{k'}\right]\\
\widehat{\mathcal{R}}^E &= mat\left[(\widehat{\delta}_k - \delta_k)^\top(\widehat{\Sigma}_k + \lambda_k \mathbb{I}_p)^{-1}(\widehat{\Sigma}_{k'} + \lambda_{k'}\mathbb{I}_p)^{-1}(\widehat{\delta}_{k'} - \delta_{k'})\right]\\
\widehat{\mathbf{u}}^E &= \delta_K^\top \Sigma^{-1} vec\left[(\widehat{\Sigma}_k + \lambda_k \mathbb{I}_p)^{-1}\delta_{k'}\right]
\end{aligned}$$

Taking the derivative with respect to $\mathbf{w}$, we get the finite-sample expression for optimal estimation weight

$$\widehat{\mathbf{w}}^E = \left(\widehat{\mathcal{A}}^E + \widehat{\mathcal{R}}^E\right)^{-1}\widehat{\mathbf{u}}^E$$

Taking $n, p \to \infty; p/n_k \to \gamma_k$, we can get the asymptotic expressions for each of the three terms above. Consider the linear term $\widehat{\mathbf{u}}^E$ first. By Lemma A.5

$$\widehat{\mathbf{u}}^E \to_{a.s.} \mathbf{u}^E = vec\left[\rho_{kK}\alpha_k\alpha_K \,\mathrm{tr}[\Sigma^{-1}(\widehat{\Sigma}_k + \lambda_k \mathbb{I}_p)^{-1}]/p\right]$$

Again, the *anisotropic local law* tells us that:

$$\mathrm{tr}(\Sigma^{-1}(\widehat{\Sigma}_{c,k} + \lambda_k \mathbb{I}_p)^{-1})/p \to_{a.s.} \frac{1}{\lambda_k}\mathbb{E}_H\left[\frac{1}{t(1+tm_k(-\lambda_k))}\right] = \frac{1}{\lambda_k}\mathbb{E}_H\left[\frac{1}{t} - \frac{m_k(-\lambda_k)}{1+tm_k(-\lambda_k)}\right]$$

$$\begin{aligned}
\mathrm{tr}(\Sigma^{-1}(\widehat{\Sigma}_{c,k} + \lambda_k \mathbb{I}_p)^{-1})/p &\to \frac{1}{\lambda_k}\mathbb{E}_H\left[\frac{1}{t[1+tm_k(-\lambda_k)]}\right]\\
&= \frac{1}{\lambda_k}\mathbb{E}_H\left[\frac{1}{t} - \frac{m_k(-\lambda_k)}{1+tm_k(-\lambda_k)}\right]
\end{aligned}$$

$$\to \frac{1}{\lambda_k} \operatorname{tr}(\Sigma^{-1})/p - m_k(-\lambda_k) \operatorname{tr}[(\widehat{\Sigma}_{c,k} + \lambda_k \mathbb{I}_p)^{-1})]/p.$$

For diagonal terms of $\widehat{\mathcal{A}}^E$, we have

$$\widehat{\mathcal{A}}_{kk}^{\mathbb{E}} \to_{a.s.} \alpha_k^2 m_{F_{\gamma_k}}(-\lambda_k)$$

For off diagonal terms of $\widehat{\mathcal{A}}^E$, we have

$$\widehat{\mathcal{A}}_{kk'}^{\mathbb{E}} \to_{a.s.} \rho_{kk'} \alpha_k \alpha_{k'} \mathcal{E}_{kk'}$$

where $\mathcal{E}_{kk'}$ can be found in Lemma A.7. Lastly, we firstly observe that the asymptotic off-diagonal terms of $\widehat{\mathcal{R}}^E$ are zero as $\widetilde{\mathbf{Z}}_k$ and $\widetilde{\mathbf{Z}}_{k'}$ are independent (see Lemma A.5). For diagonal terms of R^E, we have

$$\widehat{\mathcal{R}}_{kk}^E = \widetilde{\mathbf{Z}}_k^\top \Sigma^{1/2} (\widehat{\Sigma}_k + \lambda_k \mathbb{I}_p)^{-1} (\widehat{\Sigma}_k + \lambda_k \mathbb{I}_p)^{-1} \Sigma^{1/2} \widetilde{\mathbf{Z}}_k \to_{a.s.} \frac{v_{F_{\gamma_k}}(-\lambda_k) - \lambda_k v'_{F_{\gamma_k}}(-\lambda_k)}{\lambda_k v_{F_{\gamma_k}}(-\lambda_k)^2}$$

Gathering the asymptotic expressions together, along with the uniqueness of the asymptotic minimizer, we finish the proof. ■

Proof [Proof of Theorem 4.10] The proof is similar to the proof of Theorem 4.5. From the objective function in (4), we have

$$\mathbb{E}_{x_0} \left\| [\sum_{k=1}^K w_k (\widehat{\Sigma}_c + \lambda_k \mathbb{I}_p)^{-1} \widehat{\delta}_k - \Sigma^{-1} \delta_K]^\top x_0 \right\|_2^2 = \mathbf{w}^\top [\widehat{\mathcal{A}}^P + \widehat{\mathcal{R}}^P] \mathbf{w} - 2\mathbf{w}^\top \widehat{\mathbf{u}}^P + \|\delta_K^\top \Sigma^{-1/2}\|_2^2$$

where

$$\begin{aligned}
\widehat{\mathcal{A}}^P &= mat \left[\delta_k^\top (\widehat{\Sigma}_k + \lambda_k \mathbb{I}_p)^{-1} \Sigma (\widehat{\Sigma}_{k'} + \lambda_{k'} \mathbb{I}_p)^{-1} \delta_{k'} \right] \\
\widehat{\mathcal{R}}^P &= mat \left[(\widehat{\delta}_k - \delta_k)^\top (\widehat{\Sigma}_k + \lambda_k \mathbb{I}_p)^{-1} \Sigma (\widehat{\Sigma}_{k'} + \lambda_{k'} \mathbb{I}_p)^{-1} (\widehat{\delta}_{k'} - \delta_{k'}) \right] \\
\widehat{\mathbf{u}}^P &= vec \left[\delta_K^\top (\widehat{\Sigma}_k + \lambda_k \mathbb{I}_p)^{-1} \delta_{k'} \right]
\end{aligned}$$

We can again take the derivative with respect to $\mathbf{w}$ and set it to zero, we get

$$\widehat{\mathbf{w}}^P = \left(\widehat{\mathcal{A}}^P + \widehat{\mathcal{R}}^P \right)^{-1} \widehat{\mathbf{u}}^P$$

Taking $n, p \to \infty; p/n_k \to \gamma_k$, we can get the expression for each of the three terms above. Consider the linear term $\widehat{\mathbf{u}}^P$, by Lemma A.5

$$\widehat{\mathbf{u}}^P \to_{a.s.} vec \left[\rho_{kK} \alpha_k \alpha_K m_{F_{\gamma_k}}(-\lambda_k) \right].$$

For diagonal terms of $\widehat{\mathcal{A}}^P$, we have

$$\widehat{\mathcal{A}}_{kk}^P \to_{a.s.} \frac{\alpha_k^2}{\gamma_k} \frac{v_{F_{\gamma_k}}(-\lambda_k) - \lambda v'_{F_{\gamma_k}}(-\lambda_k)}{[\lambda v_{F_{\gamma_k}}(-\lambda_k)]^2}$$

For off diagonal terms of $\widehat{\mathcal{A}}^P$, we have

$$\widehat{\mathcal{A}}^P_{kk'} \to_{a.s.} \rho_{kk'}\alpha_k\alpha_{k'}\mathcal{M}_{kk'}.$$

The off-diagonal terms of $\widehat{\mathcal{R}}^P$ again converges to zero, and the diagonals are

$$\widehat{\mathcal{R}}^P_{kk} = \widetilde{\mathbf{Z}}_k^\top \Sigma\Sigma^{1/2}(\widehat{\Sigma}_k + \lambda_k\mathbb{I}_p)^{-1}(\widehat{\Sigma}_k + \lambda_k\mathbb{I}_p)^{-1}\Sigma^{1/2}\widetilde{\mathbf{Z}}_k \to_{a.s.} \frac{v_k'(-\lambda_k) - v_k^2(-\lambda_k)}{\lambda_k^2 v_k^4(-\lambda_k)}.$$

Gathering the asymptotic expressions together, along with the uniqueness of the asymptotic minimizer, we finish the proof. ■

Proof [Proof of Theorem 6.1] The proof is identical to that of Theorem 4.5 with minimal changes due to the differing covariance matrices Σ_k. ■

Proof [Proof of Theorem 6.3] The proof is identical to that of Theorem 4.10 with minimal changes due to the differing covariance matrices Σ_k. ■

A.4 Proofs of Corollaries

Proof [Proof of Corollary A.1] We follow the proof of Theorem 4.10 for the special case $\Sigma = \mathbb{I}_p$. Consequently,

$$\widehat{\mathcal{A}}^P_{kk'} = \delta_k^\top(\widehat{\Sigma}_k+\lambda_k\mathbb{I}_p)^{-1}\Sigma(\widehat{\Sigma}_{k'}+\lambda_{k'}\mathbb{I}_p)^{-1}\delta_{k'} \to_{a.s.} \begin{cases} \alpha_k^2 m_k'(-\lambda_k) & \text{if } k = k' \\ \rho_{kk'}\alpha_k\alpha_{k'}m_k(-\lambda_k)m_{k'}(-\lambda_{k'}) & \text{otherwise} \end{cases}$$

using the limits from Lemma A.6. The rest of the expressions follow similarly. ■

Appendix B. Numerical Experiments

B.1 Validation of Limiting Error Formulae

In this subsection, we use synthetic data to verify the accuracy of the theoretical formula in Theorems 4.1, 4.5, 4.10. We use a Toeplitz-type covariance matrix, $n_1, \cdots, n_K = 150, 140, 130, 120, 110, 100$ and $p = 150$. We use the same signal strength for all population $\alpha^2 = 0.5$ and the same weight correlation $\rho = 0.5$. The prediction error is based on 2000 simulated testing data points. We then vary λ_k from 0.3 to 10. For each λ_k, the simulation is repeated 50 times. One can refer to figure 6 and figure 7 for the comparison between simulated testing error rate (boxplots) and theoretical error rate (red line) for TL-RDA and TL-RDA respectively. We can see that the limiting formulae are very accurate across the λ_k considered.

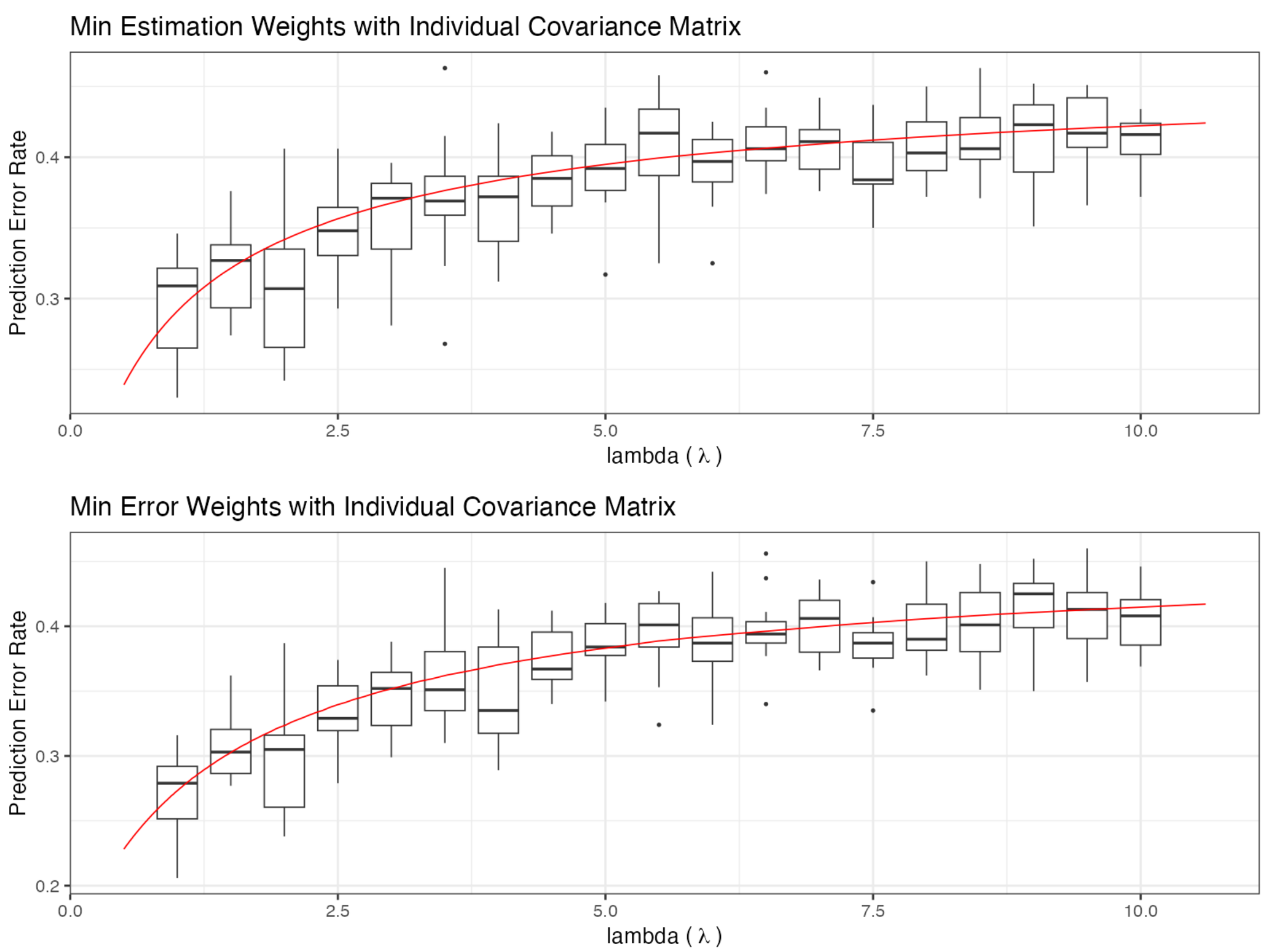

Figure 6: Individual sample covariance matrices

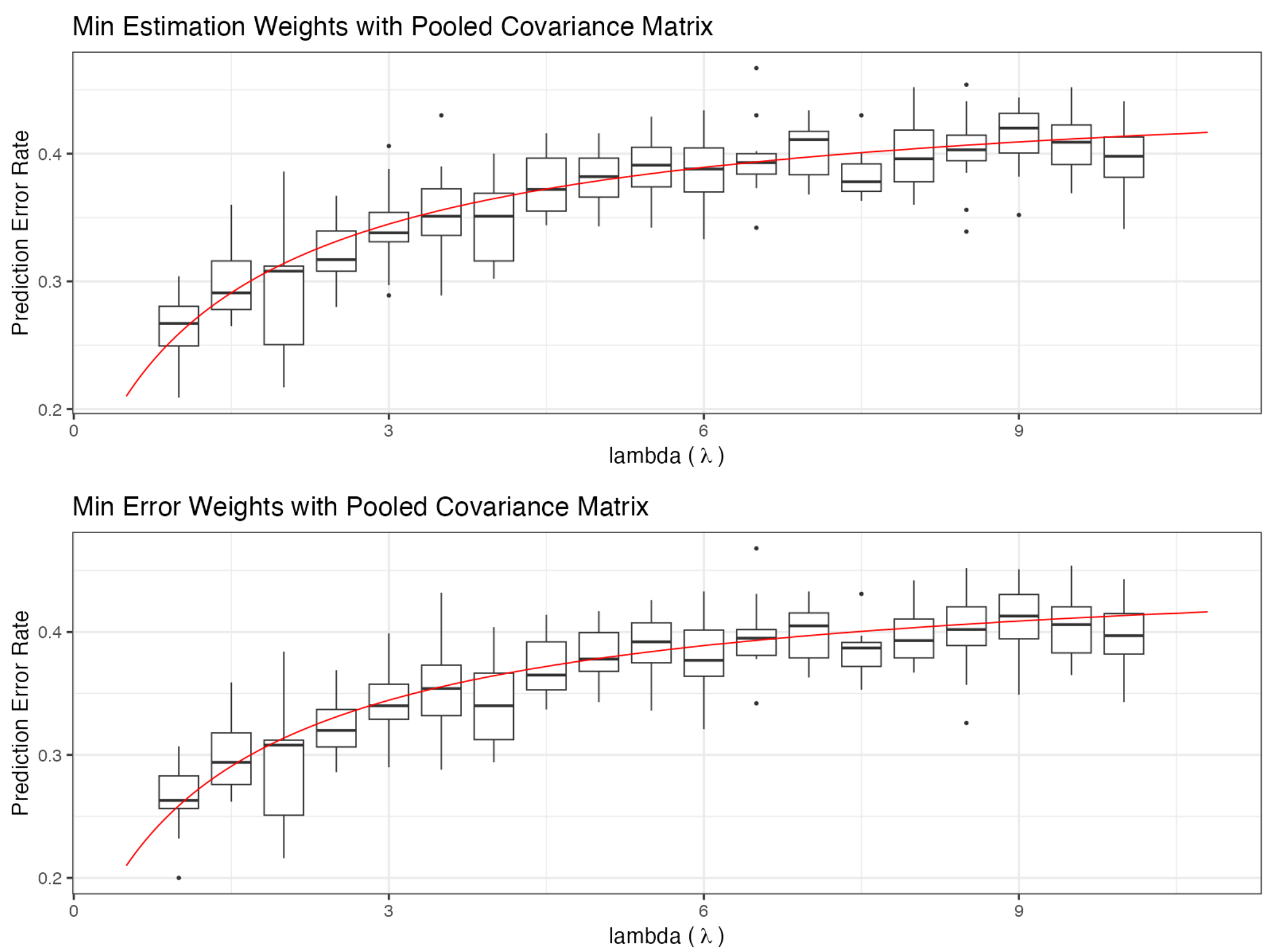

Figure 7: Pooled sample covariance matrices

B.2 Theoretical Error comparison

Here we plot the limiting errors of the naive RDA, TL-RDA with estimation weight and TL-RDA with prediction weight under different scenarios. Note the naive RDA is fitted on target population data only. One can refer to figure 8 and figure 9 for comparisons of TL-RDA and TLP-RDA respectively. We vary all parameters including correlation ρ, aspect ratio γ, signal strength α^2, number of populations K and population covariance matrix eigenvalues Σ. One can directly observe that TL estimators beat naive RDA in every set up considered, and not surprisingly, the prediction weight (minError) beats estimation weight (MinEst) in all set ups. Another interesting fact is estimation weight is usually not too much worse than prediction weight, especially in the TLP-RDA case. Lastly, we observe that the prediction errors turn to increase when γ_ks increase, decreases when α^2 increases, decrease when the number of populations increases. In addition, the error rate decreases when the eigenvalue of Σ decreases faster, that is, when the correlation strength between the features get stronger.

B.3 Estimators for Hyperparameters

We have assumed throughout this paper that $\alpha_k^2, \rho_{kk'}$ are known constants. We prove that the usual moment estimators, listed below, are consistent after debiasing.

$$\widehat{\alpha}_k^2 = \sum_{i=1}^{p} [(\widehat{\mu}_{+1,k})_i - (\widehat{\mu}_{-1,k})_i]^2/4 - \frac{1}{n}\text{trace}(\widehat{\Sigma}).$$

$$\widehat{\rho}_{kk'} = \sum_{i=1}^{p} [(\widehat{\mu}_{+1,k})_i - (\widehat{\mu}_{-1,k})_i][(\widehat{\mu}_{+1,k'})_i - (\widehat{\mu}_{-1,k'})_i]/4.$$

In order to prove the consistency of the estimators above, it is sufficient to prove proposition B.1 and proposition B.2.

Proposition B.1 (Variance of site mean) *For rows $X_1, \dots, X_n \in \mathbb{R}^p$ in X generated according to set up 1, assumption 1 and assumption 3 for a single class. Define the coordinate-wise sample means $\widehat{\mu}_j = \frac{1}{n}\sum_{i=1}^n X_{ij}$ and*

$$\widehat{\alpha}^2 := \sum_{j=1}^{p} \widehat{\mu}_j^2 - \frac{1}{n}\text{trace}(\widehat{\Sigma}).$$

Then

$$\mathbb{E}(\widehat{\alpha}^2) = \alpha^2.$$

Moreover, as $p, n \to \infty$ with $p/n \to \gamma$,

$$\widehat{\alpha}^2 \overset{\text{in probability}}{\longrightarrow} \alpha^2.$$

Proof Write $U = \widehat{\mu} - \mu$, so $U \mid \mu \sim \mathsf{N}(0, \Sigma/n)$ and $U \perp\!\!\!\perp \mu$. Then $\widehat{\mu} = \mu + U$, and for each j,

$$\mathbb{E}(\widehat{\mu}_j^2) = \text{Var}(\widehat{\mu}_j) = \mathbb{E}\big[\,\text{Var}(\widehat{\mu}_j \mid \mu)\big] + \text{Var}\left(\mathbb{E}[\widehat{\mu}_j \mid \mu]\right) = \frac{\Sigma_{jj}}{n} + \frac{\alpha^2}{p}.$$

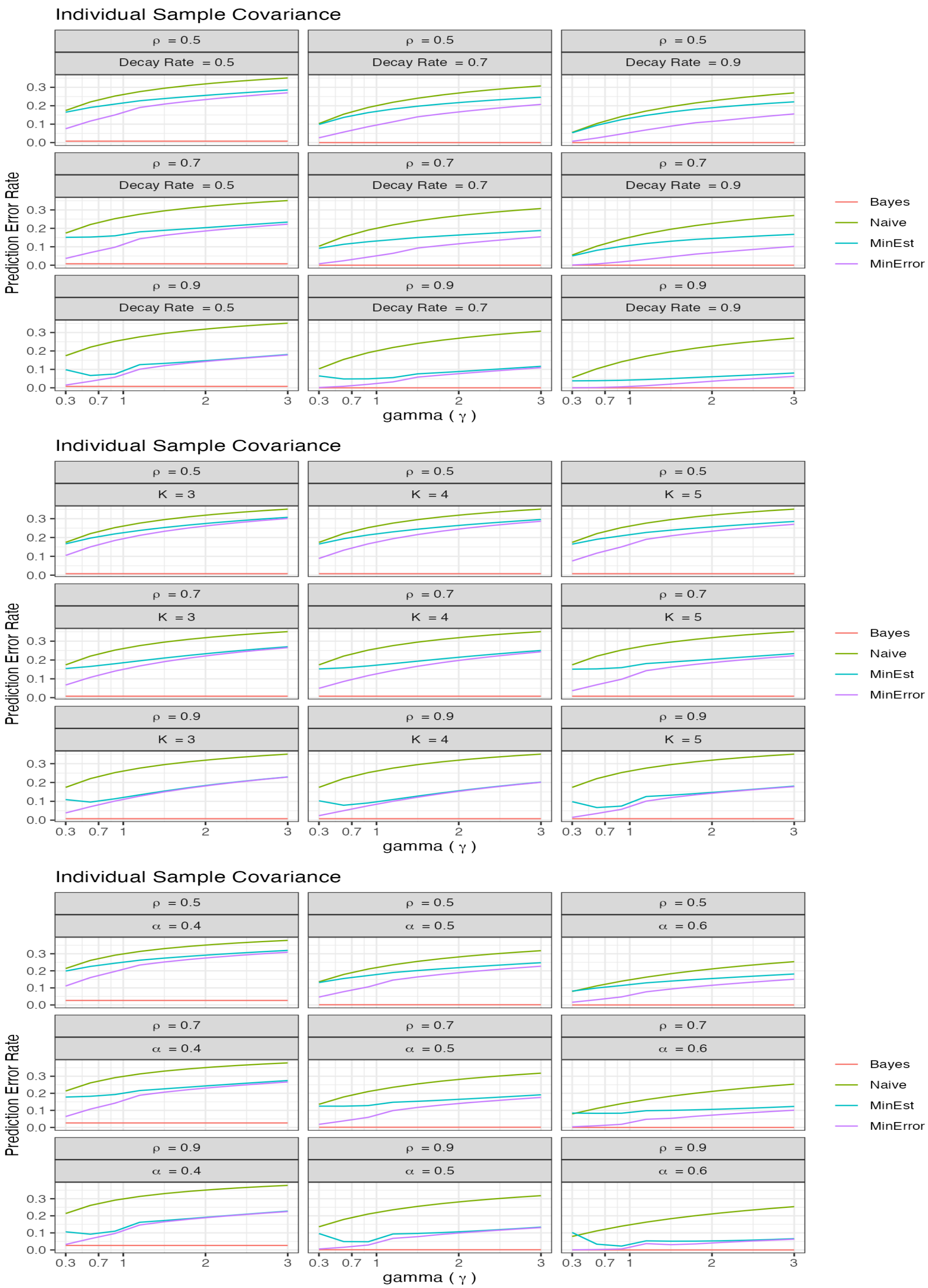

Figure 8: Individual sample covariance matrices

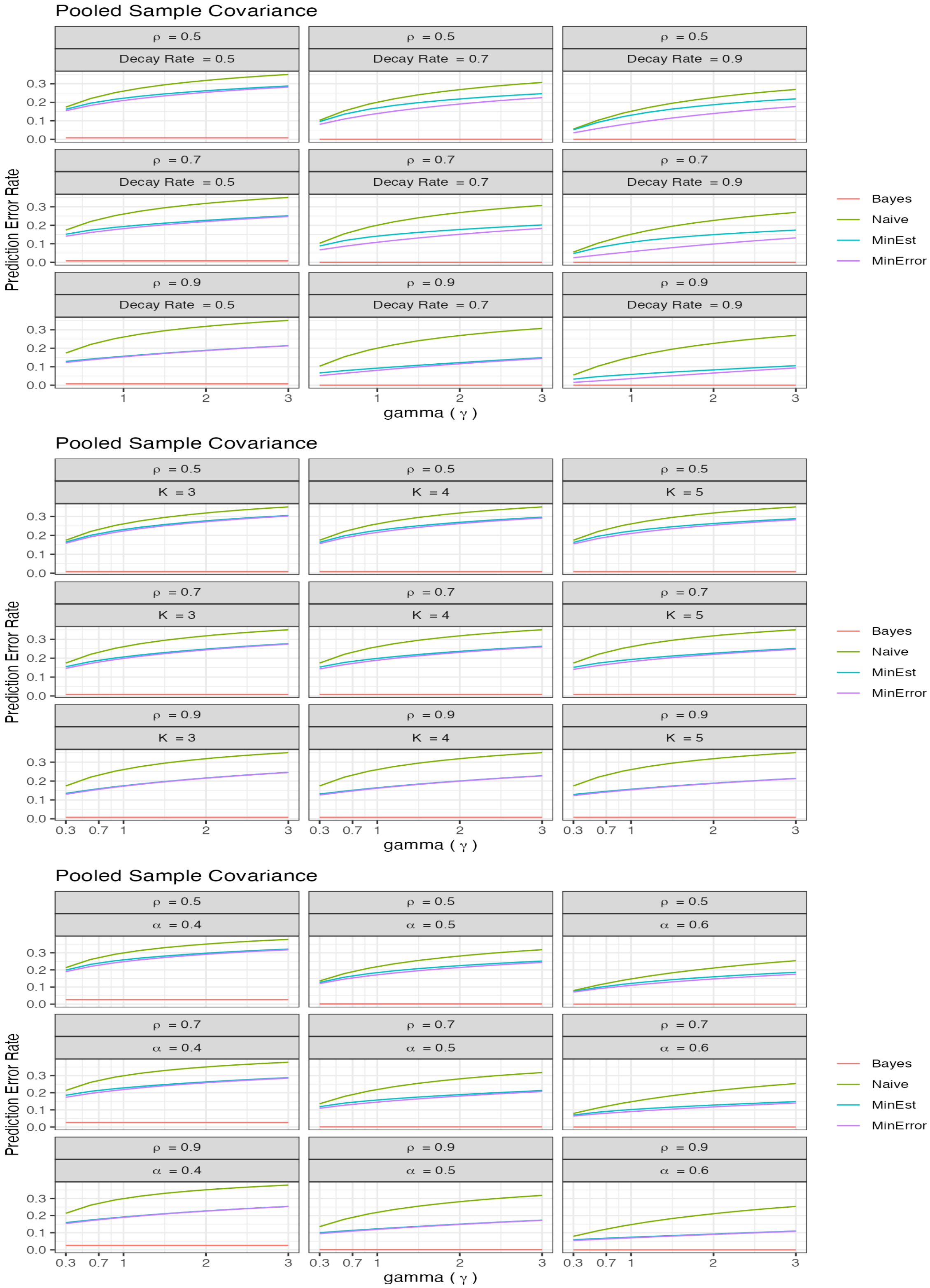

Figure 9: Pooled covariance matrices

Summing over j yields $\mathbb{E}(\widetilde{\alpha}^2) = \alpha^2$, where

$$\widetilde{\alpha}^2 = \sum_{j=1}^{p} \widehat{\mu}_j^2 - \mathrm{tr}(\Sigma)/n.$$

For the variance, expand

$$\mathrm{Var}(\widetilde{\alpha}^2) = \sum_{j=1}^{p} \mathrm{Var}(\widehat{\mu}_j^2) \;+\; 2 \sum_{1\le j<k\le p} \mathrm{Cov}(\widehat{\mu}_j^2, \widehat{\mu}_k^2).$$

Since $\widehat{\mu}$ is mean-zero and jointly Gaussian conditional on μ and the μ's are independent with mean zero, one can either compute directly via $U = \widehat{\mu} - \mu$ and independence, or invoke standard Gaussian fourth-moment identities (Isserlis/Wick), to obtain unconditionally

$$\mathrm{Var}(\widehat{\mu}_j^2) = 2\left(\frac{\Sigma_{jj}}{n} + \frac{\alpha^2}{p}\right)^2, \qquad \mathrm{Cov}(\widehat{\mu}_j^2, \widehat{\mu}_k^2) = \frac{2}{n^2}\Sigma_{jk}^2, \quad j \neq k.$$

Therefore,

$$\begin{aligned}
\mathrm{Var}(\widetilde{\alpha}^2) &= 2\sum_{j=1}^{p}\Big(\frac{\Sigma_{jj}}{n} + \frac{\alpha^2}{p}\Big)^2 + 4\sum_{1\le j<k\le p}\Big(\frac{\Sigma_{jk}}{n}\Big)^2 \\
&= 2\sum_{j=1}^{p}\Big(\frac{\Sigma_{jj}^2}{n^2} + \frac{2\alpha^2\,\Sigma_{jj}}{pn} + \frac{\alpha^4}{p^2}\Big) + \frac{2}{n^2}\sum_{j\neq k}\Sigma_{jk}^2 \\
&= \frac{2}{n^2}\|\Sigma\|_{\mathrm{F}}^2 + \frac{4\alpha^2}{pn}\,\mathrm{tr}\,\Sigma + \frac{2\alpha^4}{p}.
\end{aligned}$$

Specifically, assumption 3 upper bounds the eigenvalue of Σ, say with a constant C. Then as $p, n \to \infty$, $p/n \to \gamma$,

$$\frac{2}{n^2}\|\Sigma\|_F^2 \;\le\; \frac{2pC^2}{n^2} \;=\; O\Big(\frac{1}{n}\Big).$$

the second and third terms are $O((1/p)\cdot p/n) = O(p^{-1})$, , and $O(p^{-1})$ respectively. Hence $\mathrm{Var}(\widetilde{\alpha}^2) \to 0$, and Chebyshev yields $\widetilde{\alpha}^2$ converges in probability to α^2. To finish the proof we show that $\widehat{\alpha}^2 - \widetilde{\alpha}^2$ converges to zero in probability. Indeed

$$\widehat{\alpha}^2 - \widetilde{\alpha}^2 \;=\; -\frac{1}{n}\sum_{j=1}^{p}(\widehat{\sigma}_{jj} - \sigma_{jj})$$

For any fixed $j = 1, \ldots, p$, by standard results for $X_{ij} \overset{indep}{\sim} N(0, \sigma_{jj})$ for $i = 1, \ldots, n$ we have that $\widehat{\sigma}_{jj} - \sigma_{jj}$ converges to zero in probability. Averaging over j shows that $(\widehat{\alpha}^2 - \widetilde{\alpha}^2)$ also converges to zero and this finishes the proof. ■

Proposition B.2 (Correlation of site means) *Fix two sites k, k' with sample sizes $n_k, n_{k'}$ and dimension p. For $s \in \{k, k'\}$, let rows $X_1^{(s)}, \dots, X_{n_s}^{(s)} \in \mathbb{R}^p$ be generated under set up 1, assumption 1, assumption 2, assumption 3 for a single class, and the scalars $\alpha_k^2, \alpha_{k'}^2$ are known. Let $\widehat{\mu}_s = \frac{1}{n_s}\sum_{i=1}^{n_s} X_i^{(s)}$ be the sample mean for site s, and define*

$$\widehat{\rho}_{kk'} \;:=\; \frac{1}{\widehat{\alpha}_k\widehat{\alpha}_{k'}}\,\widehat{\mu}_k^\top\widehat{\mu}_{k'}.$$

where $\widehat{\alpha}_k$ are as defined in Proposition B.1. If as $p \to \infty$ we have $p/n_s \to \gamma_s \in (0,\infty)$,

$$\widehat{\rho}_{kk'} \overset{\textit{in probability}}{\longrightarrow} \rho_{kk'}.$$

Proof Let us define

$$\widetilde{\rho}_{kk'} \;:=\; \frac{1}{\alpha_k\alpha_{k'}}\,\widehat{\mu}_k^\top\widehat{\mu}_{k'}$$

by replacing $\widehat{\alpha}_k$ with their in probability limits α_k in $\widehat{\rho}_{kk'}$. Write $\widehat{\mu}_s = \mu_s + \varepsilon_s$ with $\varepsilon_s := \widehat{\mu}_s - \mu_s$. Conditional on μ_s, $\varepsilon_s \sim \mathsf{N}(0, \Sigma/n_s)$ and $\varepsilon_k, \varepsilon_{k'}$ are independent of $(\mu_k, \mu_{k'})$ and of each other (across sites). Using $\mathbb{E}[\varepsilon_s] = 0$ and cross-site independence of the noises,

$$\mathbb{E}\big[\widehat{\mu}_k^\top\widehat{\mu}_{k'}\big] = \mathbb{E}\big[\mu_k^\top\mu_{k'}\big] = \mathrm{tr}\big(\,\mathrm{Cov}(\mu_k, \mu_{k'})\big) = \alpha_k\alpha_{k'}\,\rho_{kk'}.$$

Dividing by $\alpha_k\alpha_{k'}$ yields $\mathbb{E}[\widetilde{\rho}_{kk'}] = \rho_{kk'}$. Expand

$$\widehat{\mu}_k^\top\widehat{\mu}_{k'} - \mathbb{E}[\widehat{\mu}_k^\top\widehat{\mu}_{k'}] = \underbrace{\big(\mu_k^\top\mu_{k'} - \mathbb{E}[\mu_k^\top\mu_{k'}]\big)}_{A} + \underbrace{\mu_k^\top\varepsilon_{k'}}_{B} + \underbrace{\mu_{k'}^\top\varepsilon_k}_{C} + \underbrace{\varepsilon_k^\top\varepsilon_{k'}}_{D}.$$

The four terms are mean-zero and pairwise uncorrelated (by independence across sites and Gaussian odd-moment cancellations), so variances add. A standard Gaussian fourth-moment computation gives

$$\mathrm{Var}(A) \;=\; \frac{\alpha_k^2\alpha_{k'}^2}{p}\,(1+\rho_{kk'}^2), \qquad \mathrm{Var}(B) \;=\; \frac{\alpha_k^2}{p\,n_{k'}}\;\mathrm{tr}(\Sigma), \qquad \mathrm{Var}(C) \;=\; \frac{\alpha_{k'}^2}{p\,n_k}\;\mathrm{tr}(\Sigma),$$

and, since $\varepsilon_k \perp \varepsilon_{k'}$ with covariances Σ/n_k and $\Sigma/n_{k'}$,

$$\mathrm{Var}(D) \;=\; \frac{1}{n_k n_{k'}}\;\mathrm{tr}(\Sigma^2).$$

Therefore

$$\mathrm{Var}(\widetilde{\rho}_{kk'}) = \frac{1}{\alpha_k^2\alpha_{k'}^2}\Big\{\frac{\alpha_k^2\alpha_{k'}^2}{p}(1+\rho_{kk'}^2) + \frac{\alpha_k^2}{p\,n_{k'}}\,\mathrm{tr}(\Sigma) + \frac{\alpha_{k'}^2}{p\,n_k}\,\mathrm{tr}(\Sigma) + \frac{1}{n_k n_{k'}}\,\mathrm{tr}(\Sigma^2)\Big\}.$$

Again, assumption 3 upper bounds the eigenvalue of Σ, say with a constant C. Then as $p, n_s \to \infty$, $p/n_s \to \gamma_s$,

$$\frac{1}{n_k n_{k'}}\;\mathrm{tr}(\Sigma^2) \;\le\; \frac{pC^2}{n_k n_{k'}} \;=\; O\bigg(\frac{1}{\min\{n_k, n_{k'}\}}\bigg)$$

and $\mathrm{tr}(\Sigma)/n_s = (\mathrm{tr}\,\Sigma/p)\cdot(p/n_s) = O(1)$, so each bracketed term is $o(1)$. Hence $\mathrm{Var}(\widetilde{\rho}_{kk'}) = O(p^{-1}) \to 0$, and Chebyshev yields $\widetilde{\rho}_{kk'} \overset{\text{in probability}}{\longrightarrow} \rho_{kk'}$. To complete the proof we use the consistency of $\widehat{\alpha}_k$ for α_k from Proposition B.1 and the continuous mapping theorem. ■

Appendix C. Limiting Error and Optimal Weight under Unequal Sampling

Recall the error rate under Assumption 1 is

$$Err(\mathbf{w}) = \pi_-\Phi\left(\frac{\widehat{d}(\mathbf{w})^\top \mu_{-1} + \widehat{b}_K}{\sqrt{\widehat{d}(\mathbf{w})^\top \Sigma \widehat{d}(\mathbf{w})}}\right) + \pi_+\Phi\left(-\frac{\widehat{d}(\mathbf{w})^\top \mu_1 + \widehat{b}_K}{\sqrt{\widehat{d}(\mathbf{w})^\top \Sigma \widehat{d}(\mathbf{w})}}\right)$$

Now the stochastic representations become

$$\widehat{\delta}_k = \delta_k + \Sigma^{1/2}\left[\frac{\widetilde{\mathbf{Z}}_{k,+1}}{\sqrt{n_{k,+1}}} - \frac{\widetilde{\mathbf{Z}}_{k,-1}}{\sqrt{n_{k,-1}}}\right]$$

$$\widehat{\mu}_k = \bar{\mu}_k + \Sigma^{1/2}\left[\frac{\widetilde{\mathbf{Z}}_{k,+1}}{\sqrt{n_{k,+1}}} + \frac{\widetilde{\mathbf{Z}}_{k,-1}}{\sqrt{n_{k,-1}}}\right]$$

The intercept terms are no longer zeros and

$$\begin{aligned}
\widehat{b}_k =& -\widehat{\delta}_k^\top(\widehat{\Sigma}_k + \lambda_k \mathbb{I}_p)^{-1}(\widehat{\mu}_{-1,k} + \widehat{\mu}_{+1,k})/2 \\
=&\frac{1}{4n_{k,-1}}\widetilde{\mathbf{Z}}_{k,-1}^\top \Sigma^{1/2}(\widehat{\Sigma}_k + \lambda_k \mathbb{I}_p)^{-1}\Sigma^{1/2}\widetilde{\mathbf{Z}}_{k,-1} - \frac{1}{4n_{k,+1}}\widetilde{\mathbf{Z}}_{k,+1}^\top \Sigma^{1/2}(\widehat{\Sigma}_k + \lambda_k \mathbb{I}_p)^{-1}\Sigma^{1/2}\widetilde{\mathbf{Z}}_{k,+1} \\
\to_{a.s.}& \frac{\gamma_{k,-1} - \gamma_{k,+1}}{4\gamma_k}\left(\frac{1}{\lambda_k v_{F_{\gamma_k}}(-\lambda_k)} - 1\right)
\end{aligned}$$

Where we use $p/n_{k,\pm 1} \to \gamma_{k,\pm 1}$. For the numerator we have

$$\widehat{d}(\mathbf{w})^\top \mu_{\pm 1} = \mathbf{w}^\top vec\left[(\bar{\mu}_k \pm \widehat{\delta}_k)^\top(\widehat{\Sigma}_k + \lambda_k)^{-1}\delta_K\right] \to_{a.s.} \mathbf{w}^\top vec\left[\pm\rho_{kK}\alpha_k\alpha_K m_{F_{\gamma_k}}(-\lambda_k)\right]$$

For the denominator term,

$$\widehat{d}(\mathbf{w})^\top \Sigma \widehat{d}(\mathbf{w}) = \mathbf{w}^\top mat\left[\widehat{\delta}_k^\top(\widehat{\Sigma}_k + \lambda_k)^{-1}\Sigma(\widehat{\Sigma}_{k'} + \lambda_{k'})^{-1}\widehat{\delta}_{k'}\right]\mathbf{w} := \mathbf{w}^\top S \mathbf{w}.$$

For $k \neq k'$

$$S_{kk'} \to_{a.s.} \alpha_k^2 \frac{v_{F_{\gamma_k}}(-\lambda) - \lambda v'_{F_{\gamma_k}}(-\lambda)}{\gamma_k[\lambda v_{F_{\gamma_k}}(-\lambda)]^2} + \frac{\gamma_{k,-1} + \gamma_{k,+1}}{4}\frac{v'_{F_{\gamma_k}}(-\lambda) - v^2_{F_{\gamma_k}}(-\lambda)}{\lambda^2 v^4_{F_{\gamma_k}}(-\lambda)}$$

while

$$S_{kk} \to_{a.s.} \rho_{kk'}\alpha_k\alpha_{k'}\frac{\mathrm{tr}(M^{kk'})/p}{\gamma_k} + \frac{\gamma_{k,-1} + \gamma_{k,+1}}{4}\frac{v'_{F_{\gamma_k}}(-\lambda) - v^2_{F_{\gamma_k}}(-\lambda)}{\lambda^2 v^4_{F_{\gamma_k}}(-\lambda)}$$

for $1 \leq k, k' \leq K$. Again the limit of $\mathrm{tr}(M^{kk'})/p$ can be found in Lemma A.8.

As for the optimal weights, we know the Bayes optimal prediction is

$$d_{Bayes}(x_0) = \delta_K^\top \Sigma^{-1} x_0 + \delta_K^\top \Sigma^{-1}\bar{\mu}_K + \log\left(\frac{\pi_{K,+}}{\pi_{K,-}}\right).$$

Note δ_K and $\bar{\mu}_K$ are independent by Assumption 1, therefore by Lemma A.5 the second term is zero. In this case, we recommend an estimator in the form of $\sum_{k=1}^{K} \widehat{d}_k + \log(\pi_{K,+}/\pi_{K,-})$ whose intercept term is consistent with the one of the Bayes direction. Further, one can still obtain the optimal weights that minimize criteria (3) and (4). However, the proposition 4.14 no longer holds and the prediction weight is not guaranteed to minimize the testing data error rate. The error weight on testing data has the following expression.

$$\pi_{K,-}\Phi\left(\frac{\widehat{d}(\mathbf{w})^\top \mu_{K,-1}}{\sqrt{\widehat{d}(\mathbf{w})^\top \Sigma \widehat{d}(\mathbf{w})}} + \frac{\widehat{b}_K}{\sqrt{\widehat{d}(\mathbf{w})^\top \Sigma \widehat{d}(\mathbf{w})}}\right) + \pi_{K,+}\Phi\left(-\frac{\widehat{d}(\mathbf{w})^\top \mu_{K,1}}{\sqrt{\widehat{d}(\mathbf{w})^\top \Sigma \widehat{d}(\mathbf{w})}} - \frac{\widehat{b}_K}{\sqrt{\widehat{d}(\mathbf{w})^\top \Sigma \widehat{d}(\mathbf{w})}}\right)$$

The difficulty arises as $\widehat{b}_K$ is now non-zero, and merely maximizing the first fraction, as the optimal prediction weight does, is no longer sufficient. When K is low, one can potentially search for a local or even global optimal point for $\mathbf{w}$ with numeric methods.

Expected Prediction Error

For any linear classifier $\text{sign}(\mathbf{w}^\top x_0 + b)$, we can write its prediction error rate as followed

$$\begin{aligned}
&\mathbb{P}(\text{sign}(\mathbf{w}^\top x_0 + b) \neq y_0) \\
&= \pi_- \mathbb{P}(\text{sign}(\mathbf{w}^\top x_0 + b) \neq -1 | y_0 = -1) + \pi_+ \mathbb{P}(\text{sign}(\mathbf{w}^\top x_0 + b) \neq 1 | y_0 = 1)
\end{aligned}$$

The expressions for the two parts above are symmetric, so we can only look the first part.

$$\begin{aligned}
&\mathbb{P}(\text{sign}(\mathbf{w}^\top x_0 + b) \neq -1 | y_0 = -1) \\
&= \mathbb{P}(\mathbf{w}^\top \mu_{-1} + \mathbf{w}^\top \epsilon + b > 0) \\
&= \mathbb{P}(\mathbf{w}^\top \epsilon \leq \mathbf{w}^\top \mu_{-1} + b) = \Phi\left(\frac{\mathbf{w}^\top \mu_{-1} + b}{\sqrt{\mathbf{w}^\top \Sigma \mathbf{w}}}\right)
\end{aligned}$$
